# A Short Synthesis of Aphanamol I in Both Racemic and Enantiopure Forms

**DOI:** 10.1002/chem.201602669

**Published:** 2016-07-08

**Authors:** Steven J. Ferrara, Jonathan W. Burton

**Affiliations:** ^1^ Department of ChemistryUniversity of OxfordChemistry Research LaboratoryMansfield RoadOxfordOX1 3TAUK

**Keywords:** aphanamol, Claisen rearrangement, conjugate addition, radical cyclization, total synthesis

## Abstract

A short synthesis of the biologically active sesquiterpene natural product (+)‐aphanamol I in both racemic and enantiopure forms is reported. Key steps include: a catalytic enantioselective conjugate addition, an oxidative radical cyclization, and a ring‐expanding Claisen rearrangement.

(+)‐Aphanamol I **1** is a sesquiterpene natural product isolated as one of the minor toxic principles from the fruit peel of the timber tree *Aphanamixis grandifolia* by Nishizawa and co‐workers.^1^, ^2^ (+)‐Aphanamol I contains a core bicyclo[5.3.0]decane (hydroazulene) a common structural motif embedded in a large number of terpenoid natural products.^3^ There have been a number of notable syntheses of aphanamol I from the groups of Mehta,^4^, ^5^ Wickberg,^6^ Harmata^7^ and Wender.^8^, ^9^ To date all of the asymmetric syntheses of aphanamol I have used limonene as the chiral pool starting material, and featured various key steps, including: a diastereoselective acyclic Claisen rearrangement and an enone‐olefin cyclization,^4^, ^5^ a photochemical cycloaddition followed by a Grob‐type fragmentation,^6^ and a rhodium‐catalyzed [5+2] cycloaddition of an allene with a vinylcyclopropane,^8^ whereas Harmata's synthesis of racemic aphanamol I featured a key [4+3] allyl cation/diene cycloaddition to construct the core bicyclo[5.3.0]decane.^7^ Herein we report a short and highly efficient catalytic enantioselective synthesis of (+)‐aphanamol I, which features a catalytic asymmetric conjugate addition of an acetylene to an α,β‐unsaturated aldehyde, an oxidative γ‐lactone annulation and a ring‐expanding Claisen rearrangement as key steps.^10^, ^11^, ^12^ This strategy provides rapid access to the key [5.3.0]‐bicyclic decane structural motif from which the natural product was readily prepared and provides a platform for the synthesis of other hyrdoazulene natural products.

Embedded within the carbon framework of aphanamol I **1** is the retron for the Claisen rearrangement.^13^ Application of this retrosynthetic transformation leads to the [3.3.0]‐bicyclic enol ether **2**, which would be readily prepared from the corresponding [3.3.0]‐bicyclic lactone **3** following methylenation (Figure 1).^12^ We have recently used oxidative radical methodology for the synthesis of [3.3.0]‐bicyclic lactones by the cyclization of 4‐pentenyl malonates.^14^, ^15^, ^16^, ^17^, ^18^, ^19^ Application of such an oxidative radical cyclization to an appropriately functionalized 4,6‐heptadienyl malonate **4** should yield the corresponding alkenyl‐substituted [3.3.0]‐bicyclic lactone **3**. Based on the Beckwith–Houk model^20^, ^21^ for 5‐*exo*‐trig radical cyclizations and our own previous experience, we would predict that the oxidative radical cyclization would proceed through the pre‐transition state assembly **5** with the *iso*‐propyl group residing in a *pseudo‐*equatorial position of the chair‐like transition state with minimization of allylic strain. Preparation of the dienyl malonate such as **4** in enantioenriched form was to be achieved using the beautiful catalytic enantioselective conjugate addition methodology recently reported by Nishimura and Hayashi,^22^, ^23^ with the requisite diene being formed by a hydroboration–Suzuki cross‐coupling sequence.

**Figure 1:**
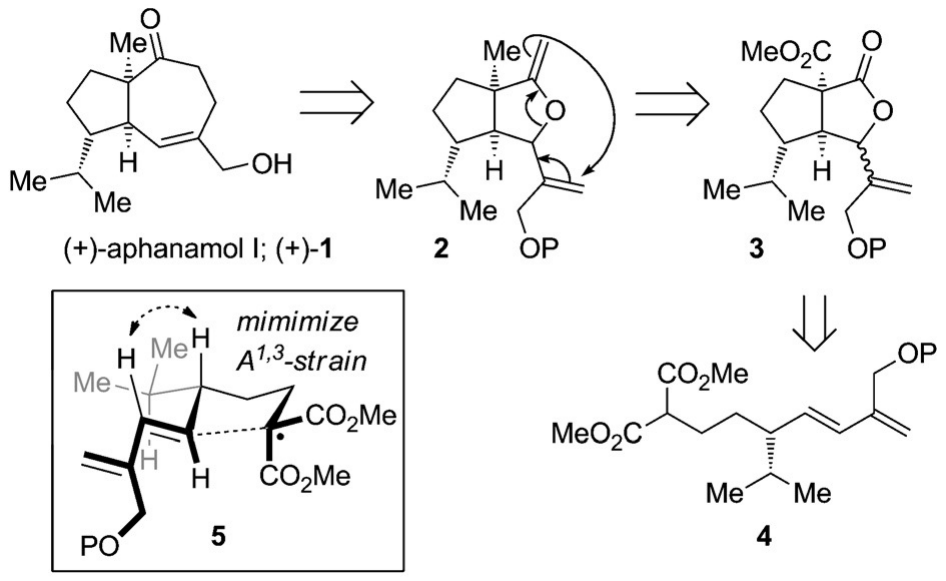
Retrosynthetic analysis of (+)‐aphanamol I (+)‐**1**; P=protecting group.

Our initial studies focused on developing a synthesis of aphanamol I in racemic form so that we could determine the effectiveness of the previously unreported oxidative radical cyclization of 4,6‐heptadienyl malonates for the synthesis of vinyl‐substituted [3.3.0]‐bicyclic lactones. The synthetic route to racemic aphanamol I (±)‐**1** began with the conjugate addition of (trimethylsilyl)acetylene **7** to the unsaturated malonate **6** 24 to give (±)‐**8** in 94 % yield using the procedure of Ohno and Tanaka^25^ (Scheme 1). Krapcho decarboxylation of the malonate (±)‐**8** 26 gave the ester (±)‐**9**, which, on reduction with lithium aluminum hydride, provided the primary alcohol (±)‐**10** in good yield. The alcohol (±)‐**10** was converted into the corresponding tosylate and the alkyne protecting group was removed using tetra‐*n*‐butylammonium fluoride giving (±)‐**11**. Hydroboration of the terminal alkyne in (±)‐**11** with catechol borane followed by Suzuki–Miyaura cross‐coupling under standard conditions using the readily prepared iodide **12** 27 gave the diene (±)‐**13** in 79 % yield.^28^ Alkylation of dimethyl malonate with the tosylate (±)‐**13** gave the cyclization substrate (±)‐**14**. After brief optimization we found that exposure of the dienyl malonate (±)‐**14** to our usual oxidative radical cyclization conditions, manganese(III) acetate and copper(II) triflate in acetonitrile, delivered the [3.3.0]‐bicyclic γ‐lactones (±)‐**15** in 79 % yield (Scheme 2).^29^ The lactones (±)‐**15** were isolated as a 6:1 mixture of inseparable C‐1 diastereomers.^30^, ^31^ The oxidative radical cyclization most likely takes place via the pre‐transition state assembly **5** (P=OTBDPS) with the *iso*‐propyl group and the diene occupying *pseudo*‐equatorial positions in the chair‐like transition state. The adduct allylic radical so formed then undergoes oxidative lactonization to deliver the product as a mixture of diastereomers at the lactone stereocenter. Krapcho decarboxylation^26^ of (±)‐**15** gave the corresponding lactones (±)‐**16**, which could be separated by flash chromatography and were individually characterized allowing assignment of their relative configurations by ^1^H NMR NOE experiments. Alkylation of the major diastereomer (±)‐**16 a** was readily achieved using methyl iodide and lithium bis(trimethylsilyl)amide giving the γ‐lactone (±)‐**17**. The alkylated lactone (±)‐**17** was readily converted into the corresponding *exo*‐cyclic enol ether (±)‐**18** on exposure to dimethyltitanocene in toluene at reflux.^32^ Heating the enol ether (±)‐**18** in xylene at reflux induced the desired Claisen rearrangement to provide the two‐carbon ring‐expanded product (±)‐**19** in 76 % yield. Deprotection of the silyl ether provided aphanamol I in racemic form (86 %). The ^1^H and ^13^C NMR data of our synthetic material were in excellent with agreement with that of the natural product^1^ and previously reported data on synthetic samples.^6^, ^8^

**Scheme 1:**
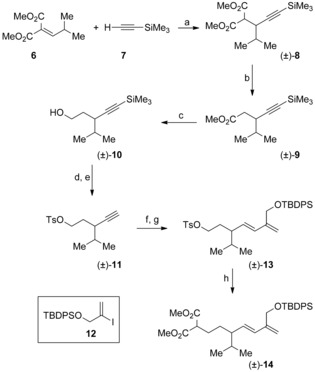
Synthesis of cyclization substrate (±)‐**14**. a) CH_3_CH_2_MgBr, 0.1 mol % CuCl, THF, 0 °C–RT, 94 %; b) LiCl, water, DMF, 150 °C, 81 %; c) LiAlH_4_, Et_2_O, 0 °C–RT, 93 %; d) 1 m Bu_4_NF, THF, RT, 92 %; e) *p‐*CH_3_C_6_H_4_SO_2_Cl, pyridine, CH_2_Cl_2,_ RT, 87 %; f) catecholborane, THF, 70 °C 18 h; g) **12**, 5 mol % Pd(OAc)_2_, 20 mol % PPh_3_, 2 m LiOH, THF, 40 °C, 4 h, 79 % (two steps); h) CH_2_(CO_2_CH_3_)_2_, NaH, DMF, THF, 80 °C, 1.5 h, 76 %. TBDPS=*tert*‐butyldiphenylsilyl, THF=tetrahydrofuran, DMF=dimethylformamide.

**Scheme 2:**
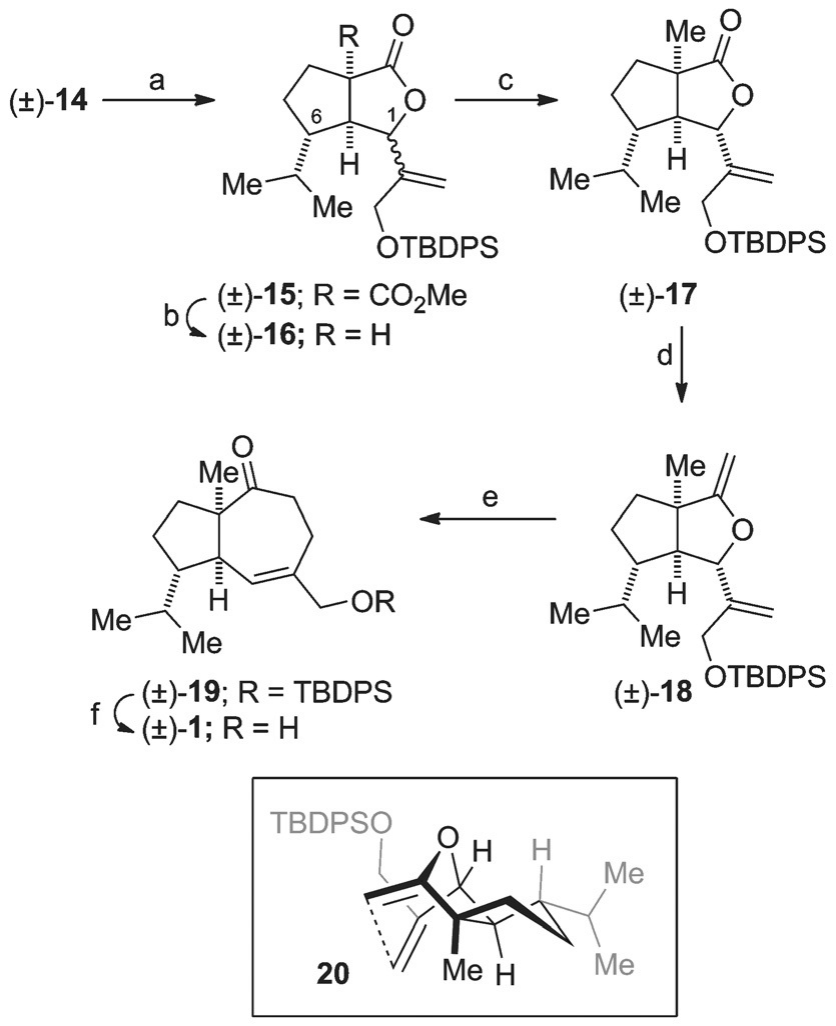
Synthesis of aphanamol I in racemic form. a) Manganese(III) acetate, copper(II) triflate, acetonitrile, reflux, 89 %, 6:1 d.r. at C‐1; b) LiCl, water, DMF, 150 °C, 65 % (±)‐**16 a** major diastereomer, 8 % (±)‐**16 b** minor diastereomer; c) CH_3_I, ((CH_3_)_3_Si)_2_NLi, THF, −78 °C, 90 %; d) Cp_2_Ti(CH_3_)_2_, toluene, reflux, 89 %; e) xylene, reflux, 76 %; f) Bu_4_NF, THF, 86 %. Cp=cyclopentadienyl.

The Claisen rearrangement to form the [5.4.0]‐bicyclic ketone (±)‐**19** is precedented from the work of Haramata and most likely proceeds through a concerted [3,3]‐sigmatropic rearrangement from a chair‐like pre‐transition state assembly related to that depicted in Scheme 2 (**20**) with the isopropyl group occupying a pseudo‐equatorial position.^33^, ^34^

Having established a thirteen‐step route to aphanamol I in racemic form, we sought to develop a synthesis of the natural product in enantioenriched form. This was reduced to the preparation of the cyclization substrate (**14**) in enantioenriched from. Nishimura, Hiyashi, and co‐workers recently reported a beautiful enantioselective rhodium‐catalyzed conjugate addition of (triisopropylsilyl)acetylene to α,β‐unsaturated aldehydes to give β‐alkynylated aldehydes in high yields and enantiomeric excesses.^22^ They had prepared the enantiomer of the aldehyde (−)‐**23** (Scheme 3) in 88 % yield and 99 % *ee* on a 0.2 mmol scale. Following the reported procedure,^22^ but using (*S*)‐DTBM‐segphos in place of (*R*)‐DTDM‐segphos, as well as working up the reaction with sodium borohydride, gave the alcohol (−)‐**24** in 78 % yield and 99 % *ee* on a 7.0 mmol scale.^35^ A similar synthetic route was used to convert the alkyne (−)‐**24** into (+)‐aphanamol I (+)‐(**1**) with similar yields. Thus, the alcohol (−)‐**24** was readily converted into the enantiopure cyclization substrate (+)‐**14** 36 using a Suzuki cross‐coupling as a key step. Exposure of the cyclization substrate (+)‐**14** to manganese(III) acetate and copper(II) triflate gave the [5.3.0]‐bicyclic γ‐lactones (+)‐**15** in 74 % yield (Scheme 4). As in the racemic series, the lactones (+)‐**15** were isolated as a 6:1 mixture of inseparable C‐1 diastereomers. Krapcho decarboxylation of the lactones (+)‐**15** allowed separation of the C‐1 diastereomeric lactones (−)‐**16**. The major diastereomer (−)‐**16 a** was readily converted into the methyl‐substituted lactone (−)‐**17** on treatment with lithium bis(trimethylsilyl)amide and methyl iodide. As in the racemic series, methylenation of the lactone gave the corresponding enol ether, which, on heating in xylene, gave the desired [5.3.0]‐bicyclic ketone (+)‐**19** (65 % over two‐steps). Alternatively we found that addition of Celite™ post‐methylenation followed by continued heating in toluene at 150 °C gave a one‐pot synthesis of the [5.3.0]‐bicyclic ketone (+)‐**19** in 74 % yield (see Scheme 4).

**Scheme 3:**
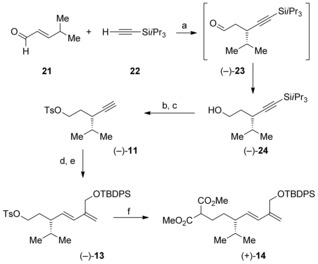
Catalytic enantioselective synthesis of cyclization substrate (+)‐1**4**. a) 2.5 mol % [{Rh(OAc)(C_2_H_4_)_2_}_2_], 6.0 mol % (*S*)‐DTBM‐segphos, CH_3_OH, 40 °C, 24 h, then NaBH_4_, RT, 30 min., 78 %, 99 % *ee*; b) Bu_4_NF, THF, 0 °C–RT, 67 %; c) *p‐*CH_3_C_6_H_4_SO_2_Cl, pyridine, −20 °C–RT, 87 %; d) catecholborane, THF, 70 °C, 18 h; e) **12**, 5 mol % Pd(OAc)_2_, 20 mol % PPh_3_, 2 m LiOH, THF, 40 °C, 4 h, 87 %; f) CH_2_(CO_2_CH_3_)_2_, NaH, THF, 80 °C, 1.5 h, 84 %. DTBM‐segphos=5,5′‐bis[di(3,5‐di‐*tert*‐butyl‐4‐methoxyphenyl)phosphino]‐4,4′‐bi‐1,3‐benzodioxole.

**Scheme 4:**
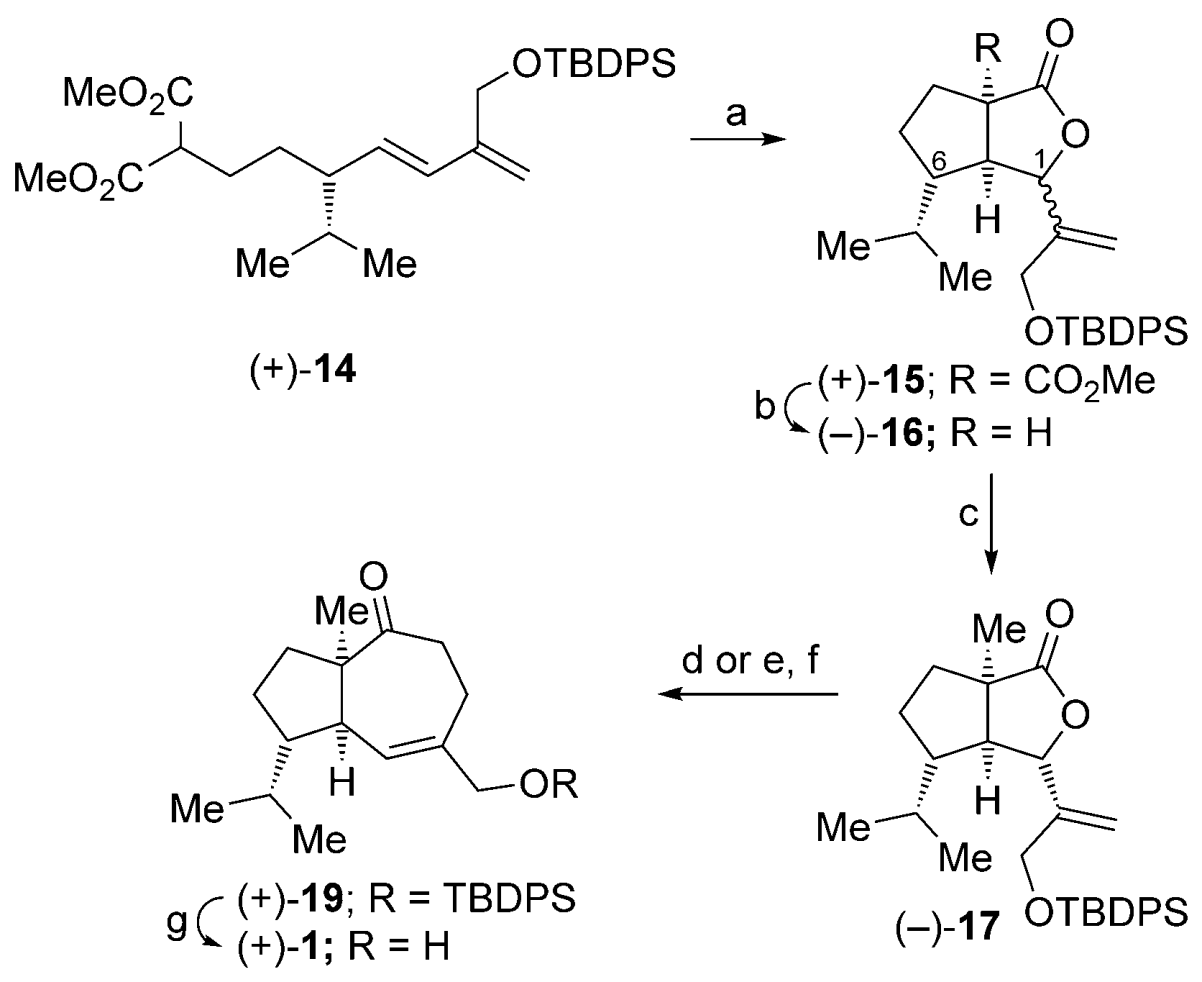
Completion of the synthesis of (+)‐aphanamol. a) Manganese(III) acetate, copper(II) triflate, acetonitrile, reflux, 74 %; b) LiCl, DMF, water, 150 °C, 75 % major diastereomer, 12 % minor diastereomer; c) CH_3_I, ((CH_3_)_3_Si)_2_NLi, THF, −78 °C, 90 %; d) Cp_2_TiMe_2_, toluene, 110 °C, then add Celite™, 150 °C, 74 %; e) Cp_2_Ti(CH_3_)_2_, toluene, reflux, 84 %; f) xylene, reflux, 77 %; g) Bu_4_NF, CH_3_CO_2_H, THF, 0 °C–RT, quant.

Deprotection of the silyl ether with buffered tetra‐*n*‐butylammonium fluoride gave (+)‐aphanamol I (+)‐**1** in quantitative yield. The ^1^H and ^13^C NMR data of our synthetic material were in excellent agreement with that of the natural product and previously reported data on synthetic samples.^1^, ^6^, ^8^, ^37^

In summary, we have developed a synthesis of the biologically active natural product aphanamol I in both racemic and enantiopure forms using a catalytic enantioselective conjugate addition of a silylated alkyne to an α,β‐unsaturated aldehyde, an oxidative radical cyclization, and a ring‐expanding Claisen rearrangement as key steps. Further applications of oxidative radical cyclizations for the synthesis of complex natural products will be reported in due course.

## Supporting information

As a service to our authors and readers, this journal provides supporting information supplied by the authors. Such materials are peer reviewed and may be re‐organized for online delivery, but are not copy‐edited or typeset. Technical support issues arising from supporting information (other than missing files) should be addressed to the authors.

Supplementary
