# Supplementary material for: A Short Synthesis of Aphanamol I in Both Racemic and Enantiopure Forms
Source: Chemistry. 2016 Jul 8;22(33):11597–600. doi: 10.1002/chem.201602669 (PMC5096262; doi:10.1002/chem.201602669)
Supplement: Supplementary file 1 — Supplementary [file CHEM-22-11597-s001.pdf]

CHEMISTRY
A European Journal

Supporting Information

A Short Synthesis of Aphanamol I in both Racemic and Enantiopure Forms

Steven J. Ferrara and Jonathan W. Burton*[a]

chem_201602669_sm_miscellaneous_information.pdf

A Short Synthesis of Aphanamol I in both Racemic and Enantiopure Forms

Steven J. Ferrara[a] and Jonathan W. Burton*[a]

Table of Contents

1.1 Introduction 2

1.2 Experimental Procedures 4

1.3 NMR Spectra and HPLC Traces 33

1.4 ^{13}C NMR comparison of natural and synthetic aphanamol I 56

1.5 References 56

1.1 Introduction

^{1}H and ^{13}C NMR spectra were recorded at 25 °C on a Bruker AV-500 (500/125 MHz), Bruker AV-400 (400/100 MHz) or Bruker DPX-400 (400 MHz) spectrometer. Proton (δ_H) and carbon (δ_C) chemical shifts are quoted in ppm and are internally referenced to the residual protonated solvent signal. Assignments were made on the basis of chemical shifts, coupling constants, COSY, DEPT, HSQC data and comparison with spectra of related compounds. Resonances are described as s (singlet), d (doublet), t (triplet), q (quartet), sept (septet), m (multiplet), dd (double doublet) and so on. Coupling constants (*J*) are given in Hz and are rounded to the nearest 0.1 Hz. H and H' refer to diastereotopic protons attached to the same carbon and imply no particular stereochemistry.

High resolution mass spectra were recorded by the Mass Spectrometry Service, at the University of Oxford Chemical Research Laboratory. *m/z* values are reported in Daltons, with their percentage abundances and the relevant fragment ions in parentheses. High resolution values are calculated to four decimal places from the molecular formula, with all found values reported within a tolerance of 5 ppm. **Low resolution mass spectra** were recorded on a Fisons Platform spectrometer (ES).

Infrared spectra were recorded using a Bruker Tensor 27 Fourier Transform spectrophotometer using thin films on NaCl plates using diamond ATR. Absorption maxima (ν_{max}) are classified as strong (s), medium (m), weak (w) and broad (br) and are quoted in wavenumbers (cm^{-1}).

Optical rotations were measured using a Perkin-Elmer 241 polarimeter in a cell of 1 dm path length.

Melting points were determined using a Leica Galen III Compound Microscope and are uncorrected.

Analytical TLC was performed on Merck DC-Alufolien 60 F_{254} 0.2 mm precoated plates and visualised using acidic vanillin or basic potassium permanganate dips. Retention factors (R_f) are reported with the solvent system used in parentheses. **Flash column chromatography** was performed on Merck 60 silica (particle size 40–63 μm, pore diameter 60 Å) and the solvent system used is recorded in parentheses.

HPLC was carried out on a Agilent 1200 Series fitted with either a Zorbax Rx-SIL column measuring 9.4 × 250 mm (packed with 5 μm beads), a Chiralcel OD column measuring 4.6 × 250 mm (packed with 10 μm beads) or a Chiralpak AD-H column measuring 4.6 × 250 mm (packed with 5 μm beads) with flow rate, solvents and retention times recorded in parentheses.

All non-aqueous reactions were carried out in oven-dried glassware sealed with rubber septa under a positive pressure of dry nitrogen or argon from a manifold or balloon during the course of the reaction. Reactions were stirred using Teflon-coated magnetic stir bars. Elevated temperature reactions were maintained using a Thermowatch-controlled DrySyn heating block. Reagents were purchased from commercial suppliers and used without further purification, unless otherwise stated. Dry solvents were purified using standard techniques. Water used experimentally was deionised. Organic solutions were concentrated using a rotary evaporator. Brine refers to a saturated solution of sodium chloride in water. 'Petrol' refers to the fraction of light petroleum ether boiling in the range of 30-40 or 40-60 °C as stated.

Compound names are as generated by ChemBioDraw Ultra 12.0, with NMR assignments based on either the proton/carbon environments or numbering as per compound name. Compounds titled in italics indicate it is a novel compound.

All intermediates *en route* to racemic aphanamol I were fully characterised. Full data were not obtained for those intermediates *en route* to enantiopure aphanamol I that had been prepared and fully characterised in their racemic form.

1.2 Experimental Procedures

Dimethyl 2-(2-methylpropylidene)malonate 6 according to the procedure of Cardillo[1]

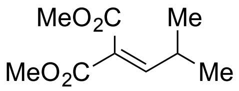

To a stirred solution of freshly distilled isobutyraldehyde (2.16 g, 30.0 mmol, 1.0 eq.) in anhydrous DMSO (10 mL) containing 3 Å molecular sieves was added L-proline (569 mg, 4.9 mmol, 0.16 eq.) and the solution was stirred for 5 min. To this mixture was rapidly added dimethyl malonate (4.36 g, 33.0 mmol, 1.1 eq.), and the resulting mixture was stirred at room temperature for 14 h. The precipitate and molecular sieves were removed by filtration. Water (50 mL) was added and resulting liquid was extracted with EtOAc (3 × 50 mL). The combined organic extracts were sequentially washed with water (150 mL), brine (150 mL), dried ($MgSO_4$), filtered and concentrated under reduced pressure. The residue was purified by flash column chromatography (20% EtOAc/petrol 40-60) to afford dimethyl 2-(2-methylpropylidene)malonate **6** as a colourless oil (4.97 g, 26.7 mmol, 89%).

R_f = 0.28 (20% EtOAc/petrol 40-60)

δ_H (400 MHz, $CDCl_3$) 6.81 (1H, d, *J* = 10.6 Hz, C=C**H**CH), 3.81 (3H, s, $OC\mathbf{H_3}$), 3.77 (3H, s, $OC\mathbf{H_3}$), 2.67 (1H, dsept, *J* = 10.6, 6.6 Hz, CHC**H**$(CH_3)_2$), 1.06 (6H, d, *J* = 6.6 Hz, $CH(C\mathbf{H_3})_2$)

δ_C (100 MHz, $CDCl_3$) 166.0 (C=O), 164.5 (C=O), 155.8 (C=**C**HCH), 125.8 ($(MeO_2C)_2$**C**=CH), 52.3 (O**C**H_3), 52.2 (O**C**H_3), 29.5 (CH**C**H$(CH_3)_2$), 21.8 (CH(**C**$H_3)_2$)

LRMS *m/z* (ESI^+) 209 ($[M+Na]^+$, 100%), 395 ($[2M+Na]^+$, 11%)

HRMS *m/z* (ESI^+) found 209.0784, $C_9H_{14}NaO_4$ requires 209.0784

ν_{max} (film)/cm^{-1} 2962 (m, CH), 2784 (w), 1731 (s, C=O), 1645 (m, C=C), 1438 (m), 1367 (m), 1325 (m), 1258 (s), 1224 (s), 1150 (m), 1058 (m).

Data are consistent with literature values.[1]

***(±)-Dimethyl 2-(4-methyl-1-(trimethylsilyl)pent-1-yn-3-yl)malonate (±)-8 according to the procedure of Ono and Tanaka*[2]**

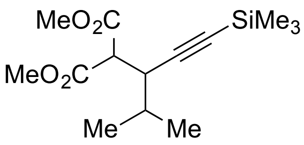

To a vigorously stirred solution of ethynyltrimethylsilane **7** (0.92 g, 10.0 mmol, 1.0 eq.) in anhydrous THF (12.5 mL) at room temperature was added a 1 M solution of ethylmagnesium bromide in THF (10.0 mL, 10.0 mmol, 1.0 eq.). The resulting mixture was stirred for 2 h and then cooled to 0 °C in an ice/water bath. Copper(I) chloride (10.0 mg, 0.10 mmol, 1.0 mol%) was added followed by rapid addition of a solution of dimethyl 2-(2-methylpropylidene)malonate **6** (1.86 g, 10.0 mmol, 1.0 eq.) in THF (12.5 mL) *via* cannula. The mixture was warmed to room temperature and stirred for 30 min. The reaction was quenched with 1 M HCl (30.0 ml) and extracted with EtOAc (3 × 50 mL). The combined organic extracts were sequentially washed with sat. aq. $NaHCO_3$ solution (100 mL), brine (100 mL), dried ($MgSO_4$), filtered and concentrated under reduced pressure. The residue was purified by flash column chromatography (20% Et_2O/petrol 30-40) to afford (±)-dimethyl 2-(4-methyl-1-(trimethylsilyl)pent-1-yn-3-yl)malonate (±)-**8** as a colourless oil (2.68 g, 9.43 mmol, 94%).

R_f = 0.25 (20% Et_2O/petrol 40-60)

δ_H (400 MHz, $CDCl_3$) 3.75 (3H, s, OC**H₃**), 3.73 (3H, s, OC**H₃**), 3.54 (1H, d, *J* = 10.9 Hz, $(MeO_2C)_2$C**H**CH), 3.18 (1H, dd, *J* = 10.9, 3.7, CHC**H**(CH)C≡), 1.73 (1H, septd, *J* = 6.7, 3.7 Hz, CHC**H**$(CH_3)_2$), 1.03 (3H, d, *J* = 6.7 Hz, CH(C**H₃**)₂), 0.93 (3H, d, *J* = 6.7 Hz, CH(C**H₃**)₂), 0.13 (9H, s, Si(C**H₃**)₃)

δ_C (100 MHz, $CDCl_3$) 168.1 (C=O), 167.9 (C=O), 103.2 (C≡**C**TMS), 89.1 (CHC≡C), 54.9 ($(MeO_2C)_2$**C**HCH), 52.6 (O**C**H_3), 52.5 (O**C**H_3), 39.6 (CH**C**H(CH)C≡), 28.7 (CH**C**H$(CH_3)_2$), 21.6 (CH(**C**H_3)$_2$), 16.7 (CH(**C**H_3)$_2$), -0.01 (3C, Si(**C**H_3)$_3$)

LRMS *m/z* (ESI^+) 285 ($[M+H]^+$, 45%), 307 ($[M+Na]^+$, 100), 591 ($[2M+Na]^+$, 72%)

HRMS *m/z* (ESI^+) found 307.1334, $C_{14}H_{24}NaO_4Si$ requires 307.1336

ν_{mmax} (film)/cm^{-1} 2962 (m, CH), 2878 (w), 2846 (w), 2174 (m, C≡C), 1744 (s, C=O), 1437 (m), 1321 (m), 1253 (m), 1159 (m), 1009 (m), 848 (s, Si(CH_3)$_3$).

(±)-Methyl 3-isopropyl-5-(trimethylsilyl)pent-4-ynoate (±)-9

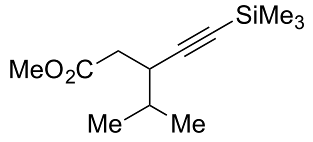

To a stirred solution of (±)-2-(4-methyl-1-(trimethylsilyl)pent-1-yn-3-yl)malonate (±)-**8** (2.84 g, 10.0 mmol, 1.0 eq.) in anhydrous DMF (20.0 mL) was added water (360 mg, 20 mmol, 2.0 eq.) followed by lithium chloride (845 mg, 20.0 mmol, 2.0 eq.). The resultant mixture was heated under reflux (150° C) for 1.5 h and then cooled to room temperature. Water (30 mL) was added to the mixture and extracted with EtOAc (3 × 30 mL). The combined organic extracts were washed sequentially with water (100 mL), brine (100 ml), dried ($MgSO_4$), filtered and concentrated under reduced pressure. The residue was purified by flash column chromatography (6% Et_2O/petrol 30-40) to afford (±)-methyl 3-isopropyl-5-(trimethylsilyl)pent-4-ynoate (±)-**8** as a colourless oil (1.84 g, 8.14 mmol, 81%).

R_f = 0.34 (10% Et_2O/petrol 40-60)

δ_H (400 MHz, $CDCl_3$) 3.69 (3H, s, OC**H$_3$**), 2.82 (1H, ddd, *J* = 8.1, 7.1, 4.8 Hz, CH$_2$C**H**(CH)C≡), 2.50 (1H, dd, *J* = 15.2, 8.1 Hz, OC(=O)C**H$_2$**CH), 2.44 (1H, dd, *J* = 15.2, 7.1 Hz, OC(=O)C**H$_2$**CH), 1.72 (1H, septd, *J* = 6.7, 4.8 Hz, CHC**H**(CH$_3$)$_2$), 0.99 (3H, d, *J* = 6.7 Hz, CH(C**H$_3$**)$_2$), 0.95 (3H, d, *J* = 6.7 Hz, CH(C**H$_3$**)$_2$), 0.13 (9H, s, Si(C**H$_3$**)$_3$)

δ_C (100 MHz, $CDCl_3$) 172.3 (C=O), 106.7 (C≡**C**TMS), 87.0 (CH**C**≡C), 51.6 (O**C**H$_3$), 37.8 (OC(=O)**C**H$_2$CH), 35.9 (CH$_2$**C**H(CH)C≡), 30.9 (CH**C**H(CH$_3$)$_2$), 20.9 (CH(**C**H$_3$)$_2$), 17.8 (CH(**C**H$_3$)$_2$), 0.10 (3C, Si(**C**H$_3$)$_3$)

LRMS *m/z* (ESI$^+$) 227 ([M+H]$^+$, 23%), 249 ([M+Na]$^+$, 100%)

HRMS *m/z* (ESI$^+$) found 249.1283, $C_{12}H_{22}NaO_2Si$ requires 249.1281

ν_{max} (film)/cm^{-1} 2962 (m, CH), 2898 (w), 2877 (w), 2171 (m, C≡C), 1744 (s, C=O), 1437 (m), 1359 (m), 1253 (m, CO-O), 1164 (m, C-O-C), 1017 (m), 896 (m), 845 (s, Si(CH_3)$_3$), 760 (m).

(±)-3-Isopropyl-5-(trimethylsilyl)pent-4-yn-1-ol (±)-10

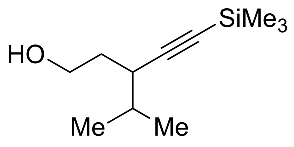

To a stirred suspension of $LiAlH_4$ (281 mg, 7.40 mmol, 1.0 eq.) in anhydrous Et_2O (7.40 mL) cooled to 0 °C in an ice/water bath, was added dropwise a solution of methyl (±)-3-isopropyl-5-(trimethylsilyl)pent-4-ynoate (±)-**9** (1.68 g, 7.40 mmol, 1.0 eq.) in anhydrous Et_2O (7.40 mL). The resulting mixture was stirred at 0 °C for 30 min. Water (0.28 mL) was carefully added dropwise, followed by addition of a 10% NaOH solution (0.28 mL) and water (0.56 mL). The mixture was stirred for 5 min, dried (Na_2SO_4), filtered and concentrated under reduced pressure. The residue was purified by flash column chromatography (25% EtOAc/petrol 40–60) to afford (±)-3-isopropyl-5-(trimethylsilyl)pent-4-yn-1-ol (±)-**10** as a colourless oil (1.47 g, 6.90 mmol, 93%).

R_f = 0.31 (30% EtOAc/petrol 40–60)

δ_H (400 MHz, $CDCl_3$) 3.81 (2H, m, HOC**H**$_2$CH$_2$), 2.41 (1H, td, *J* = 9.6, 5.5 Hz, CH$_2$C**H**(CH)C≡), 1.93 (1H, s, **H**OCH$_2$), 1.75–1.61 (3H, m, OCH$_2$C**H**$_2$CH and CHC**H**(CH$_3$)$_2$), 0.99 (3H, d, *J* = 6.8 Hz, CH(C**H**$_3$)$_2$), 0.96 (3H, d, *J* = 6.8 Hz, CH(C**H**$_3$)$_2$), 0.15 (9H, s, Si(C**H**$_3$)$_3$)

δ_C (100 MHz, $CDCl_3$) 108.6 (C≡**C**TMS), 87.4 (CH**C**≡C), 61.7 (HOC**H**$_2$CH$_2$), 36.6 (CH$_2$**C**H(CH)C≡), 35.2 (OCH$_2$**C**H$_2$CH), 31.7 (CH**C**H(CH$_3$)$_2$), 20.8 (CH(**C**H$_3$)$_2$), 18.5 (CH(**C**H$_3$)$_2$), 0.14 (3C, Si(**C**H$_3$)$_3$)

LRMS *m/z* (ESI$^+$) 199 ([M+H]$^+$, 19%), 221 ([M+Na]$^+$, 100%)

HRMS *m/z* (ESI$^+$) found 221.1333, $C_{11}H_{22}NaOSi$ requires 221.1332

ν_{max} (film)/cm^{-1} 3326 (br, OH), 2961 (s, CH), 2880 (m), 2361 (w), 2166 (m, C≡C), 1773 (w), 1684 (br), 1466 (w), 1400 (w), 1251 (m), 845 (s, Si(CH$_3$)$_3$), 758 (m).

3-Isopropylpent-4-yn-1-ol

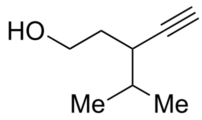

Racemic: To a stirred solution of (±)-3-isopropyl-5-(trimethylsilyl)pent-4-yn-1-ol (±)-**10** (5.74 g, 28.9 mmol, 1.0 eq.) in anhydrous THF (72.0 mL) cooled to 0 °C in an ice/water bath was added dropwise a 1 M solution of TBAF in THF (34.7 mL, 34.7 mmol, 1.2 eq.). After complete addition the mixture was warmed to room temperature and stirred for 1.5 h. Sat. aq. NH_4Cl (100 mL) was added to the mixture and extracted with Et_2O (3 × 100 mL). The combined organic extracts were successively washed with 1 M HCl (300 mL), water (300 ml), brine (300 ml), dried ($MgSO_4$), filtered and concentrated under reduced pressure. The residue was purified by flash column chromatography (50% Et_2O/petrol 40-60) to afford (±)-3-isopropylpent-4-yn-1-ol as a colourless oil (3.34 g, 26.6 mmol, 92%).

R_f = 0.24 (50% Et_2O/petrol 40-60)

δ_H (400 MHz, $CDCl_3$) 3.87–3.75 (2H, m, HOC**H₂**CH₂), 2.43 (1H, dtd, *J* = 7.5, 5.0, 2.5 Hz, CH₂C**H**(CH)C≡CH), 2.08 (1H, d, *J* = 2.5 Hz, CHC≡C**H**), 1.91 (1H, t, *J* = 5.4 Hz, **H**OCH₂CH₂), 1.78–1.61 (3H, m, CH₂C**H₂**CH and CHC**H**(CH₃)₂), 1.00 (3H, d, *J* = 6.7 Hz, CH(C**H₃**)₂), 0.97 (3H, d, *J* = 6.7 Hz, CH(C**H₃**)₂)

δ_C (100 MHz, $CDCl_3$) 85.6 (CH**C**≡CH), 70.8 (CHC≡**C**H), 61.3 (HO**C**H₂CH₂), 35.2 (CH₂**C**H₂CH), 35.1 (CH₂**C**H(CH)C≡CH), 31.5 (CH**C**H(CH₃)₂), 20.8 (CH(**C**H₃)₂), 18.3 (CH(**C**H₃)₂)

HRMS *m/z* (FI^+) found 127.1117, $C_8H_{15}O$ requires 127.1123

ν_{max} (film)/cm^{-1} 3303 (br, OH), 2961 (s, CH), 2876 (m), 2110 (w, C≡C), 1466 (m), 1387 (w), 1370 (w), 1045 (s), 629 (s)

Data are consistent with literature value.[3]

Asymmetric: To a stirred solution of (*S*)-3-isopropyl-5-(triisopropylsilyl)pent-4-yn-1-ol (–)-**24** (1.59 g, 5.65 mmol, 1.0 eq.) in anhydrous THF (22.6 mL) cooled to 0 °C in an ice/water bath was added dropwise a 1 M solution of TBAF in THF (11.3 mL, 11.3 mmol, 2.0 eq.). After complete addition the mixture was

warmed to room temperature and stirred for 16 h. 1 M HCl (40 mL) was added to the mixture and extracted with Et_2O (3 × 20 mL). The combined organic extracts were sequentially washed with sat. aq. NH_4Cl solution (100 mL), brine (100 ml), dried (Na_2SO_4), filtered and concentrated under reduced pressure. The residue was purified by flash column chromatography (50% Et_2O/petrol 30-40) to afford (*S*)-3-isopropylpent-4-yn-1-ol as a colourless oil (480 mg, 3.80mmol, 67%).

$[\alpha]_D^{20}$ -23.7 (*c* 1.02, $CHCl_3$) for >99% ee

3-Isopropylpent-4-yn-1-yl 4-methylbenzenesulfonate 11

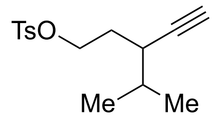

Racemic: To a stirred solution of (±)-3-isopropylpent-4-yn-1-ol (631 mg, 5.00 mmol, 1.0 eq.) in anhydrous DCM (5.0 mL) cooled to 0 °C in an ice/water bath was added pyridine (791 mg, 10.0 mmol, 2.0 eq.) followed by portionwise addition of *p*-toluenesulfonyl chloride (1.43 g, 7.50 mmol, 1.5 eq.). After complete addition, the mixture was warmed to room temperature and stirred for 6 h. 1 M HCl (20 mL) was added and the mixture was stirred for 5 min. The organic layer was separated and the aqueous layer was further extracted with DCM (2 × 20 mL). The combined organic layers were washed sequentially with sat. aq. $NaHCO_3$ solution (50 mL), dried ($MgSO_4$), filtered and concentrated under reduced pressure. The residue was purified by flash column chromatography (30% Et_2O/petrol 30–40) to afford (±)3-isopropylpent-4-yn-1-yl 4-methylbenzenesulfonate (±)-**11** as a colourless oil (1.22 g, 4.35 mmol, 87%).

R_f = 0.23 (10% EtOAc/petrol 40-60)

δ_H (400 MHz, $CDCl_3$) 7.80 (2H, d, *J* = 8.4 Hz, Ar**H**), 7.35 (2H, d, *J* = 8.4 Hz, Ar**H**), 4.27–4.14 (2H, m, TsOC**H$_2$**CH$_2$), 2.45 (3H, s, ArC**H$_3$**), 2.36 (1H, dtd, *J* = 7.1, 4.6, 2.4 Hz, CH$_2$C**H**(CH)C≡CH), 1.97 (1H, d, *J* = 2.4 Hz, CHC≡C**H**), 1.86–1.78 (1H, m, OCH$_2$C**H$_2$**CH), 1.73–1.59 (2H, m, OCH$_2$C**H$_2$**CH and CHC**H**(CH$_3$)$_2$), 0.95 (3H, d, *J* = 6.7 Hz, CH(C**H$_3$**)$_2$), 0.92 (3H, d, *J* = 6.7 Hz, CH(C**H$_3$**)$_2$)

δ_C (100 MHz, $CDCl_3$) 144.7 (Ar), 133.0 (Ar), 129.1 (2C, Ar), 127.9 (2C, Ar), 83.9 (CH**C**≡CH), 71.4 (CHC≡**C**H), 68.9 (TsO**C**H_2CH_2), 34.6 (CH_2**C**H(CH)C≡CH), 31.8 (OCH_2**C**H_2CH), 31.2 (CH**C**H(CH_3)$_2$), 21.6 (Ar**C**H_3), 20.7 (CH(**C**H_3)$_2$), 18.1 (CH(**C**H_3)$_2$)

LRMS *m/z* (ESI^+) 281 ($[M+H]^+$, 20%), 303 ($[M+Na]^+$, 95%), 583 ($[2M+Na]^+$, 100%)

HRMS *m/z* (ESI^+) found 303.1015, $C_{15}H_{20}NaO_3S$ requires 303.1025

ν_{max} (film)/cm^{-1} 3290 (m, ≡C-H), 2964 (m, CH), 2883 (m), 2112 (C≡C), 1599 (m), 1467 (m), 1357 (s, R-SO_2-R), 1177 (s, R-SO_2-R), 1095 (m), 984 (s), 909 (s), 814 (m), 777 (m), 660 (br, ≡C-H).

Asymmetric: To a stirred solution of (*S*)-3-isopropylpent-4-yn-1-ol (480 mg, 3.80 mml, 1.0 eq.) in pyridine (3.80 mL) cooled to -20 °C in a dry ice/acetone bath was added *p*-toluenesulfonyl chloride (1.08 g, 5.70 mmol, 1.5 eq.) portionwise. The resulting mixture was slowly warmed to room temperature and stirred for 18 h. 1 M HCl (20 mL) and extracted with EtOAc (3 × 10 mL). The combined organic extracts were sequentially washed water (50 mL), brine (50 mL), dried ($MgSO_4$), filtered and concentrated under reduced pressure. The residue was purified by flash column chromatography (10% EtOAc/petrol 40-60) to afford (*S*)-3-isopropylpent-4-yn-1-yl 4-methylbenzenesulfonate (–)-**11** as a colourless oil (927 mg, 3.31 mmol, 87%, >99% ee (*S*)).

$[\alpha]_D^{20}$ -50.9 (*c* 1.01, $CHCl_3$) for >99% ee.

3-Isopropyl-5-(triisopropylsilyl)pent-4-yn-1-ol 24

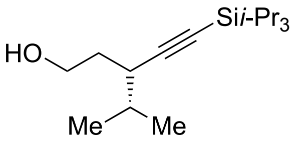

Asymmetric: To a vigorously stirred solution of chlorobis(ethylene)rhodium(I) dimer (70.7 mg, 0.18 mmol, 2.5 mol%) in Ar sparged Et_2O (18.2 mL) cooled to -30 °C was added in one portion sodium acetate trihydrate (150 mg, 1.10 mmol, 0.15 eq.). The resulting yellow mixture was slowly warmed to room temperature over 1 h and then filtered *via* a cannula fitted with a glass filter paper. The filtrate was concentrated under reduced pressure to afford an orange/red solid. The residue was re-suspended in Ar

sparged anhydrous MeOH (14.6 mL), after which (S)-(+)-DTBM-SEGPHOS (517 mg, 0.44 mmol, 6.0 mol%) was added and the resulting solution was stirred at room temperature for 15 min. 4-Methyl-2-pentenal **21** (716 mg, 7.30 mmol, 1.0 eq.) and (triisopropylsilyl)acetylene **22** (2.66 g, 14.6 mmol, 2.0 eq.) were added and the mixture was heated at 40°C for 24 h, then cooled to room temperature. Sodium borohydride (11.0 g, 29.2 mmol, 4.0 eq.) was then carefully added portionwise and the mixture was stirred for a further 30 min. Water (20 mL) was added and the mixture was extracted with DCM (3 × 20 mL). The combined organic extracts were sequentially washed with water (30 ml), dried (Na_2SO_4), filtered and concentrated under reduced pressure. The residue was purified by flash column chromatography (5-20% gradient EtOAc/petrol 40-60) to afford (*S*)-3-isopropyl-5-(triisopropylsilyl)pent-4-yn-1-ol (–)-**24** as a yellow oil (1.60 g, 5.69 mmol, 78%, >99% *ee* (*S*)). The *ee* was determined by chiral HPLC analysis of the corresponding benzoyl ester.

$[\alpha]_D^{20}$ -8.5 (*c* 0.97, $CHCl_3$) for >99% *ee*

R_f = 0.30 (20% EtOAc/petrol 40-60)

δ_H (500 MHz, $CDCl_3$) 3.85 (2H, td, *J* = 6.3, 2.8 Hz, HOC**H₂**CH₂), 2.51–2.45 (1H, m, CH₂C**H**(CH)C≡), 1.75–1.67 (4H, m, **H**OCH₂, OCH₂C**H₂**CH & CHC**H**(CH₃)₂), 1.09-1.05 (3H, n, Si(C**H**(*i*-Pr)₃)), 1.07 (18H, s, Si(CH(C**H₃**)₂)₃), 1.02 (3H, d, *J* = 6.7 Hz, CH(C**H₃**)₂), 0.99 (3H, d, *J* = 6.7 Hz, CH(C**H₃**)₂)

δ_C (125 MHz, $CDCl_3$) 110.1 (C≡**C**TIPS), 83.2 (CH**C**≡C), 61.9 (HOC**H₂**CH₂), 36.7 (CH₂**C**H(CH)C≡), 35.6 (OCH₂**C**H₂CH), 31.9 (CH**C**H(CH₃)₂), 21.1 (CH(**C**H₃)₂), 18.6 (6C, Si(CH(**C**H₃)₂)₃), 18.2 (CH(**C**H₃)₂), 11.3 (3C, Si(**C**H(CH₃)₂)₃)

LRMS *m/z* (ESI^+) 283 ($[M+H]^+$, 18%), 305 ($[M+Na]^+$, 100%)

HRMS *m/z* (ESI^+) found 305.2271, $C_{17}H_{34}NaOSi$ requires 305.2271

ν_{max} (film)/cm^{-1} 3323 (br, OH), 2958 (s), 2942 (s), 1891 (m), 2865 (s), 2164 (m, C≡C), 1463 (m), 1385 (w), 1367 (w), 1044 (m), 1016 (m), 996 (m), 882 (s), 665 (s).

Racemic: To a stirred suspension of $LiAlH_4$ (251 mg, 6.60 mmol, 1.0 eq.) in anhydrous Et_2O (11.0 mL) cooled to 0 °C in an ice/water bath, was added dropwise a solution of (±)-methyl 3-isopropyl-5-(triisopropylsilyl)pent-4-ynoate (2.05 g, 6.60 mmol, 1.0 eq. prepared analogously to (±)-**9**) in anhydrous Et_2O

(11.0 mL). The mixture was warmed to room temperature and stirred for 2 h. Water (0.25 mL) was carefully added dropwise to the mixture followed by 10% aq. NaOH solution (0.25 mL) and stirred for 5 min. Additional water (0.50 mL) was added, after which the mixture was dried ($MgSO_4$), filtered and concentrated under reduced pressure. The residue was purified by flash column chromatography (20% EtOAc/petrol 40-60) to afford 3-isopropyl-5-(triisopropylsilyl)pent-4-yn-1-ol (±)-**24** as a colourless oil (1.75 g, 5.92 mmol, 94%).

3-Isopropyl-5-(triisopropylsilyl)pent-4-yn-1-yl benzoate

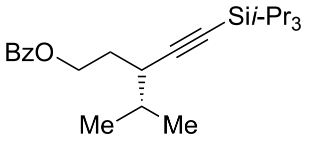

Asymmetric. To a stirred solution of (*S*)-3-isopropyl-5-(triisopropylsilyl)pent-4-yn-1-ol (–)-**24** (56.4 mg, 0.20 mmol, 1.0 eq.) in anhydrous DCM (10.0 mL) at room temperature, was added DMAP (224 mg, 2.00 mmol, 10.0 eq.) and benzoic anhydride (294 mg, 1.30 mmol, 6.5 eq.). The resulting mixture was stirred at room temperature for 24 h. Sat. aq. NH_4Cl solution (10 mL) was added to the mixture and the organic layer was separated. The aq. layer was further extracted with DCM (2 × 10 mL). The combined organic extracts were dried ($MgSO_4$), filtered and concentrated under reduced pressure. The residue was purified by flash column chromatography (5% EtOAc/petrol 40-60) to afford (*S*)-3-isopropyl-5-(triisopropylsilyl)pent-4-yn-1-yl benzoate as a colourless oil (76.0 mg, 0.19 mmol, 98%, >99% ee (*S*)). The ee was measured by HPLC (Chiralcel OD column, flow 0.6 mL/min, 0.2% IPA/hexane, 230 nm, t_1 = 18.9 min (*S*), t_2 = 20.5 min (*R*)

$[\alpha]_D^{20}$ -66.0 (*c* 0.96, $CHCl_3$) for >99% ee, [lit. $[\alpha]_D^{20}$ +51 (*c* 0.98, $CHCl_3$) for (*R*)-enantiomer][4]

R_f = 0.31 (5% EtOAc/petrol 40-60)

δ_H (500 MHz, $CDCl_3$) 8.09–8.04 (2H, m, Ar**H**), 7.59–7.54 (1H, m, Ar**H**), 7.48–7.42 (2H, m, Ar**H**), 4.57 (1H, ddd, *J* = 11.1, 6.8, 4.9 Hz, OC**H$_2$**CH$_2$), 4.47 (1H, ddd, *J* = 11.1, 8.3, 6.4 Hz, OC**H$_2$**CH$_2$), 2.56 (1H, dt, *J* = 10.0, 4.8 Hz, CH$_2$C**H**(CH)C≡), 2.00–1.82 (2H, m, CH$_2$C**H$_2$**CH), 1.77 (1H, septd, *J* = 6.7, 4.8 Hz, CHC**H**(CH$_3$)$_2$), 1.09-1.04 (3H, m, Si(C**H**(*i*-Pr)$_3$)), 1.08 (18H, s, Si(CH(C**H$_3$**)$_2$)$_3$), 1.06 (3H, d, *J* = 6.7 Hz, CH(C**H$_3$**)$_2$), 1.03 (3H, d, *J* = 6.7 Hz, CH(C**H$_3$**)$_2$)

δ_C (125 MHz, $CDCl_3$) 166.5 (**C**=O), 132.8 (Ar), 130.4 (Ar), 129.6 (2C, Ar), 128.3 (2C, Ar), 109.0 (C≡**C**TIPS), 83.1 (CH**C**≡C), 63.6 (OC**H**$_2$CH$_2$), 36.5 (CH$_2$**C**H(CH)C≡), 32.0 (OCH$_2$**C**H$_2$CH), 31.6 (CH**C**H(CH$_3$)$_2$), 21.2 (CH(**C**H$_3$)$_2$), 18.6 (6C, Si(CH(**C**H$_3$)$_2$)$_3$), 18.1 (CH(**C**H$_3$)$_2$), 11.3 (3C, Si(**C**H(CH$_3$)$_2$)$_3$)

LRMS *m/z* (ESI^+) 387 ($[M+H]^+$, 17%), 409 ($[M+Na]^+$, 100), 795 ($[2M+Na]^+$, 30)

HRMS *m/z* (ESI^+) found 409.2525, $C_{24}H_{38}NaO_2Si$ requires 409.2533

ν_{max} (film)/cm^{-1} 2959 (s, CH), 2942 (s, CH), 2891 (m, CH), 2865 (s, CH), 2166 (m, C≡C), 1722 (s, C=O), 1463 (m), 1269 (s), 1176 (m), 1114 (s), 1070 (m), 1013 (m), 883 (s).

Racemic

The sample of (±)-3-isopropyl-5-(triisopropylsilyl)pent-4-yn-1-yl benzoate was prepared from (±)-3-isopropyl-5-(triisopropylsilyl)pent-4-yn-1-ol on the same scale and under the same reaction conditions as described above.

2-Iodoprop-2-en-1-ol according to the procedure of Ishii[5]

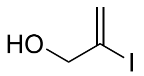

To a stirred a stirred solution of sodium iodide (7.19 g, 48.0 mmol, 1.2 eq.) in acetonitrile (80.0 mL) cooled to 0 °C in an ice/water bath was added trimethylsilyl chloride (5.21 g, 48.0 mmol, 1.2 eq.) and the reaction mixture was stirred for 15 min. To this mixture was then added water (432 mg, 24.0 mmol, 0.6 eq.) followed by rapid dropwise addition of propargyl alcohol (2.24 g, 40.0 mmol, 1.0 eq.). After complete addition, the mixture was warmed to room temperature and stirred for 6 h. Sat. aq. $NaHCO_3$ solution (100 mL) was added to the mixture and extracted with Et_2O (3 × 100 mL). The combined organic extracts were sequentially washed with 1 M NaOH (300 mL), sat. aq. sodium thiosulfate solution (300 mL), dried (Na_2SO_4), filtered and concentrated under reduced pressure. The residue was purified by flash column chromatography (50% Et_2O/petrol 30-40) to afford 2-iodoprop-2-en-1-ol as a colourless oil (3.97 g, 21.6 mmol, 54%).

R_f = 0.30 (50% Et_2O/petrol 40-60)

δ_H (400 MHz, $CDCl_3$) 6.38 (1H, q, *J* = 1.3 Hz, C=C**H$_2$**), 5.85 (1H, q, *J* = 1.3 Hz, =C**H$_2$**), 4.15 (2H, dt, *J* = 6.6, 1.3 Hz, C**H$_2$**OH), 3.03 (1H, t, *J* = 6.6, Hz, CH_2O**H**)

δ_C (100 MHz, $CDCl_3$) 124.5 (C=**C**H_2), 110.4 (**C**=CH_2), 70.9 (**C**H_2OH); HRMS *m/z* (FI^+) found 183.9386, C_3H_5IO requires 183.9385

ν_{max} (film)/cm^{-1}3305 (br, OH), 2910 (m), 2856 (m), 1808 (w, =CH_2 overtone), 1624 (m, C=C), 1443 (m), 1399 (m), 1229 (m), 1143 (m), 1028 (s), 976 (m), 899 (s, C=C-H), 643 (m, C-I).

Data are consistent with literature values.[5]

tert-*Butyl((2-iodoallyl)oxy)diphenylsilane 12*

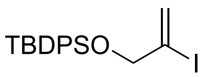

To a stirred a stirred solution of 2-iodoprop-2-en-1-ol (3.40 g, 18.5 mmol, 1.0 eq.) in anhydrous DMF (61.7 mL) cooled to 0 °C in an ice/water bath was added imidazole (3.15 g, 46.3 mmol, 2.5 eq.) followed by dropwise addition *tert*-butyl diphenylchlorosilane (5.59 g, 20.3 mmol, 1.1 eq.). After complete addition, the mixture was warmed to room temperature and stirred for 14 h. Sat. aq. NH_4Cl solution (70 mL) was added to the mixture and extracted with EtOAc (3 × 70 mL). The combined organic extracts were sequentially washed with water (200 mL), brine (200 mL), dried ($MgSO_4$), filtered and concentrated under reduced pressure. The residue was purified by flash column chromatography (0-4% gradient EtOAc/petrol 40-60) to afford *tert*-butyl((2-iodoallyl)oxy)diphenylsilane **12** as a colourless oil (7.56 g, 17.9 mmol, 97%)

R_f = 0.57 (2% EtOAc/petrol 40-60)

δ_H (400 MHz, $CDCl_3$) 7.75–7.72 (4H, m, Ar**H**), 7.52–7.41 (6H, m, Ar**H**), 6.62 (1H, q, *J* = 1.8 Hz, C=C**H$_2$**), 5.92 (1H, q, *J* = 1.8 Hz, C=C**H$_2$**), 4.28 (2H, t, *J* = 1.8 Hz, C**H$_2$**OTBDPS), 1.15 (9H, s, SiC(C**H$_3$**)$_3$)

δ_C (100 MHz, $CDCl_3$) 135.5 (4C, **Ar**), 133.0 (2C, **Ar**), 129.9 (2C, **Ar**), 127.9 (4C, **Ar**), 123.3 (C=**C**H_2), 108.9 (**C**=CH_2), 71.4 (**C**H_2OTBDPS), 26.8 (3C, SiC(**C**H_3)$_3$), 19.4 (Si**C**(CH_3)$_3$)

LRMS *m/z* (ESI^+) 423 ($[M+H]^+$, 50%), 445 ($[M+Na]^+$, 100%); HRMS *m/z* (ESI^+) found 445.0455, $C_{19}H_{23}INaOSi$ requires 445.0455

ν_{max} (film)/cm^{-1} 3071 (w), 2957 (m), 2933 (m), 2890 (m), 2856 (m), 1960 (w, Ph overtone), 1893 (w, Ph overtone), 1823 (w, Ph overtone), 1625 (m, C=C), 1468 (m), 1427 (m), 1393 (m), 1364 (m), 1260 (m), 1188 (m), 1108 (m), 1076 (s, Si-O-C), 1002 (m), 899 (m, C=C-H), 818 (m), 739 (s, Si-C).

(E)-6-(((tert-Butyldiphenylsilyl)oxy)methyl)-3-isopropylhepta-4,6-dien-1-yl 4-methylbenzenesulfonate 13

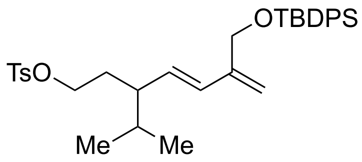

Racemic: To a stirred solution of (±)-3-isopropylpent-4-yn-1-yl 4-methylbenzenesulfonate (±)-**11** (280 mg, 1.00 mmol, 1.1 eq.) in freeze-thaw degassed anhydrous THF (1.00 mL) was added dropwise catecholborane (144 mg, 1.20 mmol, 1.3 eq.). The resulting mixture was heated under reflux (70 °C) for 14 h and cooled to room temperature. The mixture was concentrated under reduced pressure to afford the crude boronate ester as a cloudy oil. Palladium(II) acetate (7.86 mg, 0.03 mmol, 5 mol%) and triphenylphosphine (36.7 mg, 0.12 mmol, 20 mol%) were dissolved in freeze-thaw degassed anhydrous THF (2.50 mL) and stirred at room temperature for 20 min. To the yellow solution was then added dropwise a solution of *tert*-butyl((2-iodoallyl)oxy)diphenylsilane **12** (384 mg, 0.91 mmol, 1.0 eq.) and crude boronate ester in freeze-thaw degassed anhydrous THF (2.50 mL). After complete addition, freeze-thaw degassed aq. 2 M LiOH (2.50 ml) was added to the mixture and stirred at 40 °C for 2 h. Water (10 mL) was added to the mixture and extracted with Et_2O (3 × 10 mL). The combined organic extracts were sequentially washed with sat. aq. $NaHCO_3$ solution (30 mL), dried ($MgSO_4$), filtered and concentrated under reduced pressure. The residue was purified by flash column chromatography (20% Et_2O/petrol 40–60) to afford (±)-(*E*)-6-(((*tert*-butyldiphenylsilyl)oxy)methyl)-3-isopropylhepta-4,6-dien-1-yl 4-methylbenzenesulfonate (±)-**13** as a colourless oil (457 mg, 0.79 mmol, 87%)

R_f = 0.28 (20% Et_2O/petrol 40-60)

δ_H (400 MHz, C_6D_6) 7.90–7.84 (4H, m, Ar**H**), 7.84 (2H, d, J = 8.2 Hz, Ar**H**), 7.35–7.30 (6H, m, Ar**H**), 6.89 (2H,d, J = 8.2 Hz, Ar**H**), 5.92 (1H, d, J = 16.1 Hz, CH=C**H**C(=CH_2)), 5.56 (1H, d, J = 1.6 Hz, C=C$\mathbf{H_2}$), 5.19 (1H, dd, J = 16.1, 9.5 Hz, CH(CH)C**H**=CH), 5.09 (1H, d, J = 1.6 Hz, C=C$\mathbf{H_2}$), 4.49 (2H, s,

C(=CH_2)C**H**$_2$OTBDPS), 4.05–3.98 (1H, m, TsOC**H**$_2$CH$_2$), 3.89 (1H, td, *J* = 9.3, 5.5 Hz, TsOC**H**$_2$CH$_2$), 2.03 (3H, s, ArC**H**$_3$), 1.87–1.77 (1H, m, CH$_2$C**H**(CH)CH=), 1.72–1.63 (1H, m, OCH$_2$C**H**$_2$CH), 1.35–1.24 (1H, m, CHC**H**(CH$_3$)$_2$), 1.24 (9H, s, SiC(C**H**$_3$)$_3$), 1.22–1.07 (1H, m, OCH$_2$C**H**$_2$CH), 0.77 (3H, d, *J* = 6.8, Hz, CH(C**H**$_3$)$_2$), 0.69 (3H, d, *J* = 6.8, Hz, CH(C**H**$_3$)$_2$)

δ_C (100 MHz, C_6D_6) 144.3 (Ar), 144.2 (**C**=CH$_2$), 135.8 (4C, Ar), 134.3 (Ar), 133.8 (Ar), 133.7 (Ar), 132.2 (CH=**C**HC(=CH$_2$)), 130.3 (CH(CH)**C**H=CH), 130.1 (Ar), 130.1 (Ar), 129.9 (2C, Ar), 128.2 (2C, Ar), 128.1 (2C, Ar), 128.1 (2C, Ar), 114.0 (C=**C**H$_2$), 69.0 (TsO**C**H$_2$CH$_2$), 64.1 (C(=CH$_2$)**C**H$_2$OTBDPS), 46.1 (CH$_2$**C**H(CH)CH=), 32.1 (CH**C**H(CH$_3$)$_2$), 31.5 (OCH$_2$**C**H$_2$CH), 26.9 (3C, SiC(**C**H$_3$)$_3$), 21.2 (Ar**C**H$_3$), 20.5 (CH(**C**H$_3$)$_2$), 19.5 (Si**C**(CH$_3$)$_3$), 19.1 (CH(**C**H$_3$)$_2$)

LRMS *m/z* (ESI$^+$) 599 ([M+Na]$^+$ 100%)

HRMS *m/z* (ESI$^+$) found 599.2618, $C_{34}H_{44}NaO_4SSi$ requires 599.2622

ν_{max} (film)/cm^{-1} 3061 (w, =CH$_2$), 2957 (m, CH), 2863 (m, CH), 1901 (w, Ph overtone), 1815 (w, Ph overtone), 1600 (w, C=C), 1464 (m), 1431 (m), 1359 (s, R-SO$_2$-R), 1179 (s, R-SO$_2$-R), 1103 (s, SiO), 959 (m, C=CH$_2$), 909 (m, C=CH$_2$).

Asymmetric: To a stirred solution of (*S*)-3-isopropylpent-4-yn-1-yl 4-methylbenzenesulfonate (–)-**11** (920 mg, 3.28 mmol, 1.1 eq.) in Ar sparged anhydrous THF (3.28 mL) was added dropwise catecholborane (472 mg, 3.93 mmol, 1.3 eq.). The resulting mixture was heated at 70 °C for 18 h and cooled to room temperature. The mixture was concentrated under reduced pressure to afford the crude boronate ester, as a pale yellow cloudy oil. Palladium(II) acetate (33.4 mg, 0.15 mmol, 5 mol%) and triphenylphosphine (156 mg, 0.60 mmol, 20 mol%) were suspended in Ar sparged THF (8.20 mL) and stirred at room temperature for 20 min. To the yellow solution was added dropwise a solution of *tert*-butyl((2-iodoallyl)oxy)diphenylsilane **12** (1.26 g, 2.98 mmol, 1.0 eq.) and crude boronate ester in Ar sparged anhydrous THF (8.20 mL). After complete addition, Ar sparged aq. 2 M LiOH (8.20 ml, 5.5 eq.) was added to the mixture and then heated at 40 °C for 4 h. Water (20 mL) was added to the mixture and extracted with EtOAc (3 × 10 mL). The combined organic extracts were sequentially washed with sat. aq. $NaHCO_3$ solution (50 mL), brine (50 mL), dried (Na_2SO_4), filtered and concentrated under reduced pressure. The residue was purified by flash column

chromatography (10% EtOAc/petrol 40-60 with 1% triethylamine) to afford (*S,E*)-6-(((*tert*-butyldiphenylsilyl)oxy)methyl)-3-isopropylhepta-4,6-dien-1-yl 4-methylbenzenesulfonate (–)-**13** as a pale yellow oil (1.50 g, 2.85 mmol, 87%, >99% ee (*S*)).

$[\alpha]_D^{20}$ -5.0 (*c* 1.02, DCM) for >99% ee

*(*E*)-Dimethyl 2-(6-(((*tert*-butyldiphenylsilyl)oxy)methyl)-3-isopropylhepta-4,6-dien-1-yl)malonate 14*

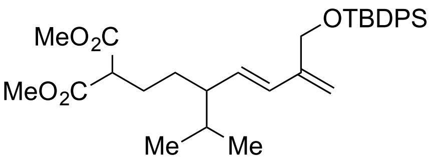

Racemic: To a stirred suspension of hexane washed NaH (67.7 mg, 2.82 mmol, 3.0 eq.), in anhydrous DMF (2.35 mL) cooled to 0 °C in an ice/water bath, was carefully added dropwise dimethyl malonate (373 mg, 2.82 mmol, 3.0 eq.). The resultant mixture was stirred for 20 min at this temperature, after which a solution of (±)-(*E*)-6-(((*tert*-butyldiphenylsilyl)oxy)methyl)-3-isopropylhepta-4,6-dien-1-yl 4-methylbenzenesulfonate (±)-**13** (542 mg, 0.94 mmol, 1.0 eq.) in anhydrous THF (2.35 mL) was added *via* cannula followed by potassium iodide (78.0 mg, 0.47 mmol, 0.5 eq.). The mixture was warmed to room temperature, and then heated at 80 °C for 18 h. The mixture was cooled to room temperature, after which sat. aq. NH_4Cl solution (5 mL) was added and extracted with Et_2O (3 × 5 mL). The combined organic extracts were sequentially washed with water (15 mL), brine (15 mL), dried ($MgSO_4$), filtered and concentrated under reduced pressure. The residue was purified by flash column chromatography (10% EtOAc/petrol 30–40) to afford (*E*)-dimethyl 2-(6-(((*tert*-butyldiphenylsilyl)oxy)methyl)-3-isopropylhepta-4,6-dien-1-yl)malonate (±)-**14** as a colourless oil (385 mg, 0.71 mmol, 76%).

R_f = 0.29 (15% EtOAc/petrol 40-60)

δ_H (400 MHz, C_6D_6) 7.91 (4H, dd, *J* = 6.0, 2.6 Hz, Ar**H**), 7.35–7.29 (6H, m, Ar**H**), 6.15 (1H, d, *J* = 16.0 Hz, CH=C**H**C(=CH_2)), 5.60 (1H, s, C=C$\mathbf{H_2}$), 5.50 (1H, dd, *J* = 16.0, 9.4 Hz, CH(CH)C**H**=CH), 5.21 (1H, s, C=C$\mathbf{H_2}$), 4.62 (2H, s, C(=CH_2)C$\mathbf{H_2}$OTBDPS), 3.41 (1H, t, *J* = 7.5 Hz, $(MeO_2C)_2$C**H**CH_2), 3.40 (3H, s, OC$\mathbf{H_3}$), 3.39 (3H, s, OC$\mathbf{H_3}$), 2.18–1.96 (2H, m, CHC$\mathbf{H_2}$$CH_2$), 1.82–1.73 (1H, m, CH_2C**H**(CH)CH=), 1.57–

1.44 (2H, m, CHC**H**$(CH_3)_2$ and CH_2C**H$_2$**CH), 1.34–1.25 (1H, m, CH_2C**H$_2$**CH), 1.29 (9H, s, SiC(C**H$_3$**)$_3$), 0.89 (3H, d, *J* = 6.7, Hz, CH(C**H$_3$**)$_2$), 0.79 (3H, d, *J* = 6.7, Hz, CH(C**H$_3$**)$_2$)

δ_C (100 MHz, C_6D_6) 169.6 (**C**=O), 169.5 (**C**=O), 144.6 (**C**=CH_2), 135.9 (4C, Ar), 133.9 (2C, Ar), 131.7 (CH(CH)**C**H=CH), 131.6 (CH=**C**HC(=CH_2)), 130.0 (2C, Ar), 128.1 (4C, Ar), 113.7 (C=**C**H_2), 64.4 (C(=CH_2)**C**H_2OTBDPS), 51.9 (($MeO_2C)_2$**C**HCH_2), 51.8 (2C, O**C**H_3), 50.0 (CH_2**C**H(CH)CH=), 32.0 (CH**C**H$(CH_3)_2$), 30.2 (CH**C**H_2CH_2), 27.4 (CH_2C**H$_2$**CH), 26.9 (3C, SiC(**C**$H_3)_3$), 20.8 (CH(**C**$H_3)_2$), 19.5 (Si**C**$(CH_3)_3$), 19.1 (CH(**C**$H_3)_2$)

LRMS *m/z* (ESI^+) 559 ($[M+Na]^+$ 100%)

HRMS *m/z* (ESI^+) found 559.2838, $C_{32}H_{44}NaO_5Si$ requires 559.2850

ν_{max} (film)/cm^{-1} 2955 (m, CH), 2863 (m, CH), 1966 (w), 1896 (w), 1742 (s, C=O), 1605 (w), 1460 (m), 1436 (m), 1136 (m), 1248 (m), 1152 (m, C-O), 1106 (s), 1009 (m), 970 (m), 898 (m), 820 (s), 747 (m), 701 (s).

(m), 820 (s), 747 (m), 701 (s).

Asymmetric: To a stirred suspension of hexane washed NaH (180 mg, 7.54 mmol, 3.0 eq.), in anhydrous DMF (6.28 mL) cooled to 0 °C in an ice/water bath, was carefully added dropwise dimethyl malonate (996 mg, 7.54 mmol, 3.0 eq.). The resulting mixture was stirred for 20 min at 0 °C, after which a solution of (S,*E*)-6-(((*tert*-butyldiphenylsilyl)oxy)methyl)-3-isopropylhepta-4,6-dien-1-yl 4-methylbenzenesulfonate (–)-**13** (1.45 g, 2.51 mmol, 1.0 eq.) in anhydrous THF (6.28 mL) was added *via* cannula followed by potassium iodide (208 mg, 1.26 mmol, 0.5 eq.). The mixture was heated at 80 °C for 1.5 h and then cooled to room temperature. Sat. aq. NH_4Cl solution (20 mL) was added to the mixture and extracted with EtOAc (3 × 20 mL). The combined organic extracts were sequentially washed with water (60 mL), brine (60 mL), dried (Na_2SO_4), filtered and concentrated under reduced pressure. The residue was purified by flash column chromatography (10% EtOAc/petrol 40-60 with 1% triethylamine) to afford (S,*E*)-dimethyl 2-(6-(((*tert*-butyldiphenylsilyl)oxy)methyl)-3-isopropylhepta-4,6-dien-1-yl)malonate (+)-**14** as a colourless oil (1.12 g, 2.11 mmol, 84%, >99% ee (*S*)).

$[\alpha]_D^{20}$ -0.2 (*c* 1.01, DCM) for >99% ee

$[\alpha]_{365}^{20}$ +10.1 (*c* 1.01, DCM) for >99% ee

(3a*R*,6*S*,6a*S*)-Methyl 1-(3-((*tert*-butyldiphenylsilyl)oxy)prop-1-en-2-yl)-6-isopropyl-3-oxohexahydro-1*H*-cyclopenta[*c*]furan-3a-carboxylate 15

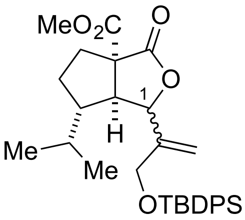

Racemic: A mixture of manganese(III) acetate (219 mg, 0.86 mmol, 2.0 eq.) and copper(II) triflate (148 mg, 0.43 mmol, 1.0 eq.) were pre heated to 80 °C, after which a solution of (*E*)-dimethyl 2-(6-(((*tert*-butyldiphenylsilyl)oxy)methyl)-3-isopropylhepta-4,6-dien-1-yl)malonate (±)-**14** (231 mg, 0.43 mmol, 1.0 eq.) in nitrogen sparged acetonitrile (2.15 mL, 0.2 M) was rapidly added *via* cannula. The resulting mixture was heated at 80 °C for 1 h, after which water (3 mL) and Et_2O (3 mL) were added and stirred for 30 min. The organic layer was separated and the aqueous layer was further extracted with Et_2O (2 × 3 mL). The combined organic layers were sequentially washed with sat. aq. $NaHCO_3$ solution (10 mL), brine (10 mL), dried ($MgSO_4$), filtered and concentrated under reduced pressure. The residue was purified by flash column chromatography (10% EtOAc/petrol 40-60) to afford (3*aR**,6*S**,6*aS**)-methyl 1-(3-((*tert*-butyldiphenylsilyl)oxy)prop-1-en-2-yl)-6-isopropyl-3-oxohexahydro-1*H*-cyclopenta[*c*]furan-3a-carboxylate (±)-**15** (diastereomeric ratio **15a**:**15b** = 6:1 – measured by HPLC) as a colourless oil (176 mg, 0.34 mmol, 79%). Characterisation is reported for the major epimer **15a** of an inseperable mixture of C-1 diastereomers, **15a** and **15b**. The ^{1}H NMR of **15** indicated the presence of another component, which we have been unable to characterise, but is possibly a C-6 diastereomer. The ratio of this component to the major C-1 diastereomer **15a** is approximately 20:1.

(1S*,3aR*,6S*,6aS*)-Methyl 1-(3-((tert-butyldiphenylsilyl)oxy)prop-1-en-2-yl)-6-isopropyl-3-oxohexahydro-1H-cyclopenta[c]furan-3a-carboxylate (±)-15a

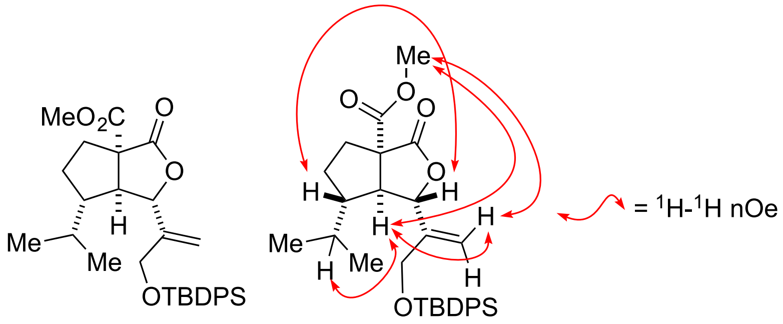

R_f = 0.21 (10% EtOAc/petrol 40-60)

δ_H (400 MHz, $CDCl_3$) 7.70–7.65 (4H, m, Ar**H**), 7.47–7.37 (6H, m, Ar**H**), 5.38 (1H, d, *J* = 0.8 Hz, C=C**H₂**), 5.24 (1H, d, *J* = 0.8 Hz, C=C**H₂**), 4.76 (1H, d, *J* = 3.7 Hz, CHC**H**(C=)O), 4.26 (2H, s, C(=CH_2)C**H₂**OTBDPS), 3.55 (3H, s, OC**H₃**), 2.84 (1H, dd, *J* = 5.3, 3.7 Hz, CHC**H**CHO), 2.48 (1H, dt, *J* = 13.0, 6.5 Hz, CH_2C**H₂**CC(=O)), 2.14–2.05 (1H, m, CH_2C**H₂**CC(=O)), 1.90–1.79 (1H, m, CH_2C**H₂**CH), 1.76–1.65 (2H, m, CH_2C**H₂**CH and CH_2C**H**(CH)CH), 1.55 (1H, dsept, *J* = 6.8, 6.8 Hz, CHC**H**(CH_3)₂), 1.08 (9H, s, SiC(C**H₃**)₃), 0.88 (3H, d, *J* = 6.8 Hz, CH(C**H₃**)₂), 0.86 (3H, d, *J* = 6.8 Hz, CH(C**H₃**)₂)

δ_C (100 MHz, $CDCl_3$) 175.6 (**C**=O), 171.0 (**C**O_2Me), 144.9 (**C**=CH_2), 135.5 (2C, Ar), 135.4 (2C, Ar), 133.2 (Ar), 133.0 (Ar), 129.8 (2C, Ar), 127.8 (4C, Ar), 112.1 (C=**C**H_2), 84.7 (CH**C**H(C=)O), 63.2 (C(=CH_2)**C**H_2OTBDPS), 61.6 (CH_2**C**(CO_2Me)C), 55.0 (CH_2**C**H(CH)CH), 54.3 (CH**C**HCHO), 52.8 (O**C**H_3), 34.2 (CH_2**C**H_2CC(=O)), 30.3 (CH**C**H(CH_3)₂), 29.6 (CH_2**C**H_2CH), 26.7 (3C, SiC(**C**H_3)₃), 21.7 (CH(**C**H_3)₂), 19.9 (CH(**C**H_3)₂), 19.2 (Si**C**(CH_3)₃)

LRMS *m/z* (ESI$^+$) 543 ([M+Na]$^+$, 50%), 580 (100%)

HRMS *m/z* (ESI$^+$) found 543.2528, $C_{31}H_{40}NaO_5Si$ requires 543.2537

ν_{max} (film)/cm^{-1} 3073 (w, =CH_2), 2959 (m, CH), 2932 (m, CH), 2859 (m, CH), 1776 (s, C=O), 1739 (s, C=O), 1239 (m, C-O), 1111 (s, Si-O-C), 907 (s, C=CH_2), 823 (m, Si-O-C).

Asymmetric: (3a*R*,6*S*,6a*S*)-Methyl 1-(3-((*tert*-butyldiphenylsilyl)oxy)prop-1-en-2-yl)-6-isopropyl-3-oxohexahydro-1*H*-cyclopenta[*c*]furan-3a-carboxylate (+)-**15**

A mixture of manganese(III) acetate (1.00 g, 3.76 mmol, 2.0 eq.) and copper(II) triflate (681 mg, 1.88 mmol, 1.0 eq.) were pre heated to 80 °C, after which a solution of (*S,E*)-dimethyl 2-(6-(((*tert*-butyldiphenylsilyl)oxy)methyl)-3-isopropylhepta-4,6-dien-1-yl)malonate (+)-**14** (1.01 g, 1.88 mmol, 1.0 eq.) in Ar sparged acetonitrile (9.40 mL) was rapidly added. The resulting mixture was heated at 80 °C for 30 min and then cooled to room temperature. Water (10 mL) and Et_2O (10 mL) were added and stirred for 10 min. The organic layer was separated and the aq. layer was further extracted with Et_2O (2 × 10 mL). The combined organic layers were sequentially washed with brine (30 mL), dried ($MgSO_4$), filtered and concentrated under reduced pressure. The residue was purified by flash column chromatography (10% EtOAc/petrol 40-60) to afford (3*aR*,6*S*,6*aS*)-methyl 1-(3-((*tert*-butyldiphenylsilyl)oxy)prop-1-en-2-yl)-6-isopropyl-3-oxohexahydro-1*H*-cyclopenta[*c*]furan-3a-carboxylate (+)-**15** (diastereomeric ratio = 6:1, measured by HPLC) as a colourless oil (720 mg, 1.39 mmol, 74%, >99% ee (*S*)). The ee was measured by HPLC (Chiralpak AD-H column, flow 0.6 mL/min, 1.0% IPA/hexane, 230 nm, t_1 = 8.4 (**15b** major and minor enantiomers) and 9.6 min (**15a** major enantiomer), t_2 = 11.0 (**15a** minor enantiomer); $[\alpha]_D^{20}$ +1.3 (*c* 0.98, $CHCl_3$), $[\alpha]_{365}^{20}$ +2.6 (*c* 0.98, $CHCl_3$) for >99% ee and a C-1 diastereomeric ratio of 6:1. The ^{1}H NMR of **15** indicated the presence of another component, which we have been unable to characterise, but is possibly a C-6 diastereomer. The ratio of this component to the major C-1 diastereomer **15a** is approximately 20:1.

*(3a*S*,4*S*,6a*R*)-3-(3-((*tert*-Butyldiphenylsilyl)oxy)prop-1-en-2-yl)-4-isopropylhexahydro-1*H*-cyclopenta[*c*]furan-1-one 16*

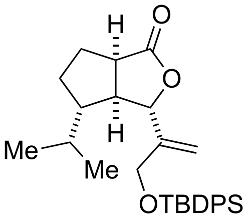

Racemic: To a stirred solution of (3a*R**,6*S**,6a*S**)-methyl 1-(3-((*tert*-butyldiphenylsilyl)oxy)prop-1-en-2-yl)-6-isopropyl-3-oxohexahydro-1*H*-cyclopenta[*c*]furan-3a-carboxylate (±)-**15** (103 mg, 0.20 mmol, 1.0 eq.) in dry DMF (2.00 mL) was added water (7.20 mg, 0.40 mmol, 2.0 eq.) and lithium chloride (16.7 mg, 0.40 mmol, 2.0 eq.). The resulting mixture was heated at 150 °C for 6 h and cooled to room temperature. Sat. aq. NH_4Cl

solution (2 mL) was added and extracted with Et_2O (3 × 2 mL). The combined organic extracts were sequentially washed with water (10 mL), brine (10 mL), dried (Na_2SO_4), filtered and concentrated under reduced pressure. The residue was purified by flash column chromatography (10% EtOAc/petrol 40–60) to afford (3a*S**,4*S**,6a*R**)-3-(3-((*tert*-butyldiphenylsilyl)oxy)prop-1-en-2-yl)-4-isopropylhexahydro-1*H*-cyclopenta[*c*]furan-1-one (±)-**16** (diastereomeric ratio = 6:1) as a colourless oil (80.0 mg, 0.17 mmol, 86%).

Characterisation is reported for the major and minor epimers as separated by flash column chromatography.

(3S*,3aS*,4S*,6aR*)-3-(3-((tert-Butyldiphenylsilyl)oxy)prop-1-en-2-yl)-4-isopropylhexahydro-1H-cyclopenta[c]furan-1-one (±)-16a

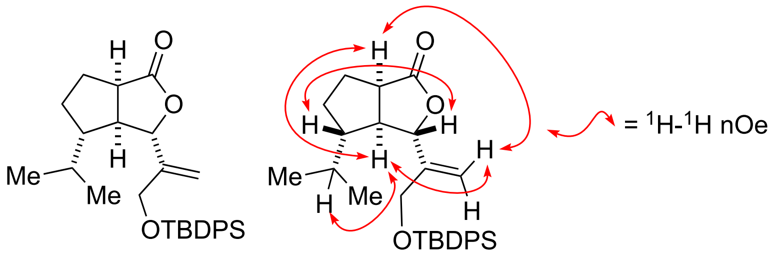

Major Epimer (±)-**16a**:

R_f = 0.38 (20% EtOAc/petrol 40-60)

δ_H (500 MHz, $CDCl_3$) 7.69–7.65 (4H, m, Ar**H**), 7.47–7.42 (2H, m, Ar**H**), 7.42–7.37 (4H, m, Ar**H**), 5.28 (1H, s, C=C**H₂**), 5.16 (1H, s, C=C**H₂**), 4.77 (1H, d, *J* = 2.6 Hz, CHC**H**(C=)O), 4.24 (1H, d, *J* = 13.9 Hz, C(=CH_2)C**H₂**OTBDPS), 4.18 (1H, d, *J* = 13.9 Hz, C(=CH_2)C**H₂**OTBDPS), 2.98 (1H, dt, *J* = 9.6, 4.9 Hz, CH_2C**H**C=O), 2.46 (1H, ddd, *J* = 9.6, 7.2, 2.6 Hz, CHC**H**CHO), 2.11–2.03 (1H, m, CH_2C**H₂**CHC=O), 1.93–1.86 (1H, m, CH_2C**H₂**CHC=O), 1.86–1.78 (1H, m, Hz, CH_2C**H₂**CH(*i*-Pr)), 1.66–1.59 (1H, m, CH_2C**H**(*i*-Pr)CH), 1.55 (1H, dsept, *J* = 6.7, 6.7 Hz, CHC**H**(CH_3)₂), 1.47–1.39 (1H, m, CH_2C**H₂**CH(*i*-Pr)), 1.07 (9H, s, SiC(C**H₃**)₃), 0.91 (3H, d, *J* = 6.7, Hz, CH(C**H₃**)₂), 0.86 (3H, d, *J* = 6.7, Hz, CH(C**H₃**)₂)

δ_C (125 MHz, $CDCl_3$) 180.6 (**C**=O), 145.8 (**C**=CH_2), 135.5 (2C, Ar), 135.4 (2C, Ar), 134.8 (Ar), 133.1 (Ar), 132.9 (Ar), 129.8 (Ar), 127.7 (2C, Ar), 127.7 (2C, Ar), 111.7 (C=**C**H₂), 84.5 (CH**C**H(C=)O), 63.5 (C(=CH_2)**C**H₂OTBDPS), 53.4 (CH_2**C**H(*i*-Pr)CH), 49.1 (CH**C**HCHO), 44.4 (CH_2**C**HC=O), 30.9

(CH**C**H(CH_3)$_2$), 29.6 (CH_2**C**H$_2$CH(*i*-Pr)), 28.3 (**C**H$_2$CHC=O), 26.7 (3C, SiC(**C**H$_3$)$_3$), 21.7 (CH(**C**H$_3$)$_2$), 19.9 (CH(**C**H$_3$)$_2$), 19.2 (Si**C**(CH_3)$_3$)

LRMS *m/z* (ESI^+) 331 (100%), 485 ($[M+Na]^+$, 80%), 947 ($[2M+Na]^+$, 65%)

HRMS *m/z* (ESI^+) found 485.2485 $C_{29}H_{38}NaO_3Si$ requires 485.2482

ν_{max} (film)/cm^{-1} 3071 (w, =CH_2), 2958 (m, CH), 2931 (m, CH), 2858 (m, CH), 1772 (s, C=O), 1257 (m, C-O), 1111 (s, Si-O-C), 915 (m, C=CH_2), 823 (m, Si-O-C).

(3R*,3aS*,4S*,6aR*)-3-(3-((tert-Butyldiphenylsilyl)oxy)prop-1-en-2-yl)-4-isopropylhexahydro-1H-cyclopenta[c]furan-1-one (±)-16b

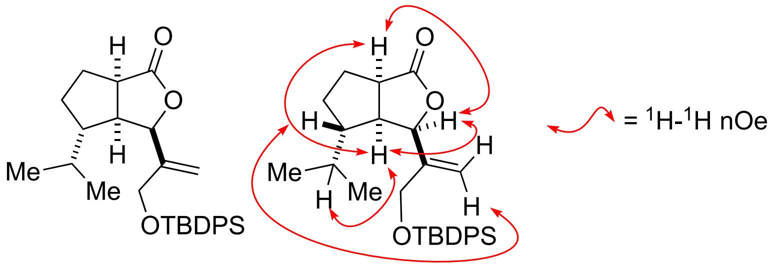

Minor Epimer (±)-**16b**:

R_f = 0.31 (20% EtOAc/petrol 40-60)

δ_H (500 MHz, $CDCl_3$) 7.70–7.64 (4H, m, Ar**H**), 7.48–7.43 (2H, m, Ar**H**), 7.42–7.38 (4H, m, Ar**H**), 5.39 (1H, s, C=C**H$_2$**), 5.29 (1H, s, C=C**H$_2$**), 5.09 (1H, d, *J* = 6.4 Hz, CHCHC**H**O), 4.16 (1H, d, *J* = 13.8 Hz, CC**H$_2$**OTBDPS), 4.10 (1H, d, *J* = 13.8 Hz, CC**H$_2$**OTBDPS), 3.09 (1H, ddd, *J* = 8.6, 8.6, 2.2 Hz, CH_2C**H**C=O), 2.56 (1H, ddd, *J* = 8.2, 6.4, 2.2 Hz, CHC**H**CHO), 2.07–2.00 (1H, m, CH_2C**H$_2$**CHC(=O)), 1.90–1.80 (1H, m, CH_2C**H$_2$**CHC(=O)), 1.78–1.71 (1H, m, CH_2C**H**(*i*-Pr)CH), 1.57–1.36 (3H, m, CH_2C**H$_2$**CH(*i*-Pr) & CHC**H**(CH_3)$_2$), 1.08 (9H, s, SiC(C**H$_3$**)$_3$), 0.76 (3H, d, *J* = 6.8, Hz, CH(C**H$_3$**)$_2$), 0.69 (3H, d, *J* = 6.8, Hz, CH(C**H$_3$**)$_2$)

δ_C (125 MHz, $CDCl_3$) 180.3 (**C**=O), 142.7 (**C**=CH_2), 135.5 (2C, Ar), 135.5 (2C, Ar), 133.1 (Ar), 132.9 (Ar), 129.9 (2C, Ar), 127.8 (2C, Ar), 127.8 (2C, Ar), 110.8 (C=**C**H$_2$), 80.0 (CHCH**C**HO), 64.2 (C**C**H$_2$OTBDPS), 47.5 (CH_2**C**HC=O), 46.5 (CH_2**C**H(*i*-Pr)CH), 45.3 (CH**C**HCHO), 29.7 (CH**C**H(CH_3)$_2$), 28.4

(CH_2**C**H_2CHC(=O)), 26.8 (3C, SiC(**C**H_3)$_3$), 26.3 (CH_2**C**H_2CH(*i*-Pr)), 22.0 (CH(**C**H_3)$_2$), 19.2 (Si**C**(CH_3)$_3$), 17.2 (CH(**C**H_3)$_2$)

LRMS *m/z* (ESI^+) 485 ($[M+Na]^+$, 60%), 947 ($[2M+Na]^+$, 100%)

HRMS *m/z* (ESI^+) found 485.2478, $C_{29}H_{38}NaO_3Si$ requires 485.2482

ν_{max} (film)/cm^{-1} 3071 (w, =CH_2), 2958 (m, CH), 2931 (m, CH), 2858 (m, CH), 1774 (s, C=O), 1660 (w), 1589 (w), 1471 (m), 1428 (m), 1389 (m), 1363 (m), 1257 (m, C-O), 1112 (s, Si-O-C), 1017 (m), 911 (m, C=CH_2), 823 (m, Si-O-C).

Asymmetric: To a stirred solution of (3a*R*,6*S*,6a*S*)-methyl 1-(3-((*tert*-butyldiphenylsilyl)oxy)prop-1-en-2-yl)-6-isopropyl-3-oxohexahydro-1*H*-cyclopenta[*c*]furan-3a-carboxylate (+)-**15** (702 mg, 1.35 mmol, 1.0 eq.) in anhydrous DMF was added water (48.6 mg, 2.70 mmol, 2.0 eq.) and lithium chloride (114 mg, 2.70 mmol, 2.0 eq.). The resulting mixture was heated at 150 °C for 6 h then cooled to room temperature. Sat. aq. NH_4Cl solution (20 mL) was added to the mixture and extracted with Et_2O (3 × 10 mL). The combined organic extracts were sequentially washed with water (30 mL), brine (30 mL), dried ($MgSO_4$), filtered and concentrated under reduced pressure. The residue was purified by flash column chromatography (10-20% gradient EtOAc/petrol 40-60) to afford (3a*S*,4*S*,6a*R*)-3-(3-((*tert*-butyldiphenylsilyl)oxy)prop-1-en-2-yl)-4-isopropylhexahydro-1*H*-cyclopenta[*c*]furan-1-one (–)-**16** (diastereomeric ratio = 6:1) as a colourless oil (541 mg, 1.16 mmol, 86%, >99% ee).

Major Epimer (–)-**16a**:

(3S,3aS,4S,6aR)-3-(3-((tert-Butyldiphenylsilyl)oxy)prop-1-en-2-yl)-4-isopropylhexahydro-1H-cyclopenta[c]furan-1-one (–)-16a

$[\alpha]_D^{20}$ -6.6 (*c* 0.96, $CHCl_3$), $[\alpha]_{365}^{20}$ -20.4 (*c* 0.96, $CHCl_3$) for >99% ee

Minor Epimer (–)-**16b**:

(3R,3aS,4S,6aR)-3-(3-((tert-Butyldiphenylsilyl)oxy)prop-1-en-2-yl)-4-isopropylhexahydro-1H-cyclopenta[c]furan-1-one (–)-16b

$[\alpha]_D^{20}$ -23.0 (*c* 1.00, $CHCl_3$), $[\alpha]_{365}^{20}$ -76.9 (*c* 1.00, $CHCl_3$) for >99% ee

(3S,3aS*,4S*,6aR*)-3-(3-((tert-Butyldiphenylsilyl)oxy)prop-1-en-2-yl)-4-isopropyl-6a-methylhexahydro-1H-cyclopenta[c]furan-1-one (±)-17*

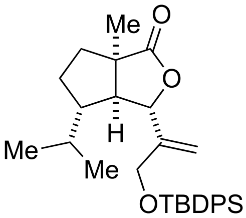

Racemic: To a stirred solution of (3*S**,3a*S**,4*S**,6a*R**)-3-(3-((*tert*-butyldiphenylsilyl)oxy)prop-1-en-2-yl)-4-isopropylhexahydro-1*H*-cyclopenta[*c*]furan-1-one (±)-**16a** (320 mg, 0.69 mmol, 1.0 eq.) in anhydrous THF (13.8 mL) cooled to -78 °C in a acetone/dry ice bath was added methyl iodide (491 mg, 3.46 mmol, 5.0 eq.) followed by dropwise addition of a 1 M solution of LiHMDS in toluene (3.46 mL, 3.46 mmol, 5.0 eq.). After complete addition the mixture was stirred for a further 30 min at -78 °C. Sat. aq. NH_4Cl solution (10 mL) was added to the mixture and extracted with Et_2O (3 × 10 mL). The combined organic extracts were sequentially washed brine (30 mL), dried (Na_2SO_4), filtered and concentrated under reduced pressure. The residue was purified by flash column chromatography (10% EtOAc/petrol 40–60) to afford (3*S**,3a*S**,4*S**,6a*R**)-3-(3-((*tert*-butyldiphenylsilyl)oxy)prop-1-en-2-yl)-4-isopropyl-6a-methylhexahydro-1*H*-cyclopenta[*c*]furan-1-one (±)-**17** as a colourless oil (296 mg, 0.62 mmol, 90%).

R_f = 0.21 (10% EtOAc/petrol 40-60)

δ_H (500 MHz, $CDCl_3$) 7.70–7.65 (4H, m, Ar**H**), 7.47–7.36 (6H, m, Ar**H**), 5.33 (1H, s, C=C**H₂**), 5.22 (1H, s, C=C**H₂**), 4.65 (1H, d, *J* = 3.9 Hz, CHC**H**(C)O), 4.21 (2H, s, CC**H₂**OTBDPS), 2.17–2.14 (1H, m, CHC**H**(C)CHO), 2.01 (1H, ddd, *J* = 13.1, 8.7, 7.0 Hz, CH_2C**H₂**C(Me)), 1.81–1.74 (1H, m, CH_2C**H₂**CH(*i*-Pr)), 1.73–1.64 (2H, m, CH_2C**H₂**C(Me) & CH_2C**H**(*i*-Pr)CH), 1.59–1.49 (2H, m, CH_2C**H₂**CH(*i*-Pr) & CHC**H**(CH_3)₂), 1.25 (3H, s, C(C**H₃**)), 1.08 (9H, s, SiC(C**H₃**)₃), 0.87 (3H, d, *J* = 6.7 Hz, CH(C**H₃**)₂), 0.84 (3H, d, *J* = 6.7 Hz, CH(C**H₃**)₂)

δ_C (125 MHz, $CDCl_3$) 182.6 (C=O), 145.9 (**C**=CH_2), 135.5 (2C, Ar), 135.4 (2C, Ar), 133.1 (Ar), 133.0 (Ar), 129.8 (2C, Ar), 127.8 (2C, Ar), 127.7 (2C, Ar), 111.2 (C=**C**H_2), 84.0 (CH**C**H(C)O), 63.4 (C**C**H_2OTBDPS), 55.5 (CH_2**C**H(*i*-Pr)CH), 55.1 (CH**C**H(C)CHO), 51.1 (CH_2**C**(Me)C), 37.8 ($CH_2$$CH_2$C(Me)), 30.8

(CH**C**H(CH_3)$_2$), 29.0 (CH_2**C**H_2CH(*i*-Pr)), 26.7 (3C, SiC(**C**H_3)$_3$), 24.2 (C(**C**H_3)), 21.8 (CH(**C**H_3)$_2$), 19.9 (CH(**C**H_3)$_2$), 19.2 (Si**C**(CH_3)$_3$)

LRMS *m/z* (ESI^+) 499 ($[M+Na]^+$, 49%), 975 ($[2M+Na]^+$, 100%)

HRMS *m/z* (ESI^+) found 499.2636, $C_{30}H_{40}NaO_3Si$ requires 499.2639

ν_{max} (film)/cm^{-1} 3071 (w, =CH_2), 2958 (s, CH), 2932 (m, CH), 2858 (m, CH), 1771 (s, C=O), 1658 (w), 1590 (w), 1471 (m), 1428 (m), 1389 (m), 1374 (m), 1337 (w), 1307 (w), 1255 (w), 1209 (w), 1162 (w, C-O), 1110 (s, Si-O-C), 1056 (m), 1010 (m), 914 (m, C=CH_2), 823 (m, Si-O-C), 741 (m), 702 (s).

Asymmetric: (3*S*,3a*S*,4*S*,6a*R**-3-(3-((*tert*-Butyldiphenylsilyl)oxy)prop-1-en-2-yl)-4-isopropyl-6a-methylhexahydro-1*H*-cyclopenta[*c*]furan-1-one (–)-**17**

To a vigourously stirred solution of (3*S*,3a*S*,4*S*,6a*R*)-3-(3-((*tert*-butyldiphenylsilyl)oxy)prop-1-en-2-yl)-4-isopropylhexahydro-1*H*-cyclopenta[*c*]furan-1-one (–)-**16a** (450 mg, 0.97 mmol, 1.0 eq.) in anhydrous THF (19.4 mL) cooled to -78 °C in a dry ice/acetone bath was added methyl iodide (691 mg, 4.86 mmol, 5.0 eq.) followed by dropwise addition of a 1 M solution of LiHMDS in toluene (4.86 mL, 4.86 mmol, 5.0 eq.). The resulting mixture was stirred for 30 min at -78 °C. Sat. aq. NH_4Cl solution (20 mL) was added to the mixture and extracted with Et_2O (3 × 10 mL). The combined organic extracts were sequentially washed with brine (30 mL), dried ($MgSO_4$), filtered and concentrated under reduced pressure. The residue was purified by flash column chromatography (10% EtOAc/petrol 40-60) to afford (3*S*,3a*S*,4*S*,6a*R*)-3-(3-((*tert*-butyldiphenylsilyl)oxy)prop-1-en-2-yl)-4-isopropyl-6a-methylhexahydro-1*H*-cyclopenta[*c*]furan-1-one (–)-**17** as a colourless oil (414 mg, 0.87 mmol, 90%, >99% ee). The ee was measured by HPLC (Chiralcel OD column, flow 0.6 mL/min, 1.0% IPA/hexane, 230 nm, t_1 = 15.2 min (major enantiomer), t_2 = 16.5 min (minor enantiomer);

$[\alpha]_D^{20}$ -7.5 (*c* 1.00, $CHCl_3$).

$[\alpha]_{365}^{20}$ -23.3 (*c* 1.00, $CHCl_3$) for >99% ee

tert-Butyl((2-((1S,3aR,6S,6aS)-6-isopropyl-3a-methyl-3-methylenehexahydro-1H-cyclopenta[c]furan-1-yl)allyl)oxy)diphenylsilane **18**

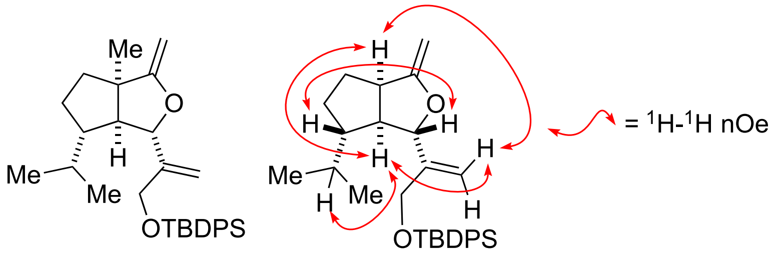

Racemic: To (3*S**,3*aS**,4*S**,6*aR**)-3-(3-((*tert*-butyldiphenylsilyl)oxy)prop-1-en-2-yl)-4-isopropyl-6a-methylhexahydro-1*H*-cyclopenta[*c*]furan-1-one (±)-**17** (141 mg, 0.30 mmol, 1.0 eq.) was added a 0.24 M solution of the Petasis reagent in toluene (3.06 mL, 153 mg, 0.73 mmol, 2.4 eq.). The resulting mixture was covered and heated at 110 °C for 30 min. The mixture was concentrated under reduced pressure to afford a red oil which was suspended in hexane (10 mL). Celite (5.00 g) was added to the mixture and stirred for 30 min. The mixture was filtered and concentrated under reduced pressure. The residue was purified by flash column chromatography (10% EtOAc/petrol 40–60 with 1% triethylamine) to afford *tert*-butyl((2-((1*S**,3a*R**,6*S**,6a*S**)-6-isopropyl-3a-methyl-3-methylenehexahydro-1*H*-cyclopenta[*c*]furan-1-yl)allyl)oxy)diphenylsilane (±)-**18** (as a colourless oil (122 mg, 0.26 mmol, 89%).

R_f = 0.15 (10% EtOAc/petrol 40-60)

δ_H (500 MHz, C_6D_6) 7.93–7.88 (4H, m, Ar**H**), 7.36–7.30 (6H, m, Ar**H**), 5.61 (1H, dd, *J* = 3.0, 1.4 Hz, CHC(=C**H$_2$**)CH$_2$), 5.43 (1H, dt, *J* = 3.0, 1.4 Hz, CHC(=C**H$_2$**)CH$_2$), 4.60 (1H, d, *J* = 1.4 Hz, CC(=C**H$_2$**)O), 4.56 (1H, d, *J* = 5.1 Hz, CHC**H**(C)O), 4.51 (1H, t, *J* = 1.4 Hz, CC**H$_2$**OTBDPS), 4.49 (1H, t, *J* = 1.4 Hz, CC**H$_2$**OTBDPS), 3.96 (1H, d, *J* = 1.4 Hz, CC(=C**H$_2$**)O), 2.05 (1H, dd, *J* = 5.1, 5.1 Hz, CHC**H**(C)CHO), 1.92 (1H, ddd, *J* = 12.5, 10.1, 6.8 Hz, (C)C**H$_2$**CH$_2$CH), 1.75 (1H, dddd, *J* = 12.5, 6.8, 6.8, 4.0 Hz, (C)CH$_2$C**H$_2$**CH), 1.62 (1H, ddd, *J* = 12.5, 6.8, 4.0 Hz, (C)C**H$_2$**CH$_2$CH), 1.55–1.48 (1H, m, CH$_2$C**H**(*i*-Pr)CH), 1.44–1.34 (2H, m, CHC**H**(CH$_3$)$_2$ and (C)CH$_2$C**H$_2$**CH), 1.29 (9H, s, SiC(C**H$_3$**)$_3$), 1.23 (3H, s, C(C**H$_3$**)), 0.88 (3H, d, *J* = 6.6 Hz, CH(C**H$_3$**)$_2$), 0.86 (3H, d, *J* = 6.6 Hz, CH(C**H$_3$**)$_2$)

δ_C (125 MHz, C_6D_6) 173.1 (C**C**(=CH$_2$)O), 148.3 (CHC(=**C**H$_2$)CH$_2$), 135.9 (2C, Ar), 135.9 (2C, Ar), 133.9 (Ar), 133.7 (Ar), 130.1 (2C, Ar), 128.3 (Ar), 128.1 (2C, Ar), 127.9 (Ar), 110.6 (CHC(=**C**H$_2$)CH$_2$), 87.8 (CH**C**H(C)O), 78.1 (CHC(=**C**H$_2$)CH$_2$), 63.9 (C**C**H$_2$OTBDPS), 58.7 (CH**C**H(C)CHO), 54.6 (CH$_2$**C**H(*i*-

Pr)CH), 53.8 (CH_2**C**(Me)C), 41.6 ((C)**C**H_2CH_2CH), 32.1 (CH**C**H$(CH_3)_2$), 30.5 ((C)CH_2**C**H_2CH), 27.1 (C(**C**H_3)), 27.0 (3C, SiC(**C**H_3)$_3$), 22.2 (CH(**C**H_3)$_2$), 20.3 (CH(**C**H_3)$_2$), 19.5 (Si**C**$(CH_3)_3$)

LRMS *m/z* (ESI^+) 475 ($[M+H]^+$, 35%), 497 ($[M+Na]^+$, 100%)

HRMS *m/z* (ESI^+) found 497.2845, $C_{31}H_{42}NaO_2Si$ requires 497.2846

ν_{max} (film)/cm^{-1} 3235 (w), 2958 (m, CH), 2859 (m, CH), 2280 (s, C=CH_2)1667 (m, C=CH_2), 1618 (m), 1453 (m), 1428 (m), 1390 (w), 1330 (s), 1162 (w), 1112 (m, Si-O-C), 1053 (w), 914 (w), 812 (s, Si-O-C), 741 (m), 704 (s).

Asymmetric: *tert*-Butyl((2-((1*S*,3a*R*,6*S*,6a*S*)-6-isopropyl-3a-methyl-3-methylenehexahydro-1H-cyclopenta[c]furan-1-yl)allyl)oxy)diphenylsilane (–)-**18**

A sealed tube was charged with (3*S*,3*aS*,4*S*,6*aR*)-3-(3-((*tert*-butyldiphenylsilyl)oxy)prop-1-en-2-yl)-4-isopropyl-6a-methylhexahydro-1*H*-cyclopenta[*c*]furan-1-one (–)-**17** (47.7 mg, 0.10 mmol, 1.0 eq.) and a 0.24 M solution of the Petasis reagent in toluene (1.25 mL, 0.30 mmol, 3.0 eq.). The resulting mixture was covered and heated at 110 °C for 1 h, then cooled to room temperatre. Hexane (10 mL) and Celite were added to the mixture and stirred for 30 min, then filtered through a Celite plug washing with hexane (2 × 10 mL). The filtrate was concentrated under reduced pressure. The residue was purified by flash column chromatography (0-15% EtOAc/petrol 40-60 with 1% triethylamine) to afford *tert*-butyl((2-((1*S*,3a*R*,6*S*,6a*S*)-6-isopropyl-3a-methyl-3-methylenehexahydro-1*H*-cyclopenta[*c*]furan-1-yl)allyl)oxy)diphenylsilane (–)-**18** as a pale orange oil (39.9 mg, 0.08 mmol, 84%, >99% ee).

$[\alpha]_D^{20}$ -3.2 (*c* 1.00, $CHCl_3$) for >99% ee

*(1***S***,3a***R***,8a***R***)-7-(((***tert***-Butyldiphenylsilyl)oxy)methyl)-1-isopropyl-3a-methyl-1,3,3a,5,6,8a-hexahydroazulen-4(2***H***)-one 19*

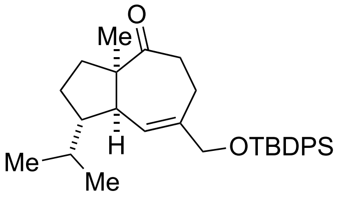

Racemic: A solution of *tert*-butyl((2-((1*S**,3*aR**,6*S**,6*aS**)-6-isopropyl-3a-methyl-3-methylenehexahydro-1*H*-cyclopenta[*c*]furan-1-yl)allyl)oxy)diphenylsilane (±)-**18** (104 mg, 0.21 mmol, 1.0 eq.) in anhydrous xylene (4.20 mL) was heated at 150 °C for 16 H and then concentrated under reduced pressure. The residue was purified by flash column chromatography (5% EtOAc/petrol 40–60) to afford (1*S**,3*aR**,8*aR**)-7-(((*tert*-butyldiphenylsilyl)oxy)methyl)-1-isopropyl-3a-methyl-1,3,3a,5,6,8a-hexahydroazulen-4(2*H*)-one (±)-**19** as a colourless oil (78 mg, 0.16 mmol, 76%).

R_f = 0.28 (10% EtOAc/petrol 40-60)

δ_H (500 MHz, $CDCl_3$) 7.68–7.64 (4H, m, Ar**H**), 7.46–7.35 (6H, m, Ar**H**), 5.56 (1H, d, *J* = 4.2 Hz, CHC**H**=C), 4.08 (2H, s, CC**H**$_2$OTBDPS), 2.80–2.74 (1H, m, C(=O)C**H**$_2$CH$_2$), 2.54–2.37 (2H, m, =C(CH$_2$)C**H**$_2$CH$_2$ & C(=O)C**H**$_2$CH$_2$), 2.32–2.27 (1H, m, CHC**H**CH=), 2.22–2.15 (1H, m, =C(CH$_2$)C**H**$_2$CH$_2$), 2.15–2.07 (1H, m, CH$_2$C**H**$_2$C(Me)), 1.84–1.76 (1H, m, CH$_2$C**H**$_2$CH(*i*-Pr)), 1.69–1.61 (1H, m, CH$_2$C**H**(*i*-Pr)CH), 1.61–1.53 (1H, m, CHC**H**(CH$_3$)$_2$), 1.42–1.29 (2H, m, CH$_2$C**H**$_2$C(Me) & CH$_2$C**H**$_2$CH(*i*-Pr)), 1.27 (3H, s, C(C**H**$_3$)), 1.06 (9H, s, SiC(C**H**$_3$)$_3$), 0.93 (3H, d, *J* = 6.6 Hz, CH(C**H**$_3$)$_2$), 0.91 (3H, d, *J* = 6.6 Hz, CH(C**H**$_3$)$_2$)

δ_C (125 MHz, $CDCl_3$) 213.5 (**C**=O), 140.7 (CH=**C**(C)CH$_2$), 135.5 (4C, Ar), 133.6 (CH**C**H=C), 131.0 (2C, Ar), 129.6 (2C, Ar), 127.6 (4C, Ar), 67.3 (C**C**H$_2$OTBDPS), 58.8 (CH$_2$**C**(CH$_3$)C=O), 56.0 (CH$_2$**C**H(*i*-Pr)CH), 51.3 (CH**C**HCH=), 39.6 (C(=O)**C**H$_2$CH$_2$), 34.7 (CH$_2$**C**H$_2$C(Me)), 33.1 (CH**C**H(CH$_3$)$_2$), 27.1 (CH$_2$**C**H$_2$CH(*i*-Pr)), 26.8 (3C, SiC(**C**H$_3$)$_3$), 24.8 (=C(CH$_2$)**C**H$_2$CH$_2$), 24.7 (C(**C**H$_3$)), 22.0 (CH(**C**H$_3$)$_2$), 20.0 (CH(**C**H$_3$)$_2$), 19.2 (Si**C**(CH$_3$)$_3$)

LRMS *m/z* (ESI$^+$) 497 ([M+Na]$^+$, 97%), 971 ([2M+Na]$^+$, 100%)

HRMS *m/z* (ESI$^+$) found 497.2851, $C_{31}H_{42}NaO_2Si$ requires 497.2846

ν_{max} (film)/cm^{-1} 3071 (w, =CH), 3049 (w, =CH), 2956 (s, CH), 2858 (s, CH), 1959 (w, Ph overtone), 1891 (w, Ph overtone), 1824 (w, Ph overtone), 1699 (s, C=O), 1462 (m), 1428 (m), 1369 (w), 1254 (w), 1111 (s, Si-O-C), 1062 (m), 915 (m, C=C-H), 823 (m, Si-O-C).

Asymmetric: (1*S*,3a*R*,8a*R*)-7-(((*tert*-Butyldiphenylsilyl)oxy)methyl)-1-isopropyl-3a-methyl-1,3,3a,5,6,8a-hexahydroazulen-4(2H)-one (+)-**19**

A stirred solution of *tert*-butyl((2-((1*S*,3*aR*,6*S*,6*aS*)-6-isopropyl-3a-methyl-3-methylenehexahydro-1*H*-cyclopenta[*c*]furan-1-yl)allyl)oxy)diphenylsilane (–)-**18** (35.0 mg, 0.07 mmol, 1.0 eq.) in anhydrous xylene (1.47 mL) was heated at 150 °C for 18 h, then cooled to room temperature. The mixtue was concentrated under reduced pressure. The residue was purified by flash column chromatography (10% EtOAc/petrol 40-60) to afford (1*S*,3*aR*,8*aR*)-7-(((*tert*-butyldiphenylsilyl)oxy)methyl)-1-isopropyl-3a-methyl-1,3,3a,5,6,8a-hexahydroazulen-4(2*H*)-one (+)-**19** as a colourless oil (25.7 mg, 0.05 mmol, 77%, >99% ee).

One-pot: A sealed tube was charged with (3*S*,3a*S*,4*S*,6a*R*)-3-(3-((*tert*-butyldiphenylsilyl)oxy)prop-1-en-2-yl)-4-isopropyl-6a-methylhexahydro-1*H*-cyclopenta[*c*]furan-1-one (–)-**18** (23.8 mg, 0.05 mmol, 1.0 eq.) and a 0.24 M solution of the Petasis reagent in toluene (0.65 mL, 0.15 mmol, 3.0 eq.). The resulting mixture was covered and heated at 110 °C for 1 h, then cooled to room temperature. Celite and a few drops of hexane were added to the mixture to destroy residual Petasis reagent. The resulting mixture was then heated at 150 °C for 18 h and cooled to room temperature and concentrated under reduced pressure. The residue was purified by flash column chromatography (10% EtOAc/petrol 40-60) to afford (1*S*,3*aR*,8*aR*)-7-(((*tert*-butyldiphenylsilyl)oxy)methyl)-1-isopropyl-3a-methyl-1,3,3a,5,6,8a-hexahydroazulen-4(2*H*)-one (+)-**19** as a colourless oil (17.6 mg, 0.03 mmol, 74%, >99% *ee*).

$[\alpha]_D^{20}$ +22.9 (*c* 1.00, $CHCl_3$) for >99% ee

Aphanamol-I

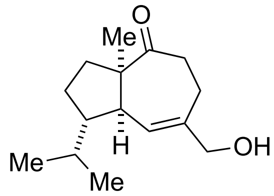

Racemic: To a stirred solution of (1*S**,3*aR**,8*aR**)-7-(((*tert*-butyldiphenylsilyl)oxy)methyl)-1-isopropyl-3a-methyl-1,3,3a,5,6,8a-hexahydroazulen-4(2*H*)-one (±)-**19** (47.4 mg, 0.10 mmol, 1.0 eq.) in anhydrous THF (0.50 mL) cooled to 0 °C in a ice/water bath was added acetic acid (24.0 mg, 0.40 mmol, 4.0 eq.) followed by dropwise addition of a 1 M solution of TBAF in THF (0.20 mL, 0.20 mmol, 2.0 eq.). The resulting mixture was warmed to room temperature and stirred for 4 h. Sat. aq. NH_4Cl solution (10 mL) was added and extracted with EtOAc (3 × 10 mL). The combined organic extracts were sequentially washed with brine (30 mL), dried ($MgSO_4$), filtered and concentrated under reduced pressure. The residue was purified by flash column chromatography (40% EtOAc/petrol 40–60) to afford (±)-aphanamol-I (±)-**1** as a colourless oil (23.5 mg, 0.10 mmol, 100%).

R_f = 0.22 (40% EtOAc/petrol 40-60)

δ_H (500 MHz, $CDCl_3$) 5.53 (1H, d, *J* = 4.5 Hz, C(H)C**H**=C), 4.03 (2H, d, *J* = 2.3 Hz, CC$\mathbf{H_2}$OH), 2.83 (1H, ddd, *J* = 14.6, 5.8, 3.8 Hz, C(=O)C$\mathbf{H_2}$$CH_2$), 2.60–2.51 (1H, m, =C($CH_2$)C$\mathbf{H_2}$$CH_2$), 2.43 (1H, ddd, *J* = 14.6, 11.8, 5.8 Hz, C(=O)C$\mathbf{H_2}$$CH_2$), 2.32–2.25 (2H, m, =C($CH_2$)C$\mathbf{H_2}$$CH_2$ & CHC**H**CH=), 2.14–2.05 (1H, m, CH_2C$\mathbf{H_2}$C(Me)), 1.85–1.75 (1H, m, CH_2C$\mathbf{H_2}$CH(*i*-Pr)), 1.71–1.63 (1H, m, CH_2C**H**(CH)CH), 1.63–1.53 (1H, m, CHC**H**(CH_3)$_2$), 1.42–1.29 (3H, m, CH_2C$\mathbf{H_2}$C(Me), CH_2O**H** & CH_2C$\mathbf{H_2}$CH(*i*-Pr)), 1.27 (3H, s, C(C$\mathbf{H_3}$)), 0.92 (3H, d, *J* = 6.5 Hz, CH(C$\mathbf{H_3}$)$_2$), 0.91 (3H, d, *J* = 6.5 Hz, CH(C$\mathbf{H_3}$)$_2$)

δ_C (125 MHz, $CDCl_3$) 213.6 (**C**=O), 141.6 (CH=**C**(C)CH_2), 132.9 (C(H)**C**H=C), 67.1 (C**C**H_2OH), 58.9 (CH_2**C**(CH_3)C=O), 56.0 (CH_2**C**H(CH)CH), 51.4 (CH**C**HCH=), 39.9 (C(=O)**C**$H_2$$CH_2$), 34.5 ($CH_2$**C**$H_2$C(Me)), 33.0 (CH**C**H($CH_3$)$_2$), 27.0 ($CH_2$**C**$H_2$CH(*i*-Pr)), 24.9 (=C($CH_2$)**C**$H_2$$CH_2$), 24.7 (C(**C**$H_3$)), 22.0 (CH(**C**$H_3$)$_2$), 19.9 (CH(**C**$H_3$)$_2$)

LRMS *m*/*z* (ESI^+) 259 ([M+Na]$^+$, 60%), 495 ([2M+Na]$^+$, 100%)

HRMS *m*/*z* (ESI^+) found 259.1670, $C_{15}H_{24}NaO_2$ requires 259.1669

ν_{max} (film)/cm^{-1} 3418 (br, OH), 2960 (m, CH), 2872 (m, CH), 1691 (s, C=O), 1463 (m), 1420 (w), 1386 (w), 1374 (w), 1255 (w), 1174 (w), 1075 (m), 1000 (m). Data are consistent with literature values.

Asymmetric: To a stirred solution of (1*S*,3*aR*,8*aR*)-7-(((*tert*-butyldiphenylsilyl)oxy)methyl)-1-isopropyl-3a-methyl-1,3,3a,5,6,8a-hexahydroazulen-4(2*H*)-one (+)-**19** (14.2 mg, 0.03 mmol, 1.0 eq.) in anhydrous THF (0.50 mL) cooled to 0 °C in a ice/water bath was added acetic acid (7.81 mg, 0.13 mmol, 4.0 eq.) followed by dropwise addition of a 1 M solution of TBAF in THF (60.0 μL, 0.06 mmol, 2.0 eq.). The resulting mixture was warmed to room temperature and stirred for 24 h. 1 M HCl (10 mL) was added to the mixture and extracted with Et_2O (3 × 10 mL). The combined organic extracts were sequentially washed with brine (30 mL), dried ($MgSO_4$), filtered and concentrated under reduced pressure. The residue was purified by flash column chromatography (40% EtOAc/petrol 40-60) to afford (+)-aphanamol-I (+)-**1** as a colourless oil (7.1 mg, 0.03 mmol, 100%, >99% ee (*S*)).

$[\alpha]_D^{20}$ +23.3 (*c* 0.40, $CHCl_3$) for >99% ee,

lit. synthetic enantiopure material $[\alpha]_D^{20}$ +23.0 (*c* 0.40, $CHCl_3$)[6]; $[\alpha]_D^{20}$ +23.0 (*c* 2.00, $CHCl_3$)[7];

lit. natural material $[\alpha]_D^{18}$ +13.7 (*c* 0.29, $CHCl_3$)[8] – Wickberg has previously postulated that natural aphanamol I was isolated in 50% enantiomeric excess.[7]

1.3 NMR Spectra and HPLC Traces

Dimethyl 2-(2-methylpropylidene)malonate ***6***

^{1}H NMR (400 MHz, $CDCl_3$)

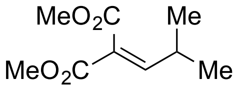

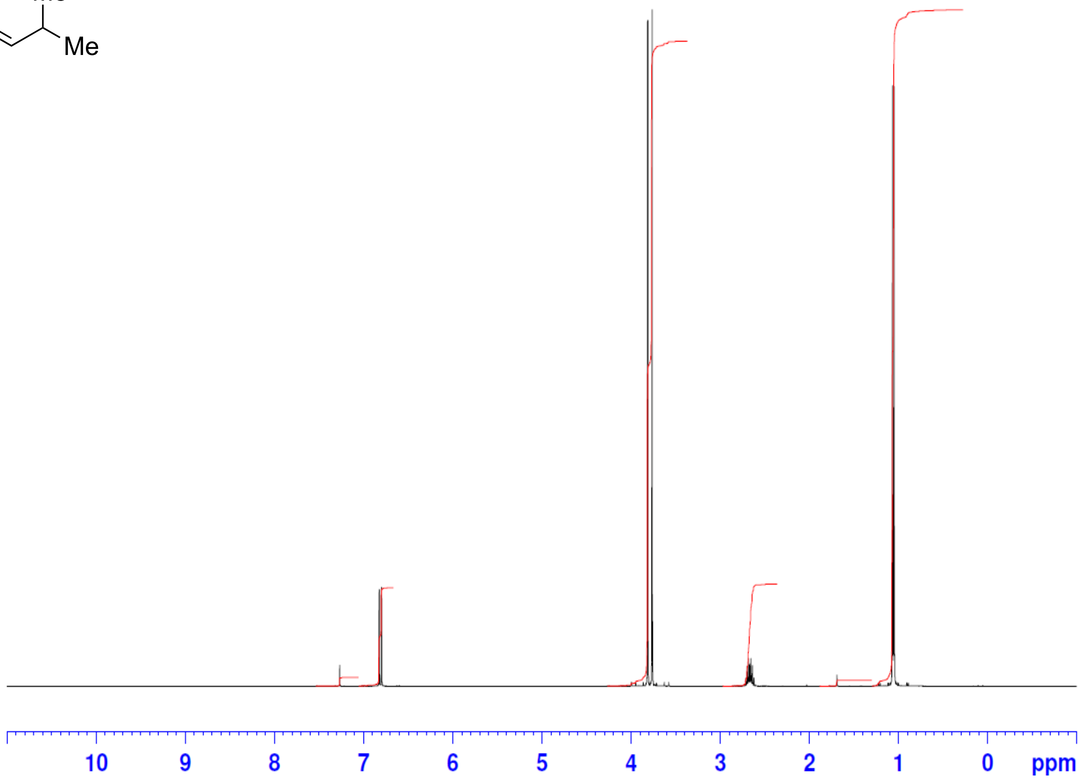

^{13}C NMR (100 MHz, $CDCl_3$)

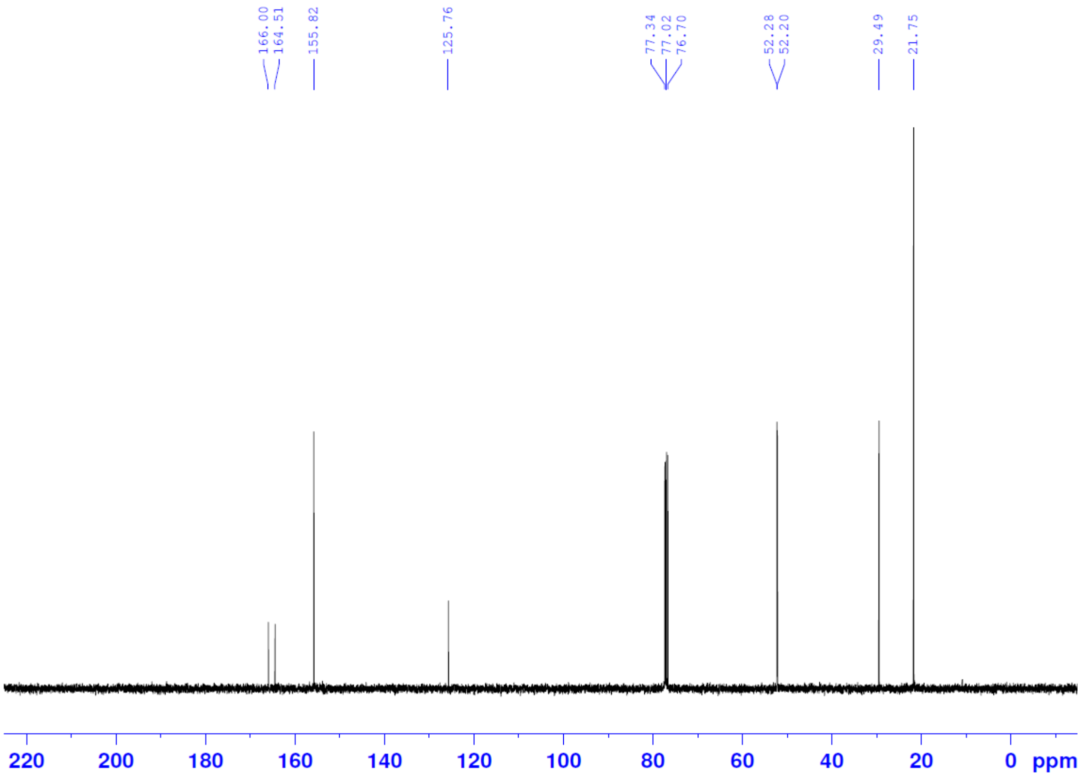

*(±)-Dimethyl 2-(4-methyl-1-(trimethylsilyl)pent-1-yn-3-yl)malonate (±)-**8***

^{1}H NMR (400 MHz, $CDCl_3$)

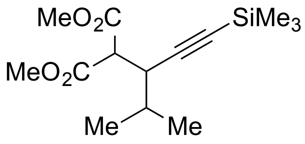

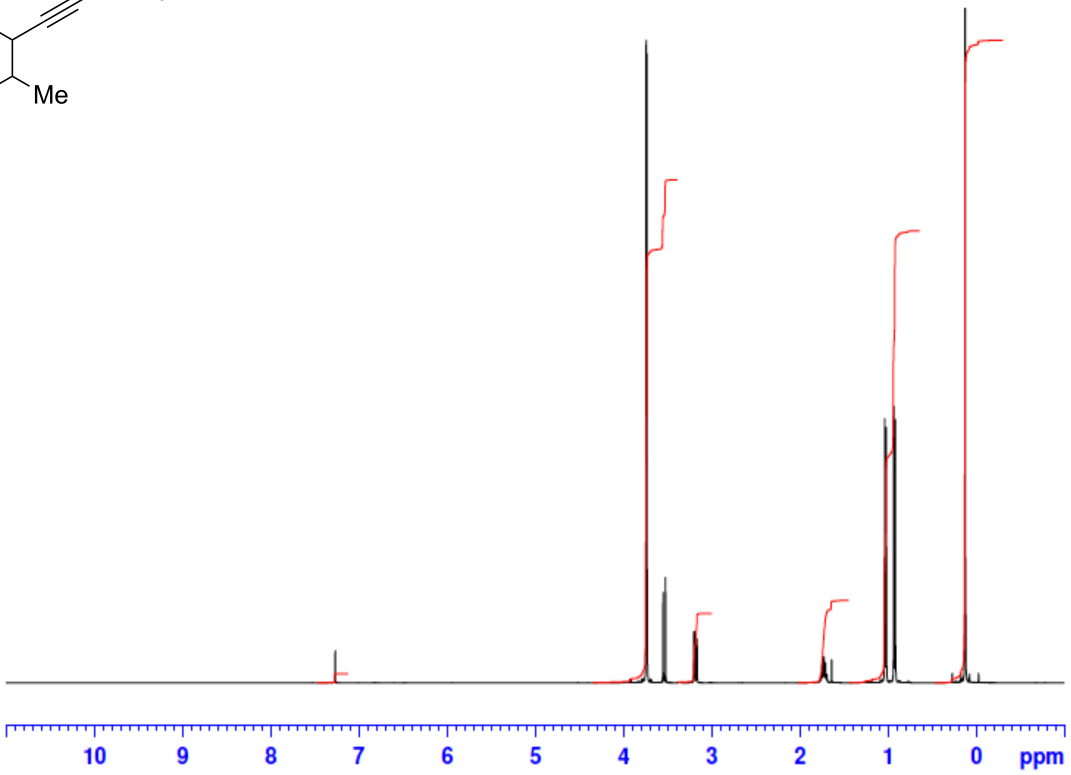

^{13}C NMR (100 MHz, $CDCl_3$)

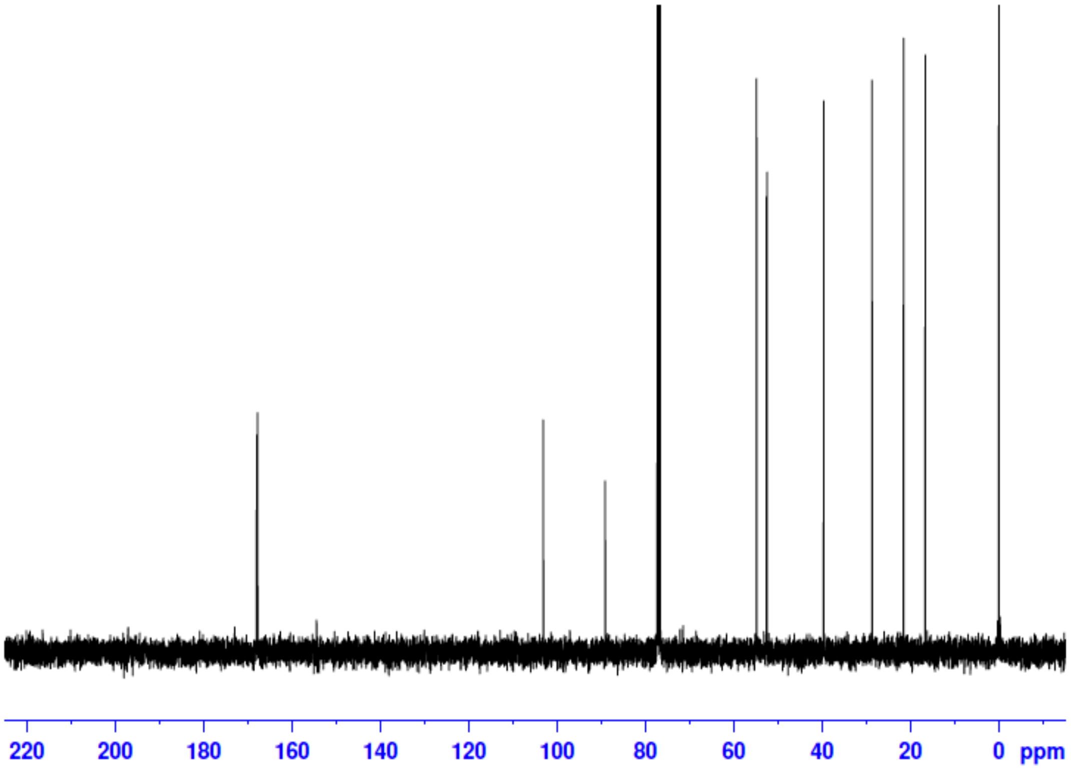

(±)-Methyl 3-isopropyl-5-(trimethylsilyl)pent-4-ynoate ***(±)-9***

^{1}H NMR (400 MHz, $CDCl_3$)

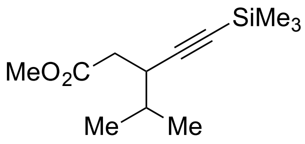

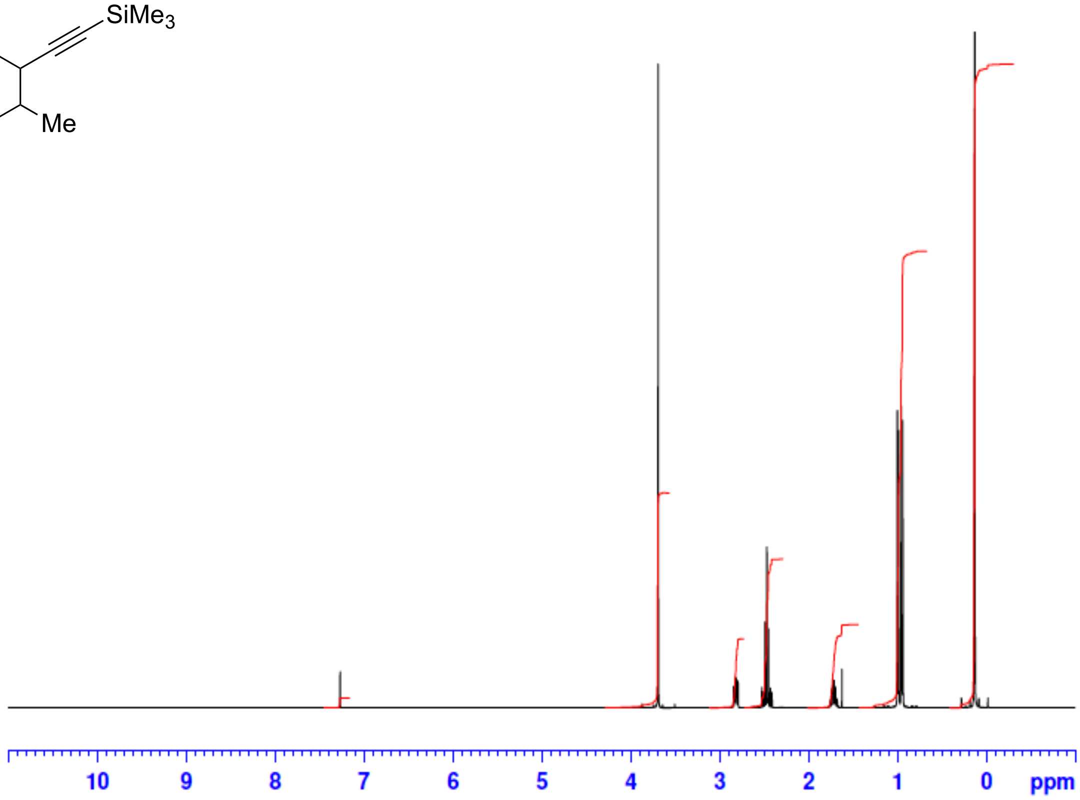

^{13}C NMR (100 MHz, $CDCl_3$)

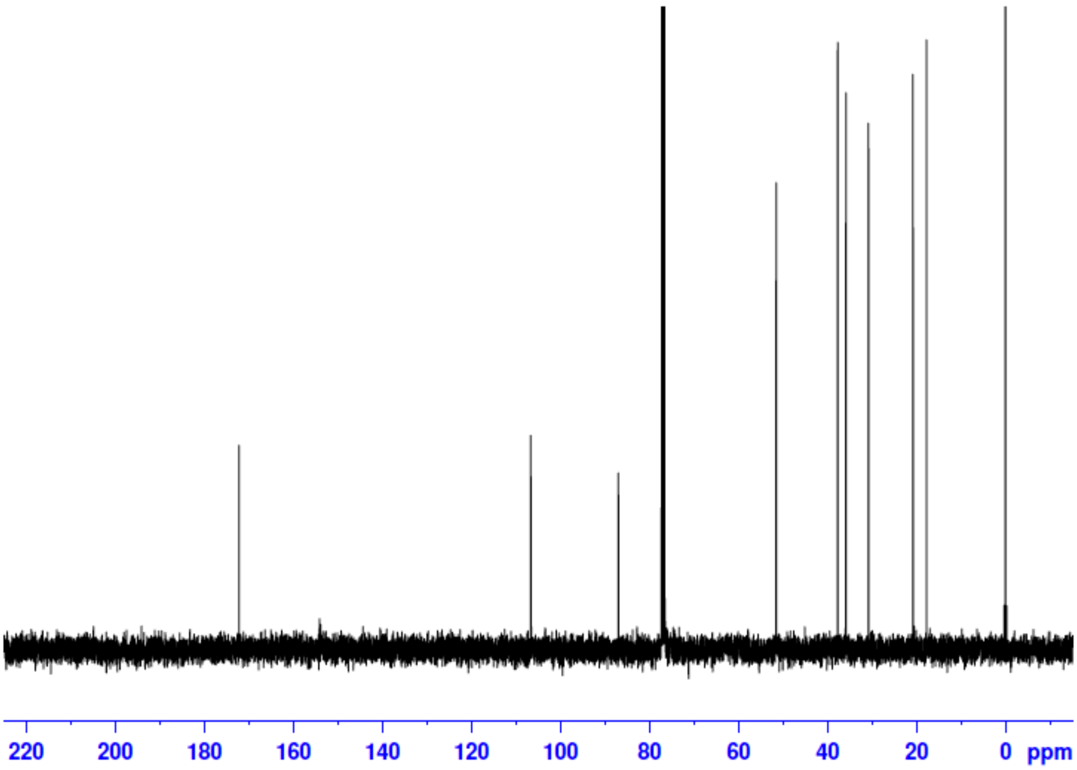

(±)-3-Isopropyl-5-(trimethylsilyl)pent-4-yn-1-ol ***(±)-10***

^{1}H NMR (400 MHz, $CDCl_3$)

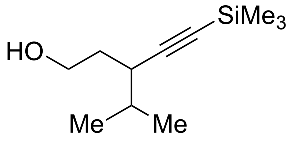

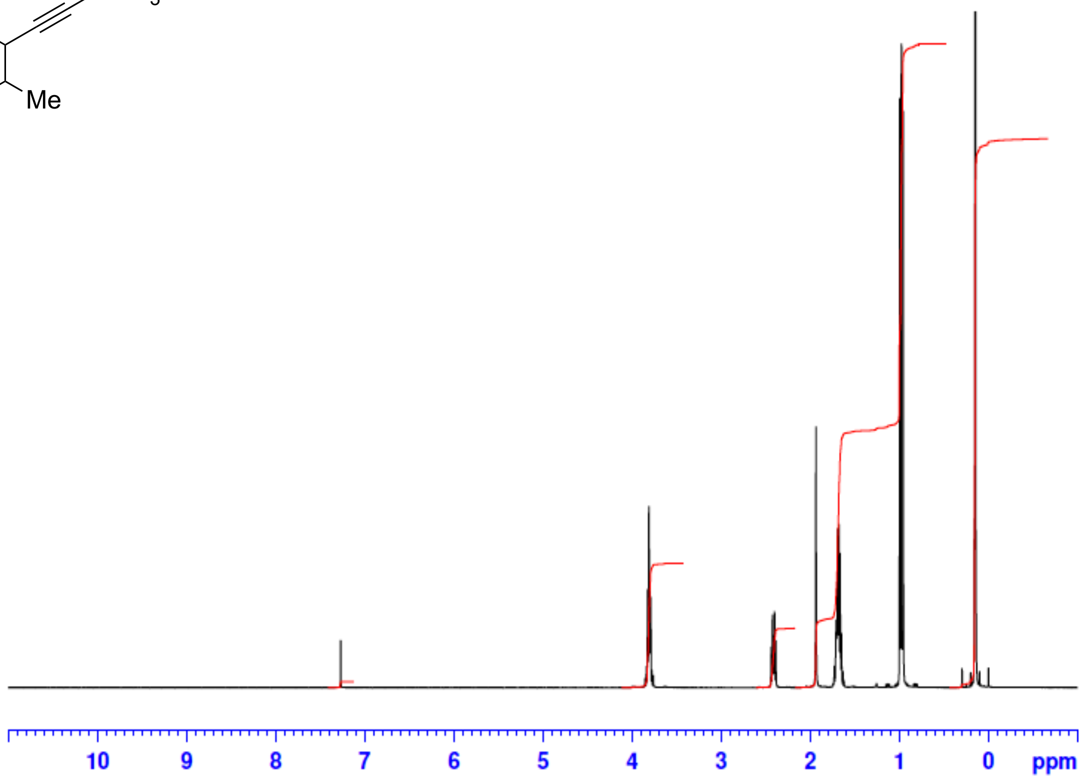

^{13}C NMR (100 MHz, $CDCl_3$)

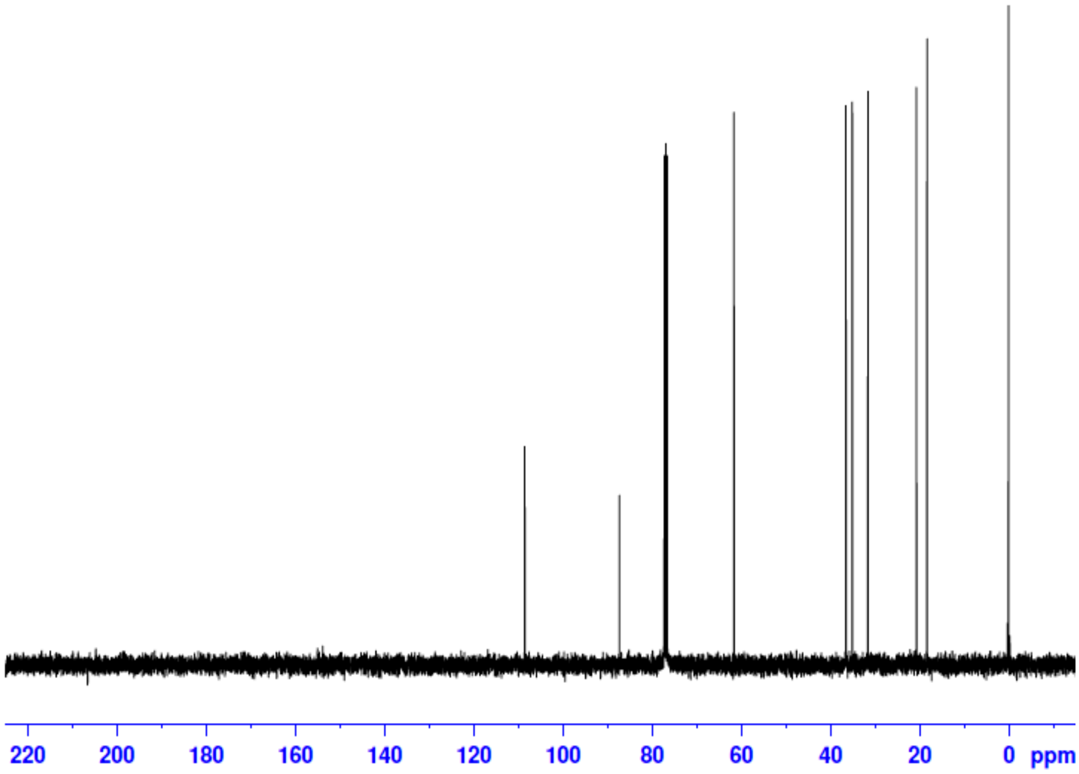

(±)-3-Isopropylpent-4-yn-1-ol

^{1}H NMR (400 MHz, $CDCl_3$)

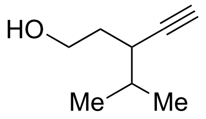

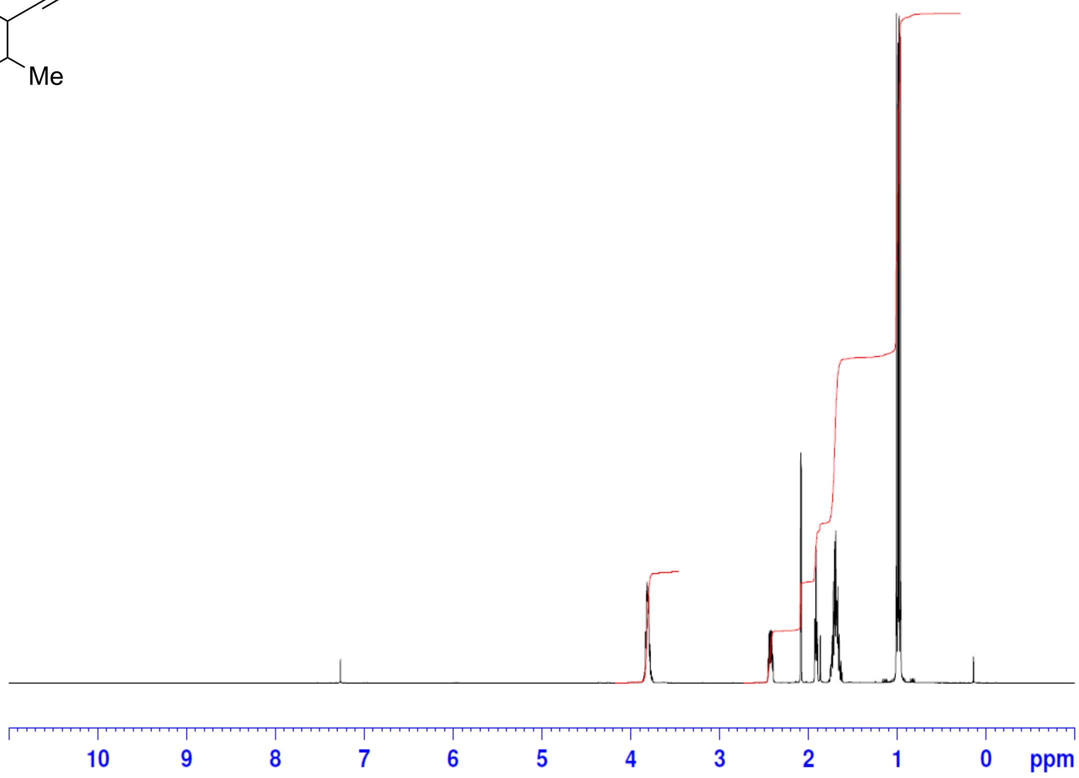

^{13}C NMR (100 MHz, $CDCl_3$)

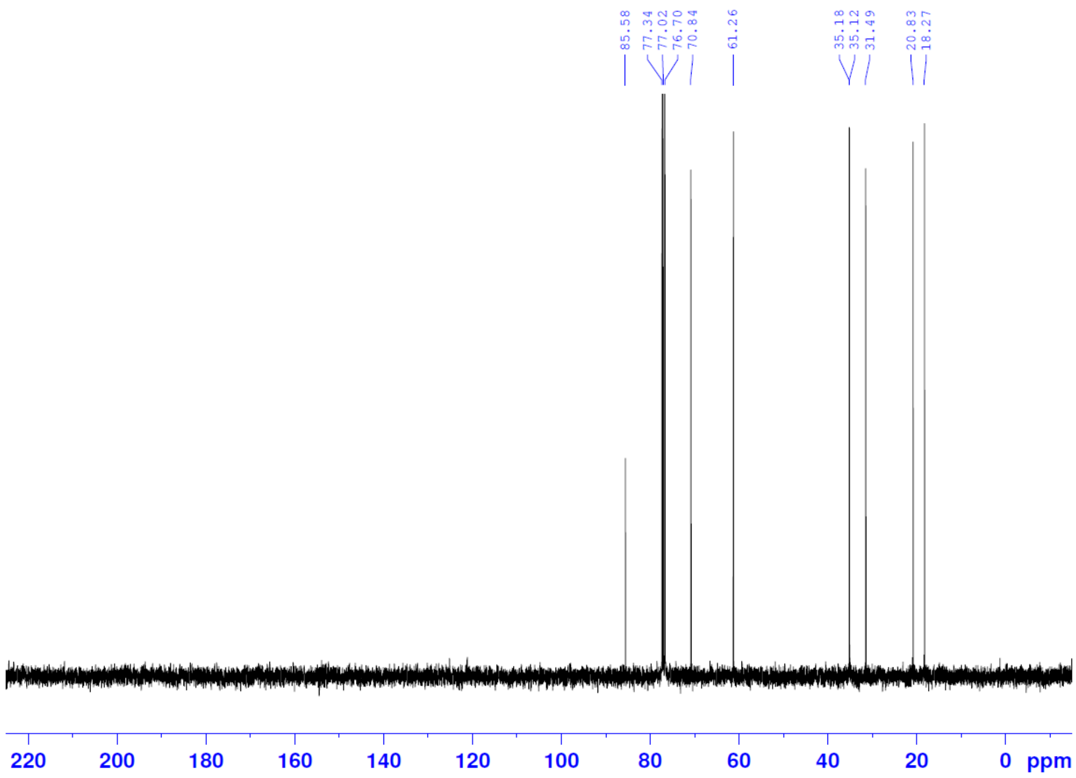

*(S)-3-Isopropyl-5-(triisopropylsilyl)pent-4-yn-1-ol (–)-**24***

^{1}H NMR (500 MHz, $CDCl_3$)

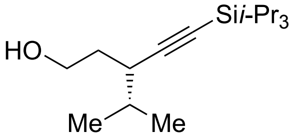

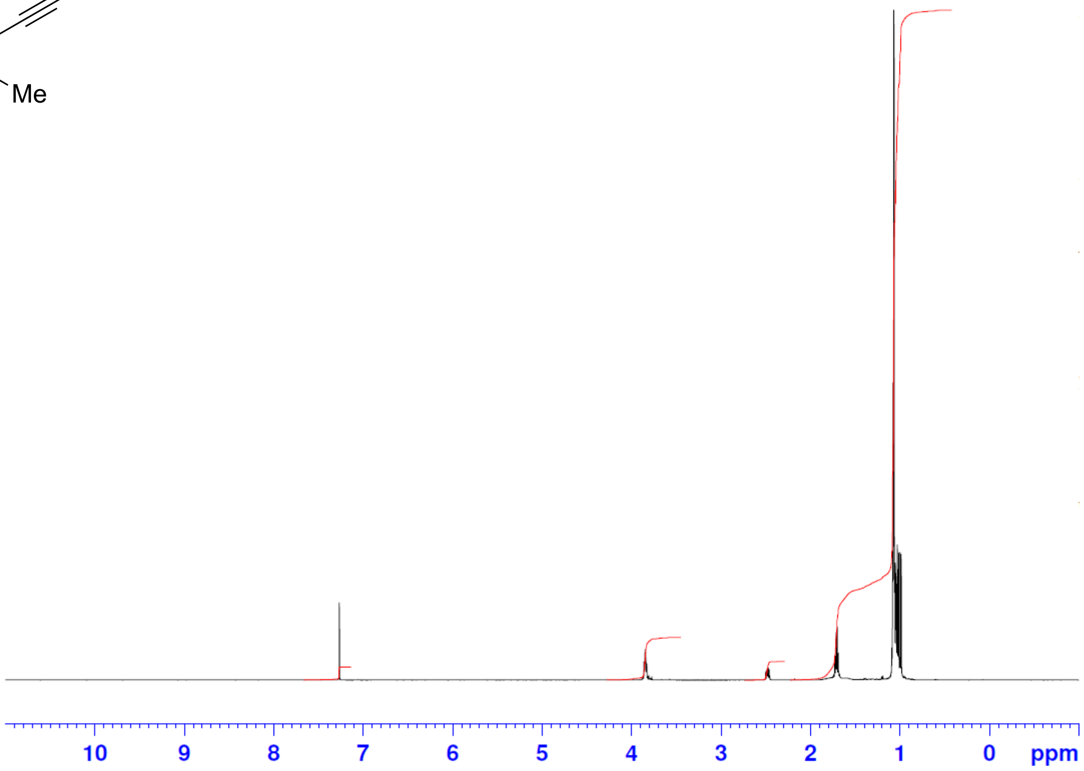

^{13}C NMR (125 MHz, $CDCl_3$)

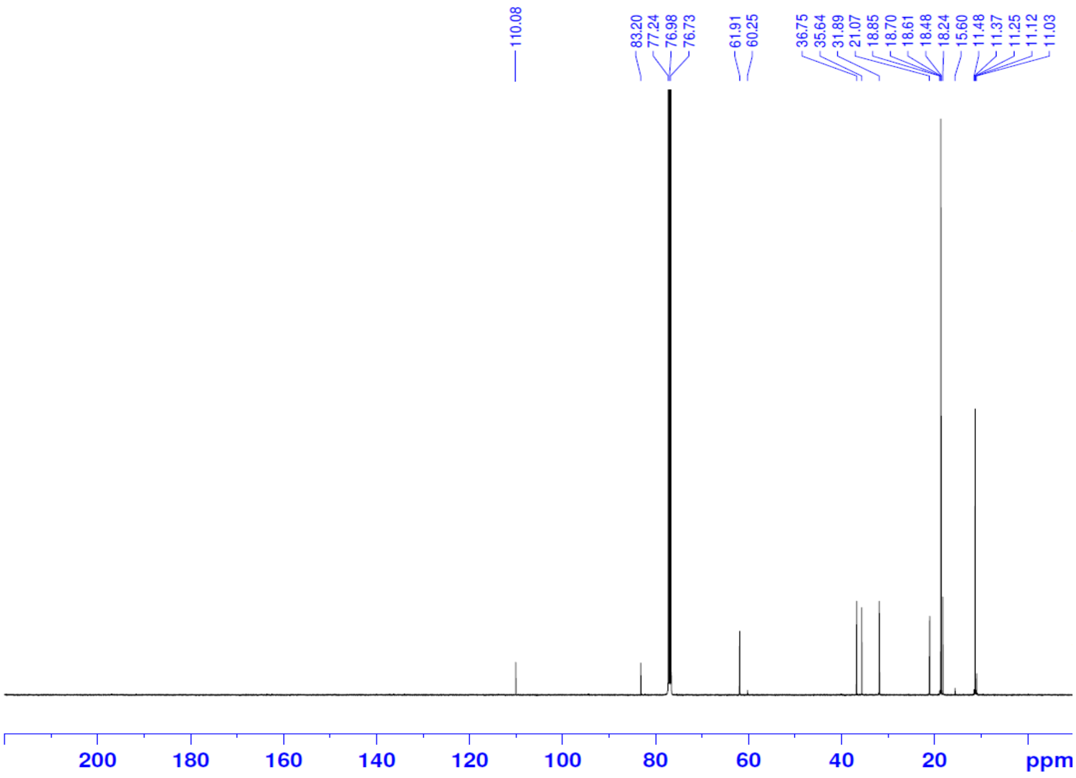

(S)-3-Isopropyl-5-(triisopropylsilyl)pent-4-yn-1-yl benzoate

^{1}H NMR (500 MHz, $CDCl_3$)

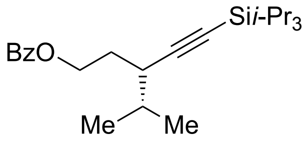

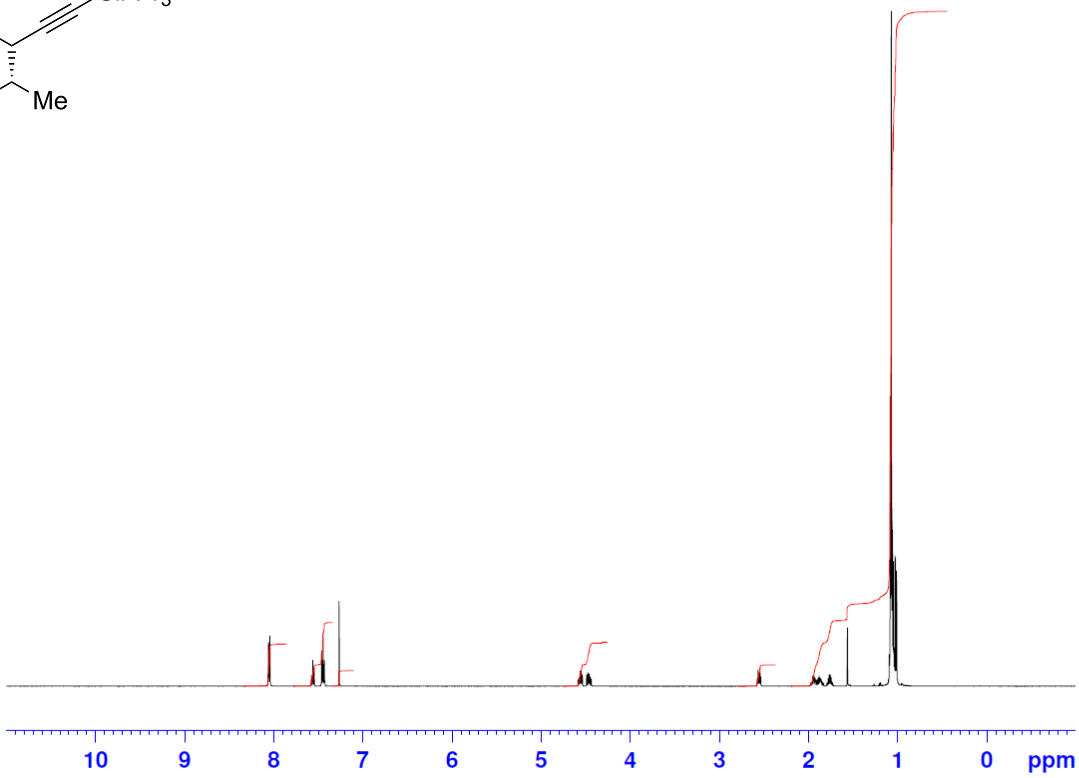

^{13}C NMR (125 MHz, $CDCl_3$)

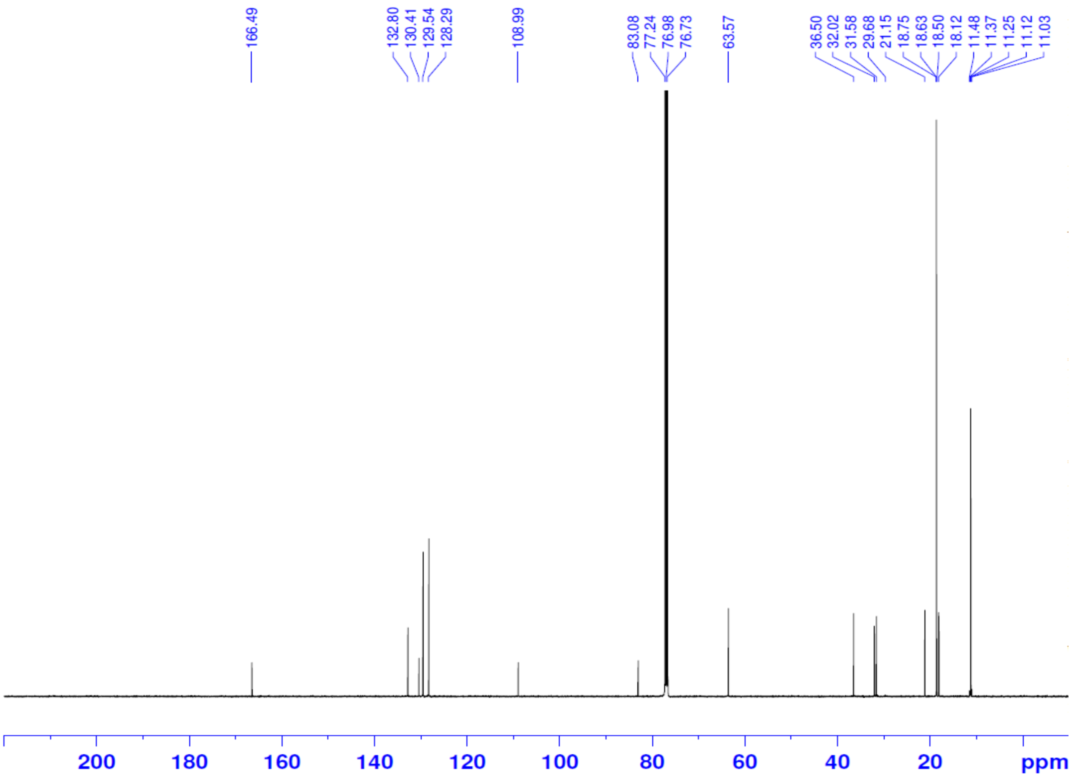

HPLC (Chiial OD column, flow 0.6 ml/min 0.2% IPA/hexane 230 nm) for ee determination .

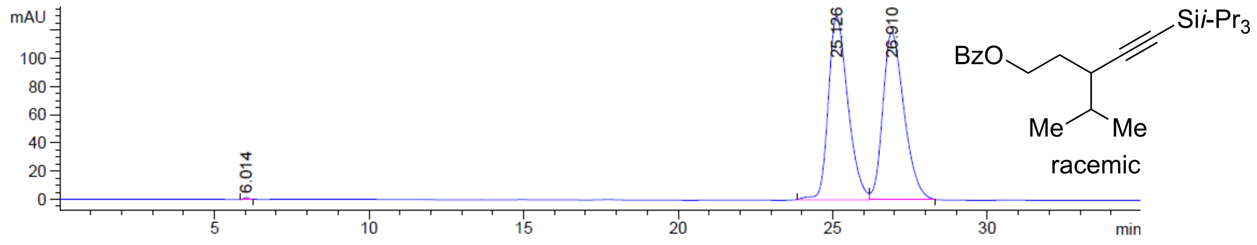

Signal 4: DAD1 D, Sig=230,16 Ref=360,100

Peak #	RetTime [min]	Type	Width [min]	Area [mAU*s]	Height [mAU]	Area %
1	6.014	BB	0.1530	12.85503	1.28172	0.1126
2	25.126	BV	0.6722	5736.58496	130.15561	50.2548
3	26.910	VB	0.7312	5665.55811	118.62328	49.6326

Totals : 1.14150e4 250.06061

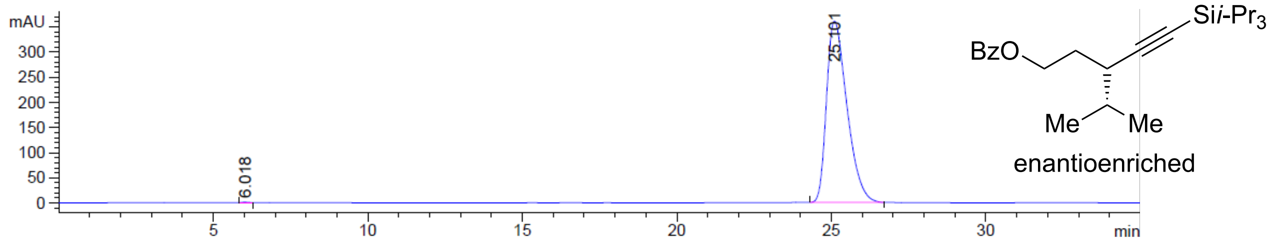

Signal 4: DAD1 D, Sig=230,16 Ref=360,100

Peak #	RetTime [min]	Type	Width [min]	Area [mAU*s]	Height [mAU]	Area %
1	6.018	BB	0.1525	13.19739	1.32118	0.0797
2	25.101	BB	0.7059	1.65458e4	360.19833	99.9203

Totals : 1.65590e4 361.51952

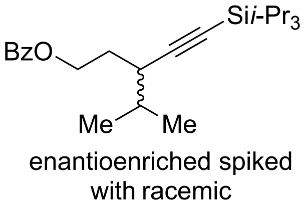

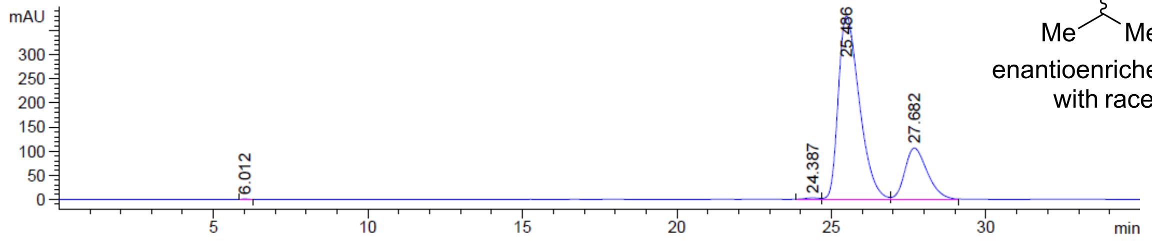

Signal 4: DAD1 D, Sig=230,16 Ref=360,100

Peak #	RetTime [min]	Type	Width [min]	Area [mAU*s]	Height [mAU]	Area %
1	6.012	BB	0.1553	12.62923	1.23535	0.0538
2	24.387	BV	0.4649	100.87181	3.08996	0.4301
3	25.486	VB	0.7285	1.79951e4	379.95398	76.7228
4	27.682	BB	0.7740	5346.12305	106.08109	22.7933

Totals : 2.34548e4 490.36037

*(±)-3-Isopropylpent-4-yn-1-yl 4-methylbenzenesulfonate (±)-**11***

^{1}H NMR (400 MHz, $CDCl_3$)

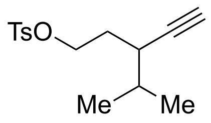

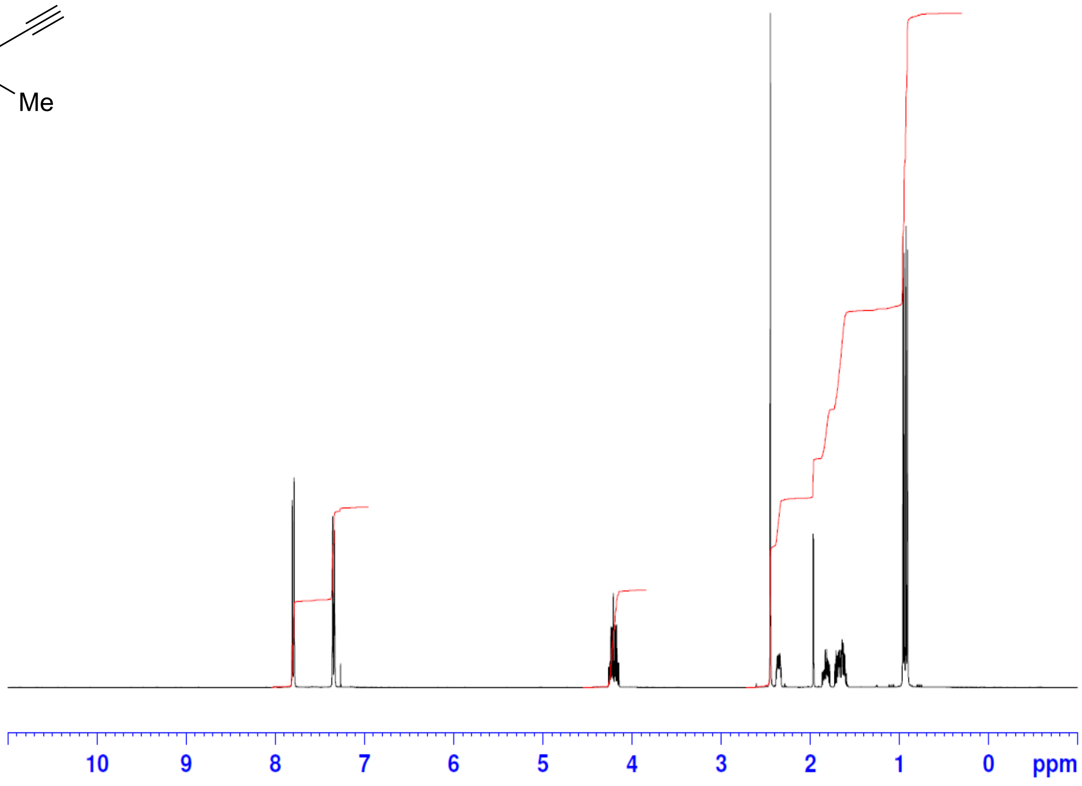

^{13}C NMR (100 MHz, $CDCl_3$)

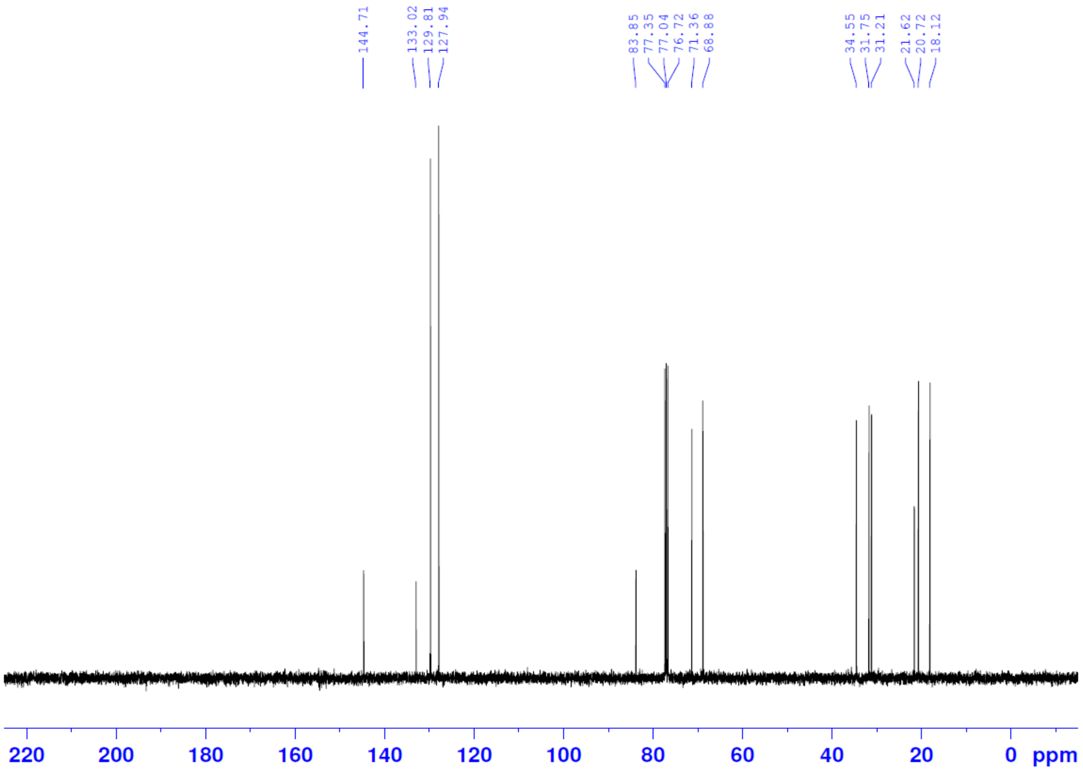

2-Iodoprop-2-en-1-ol

^{1}H NMR (400 MHz, $CDCl_3$)

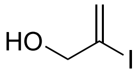

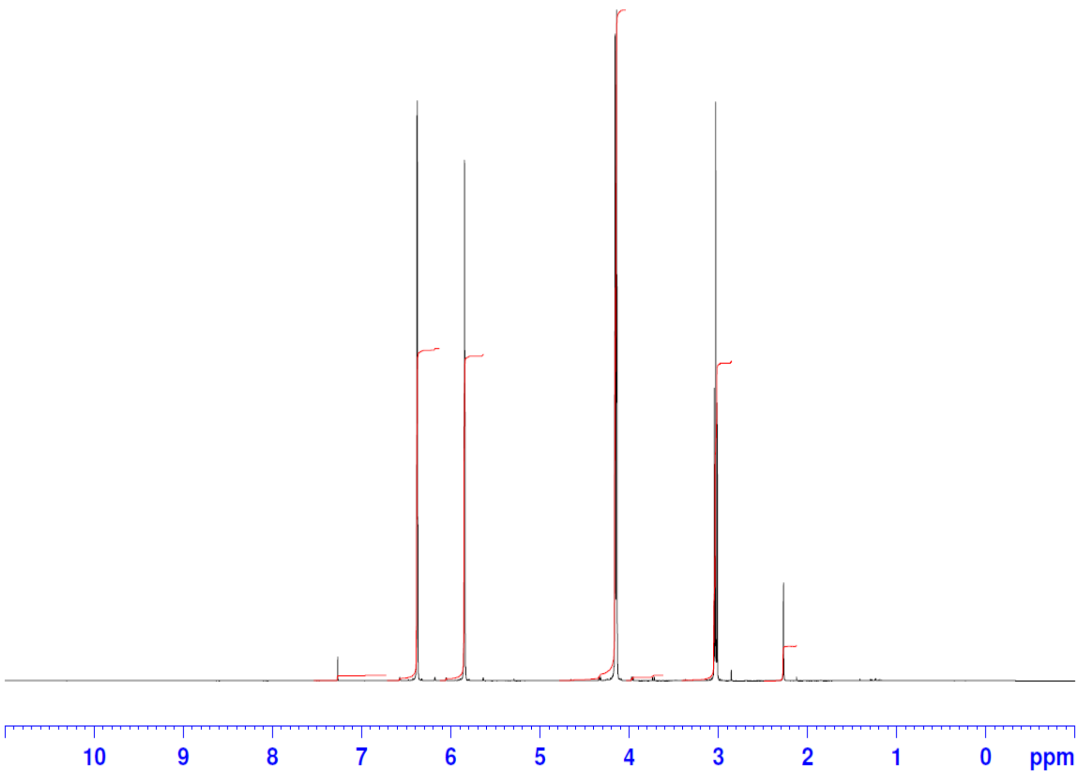

^{13}C NMR (100 MHz, $CDCl_3$)

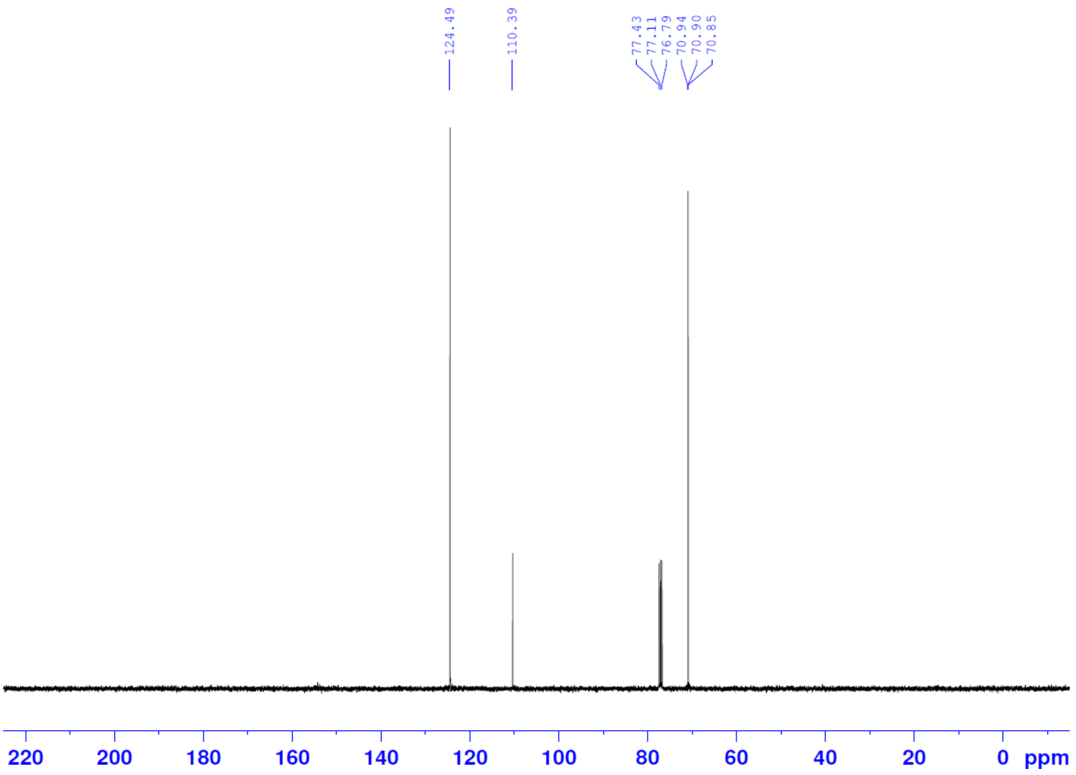

tert-*Butyl((2-iodoallyl)oxy)diphenylsilane* ***12***

^{1}H NMR (400 MHz, $CDCl_3$)

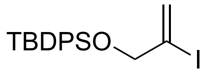

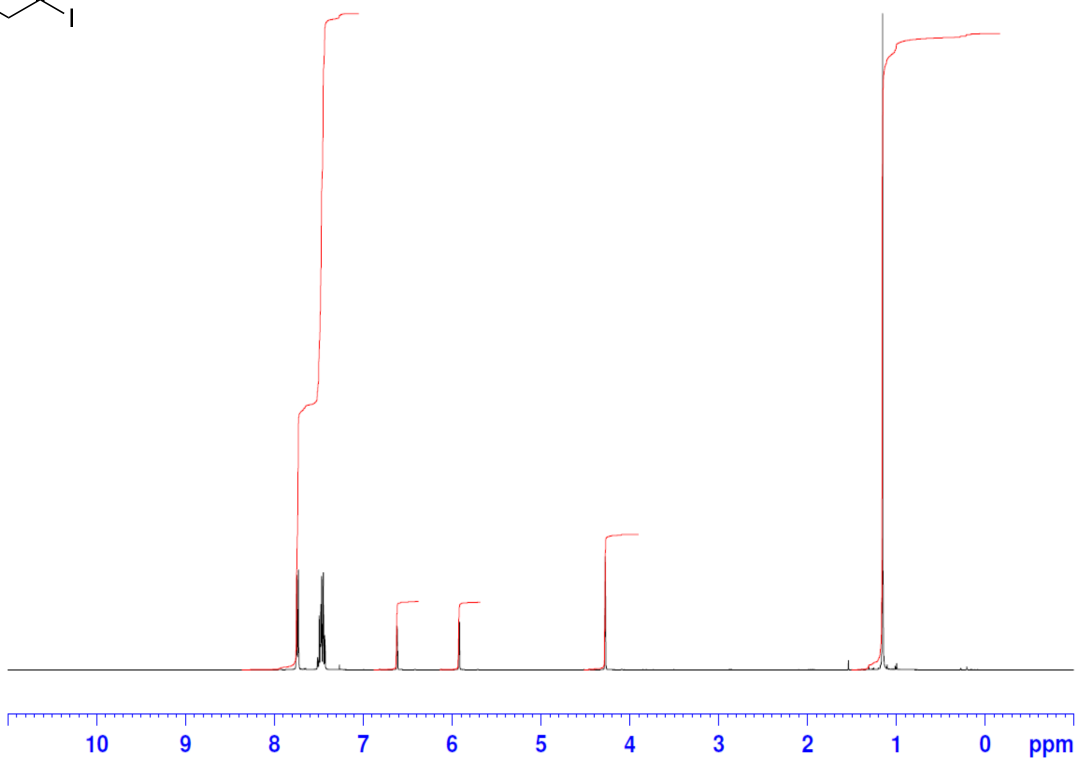

^{13}C NMR (100 MHz, $CDCl_3$)

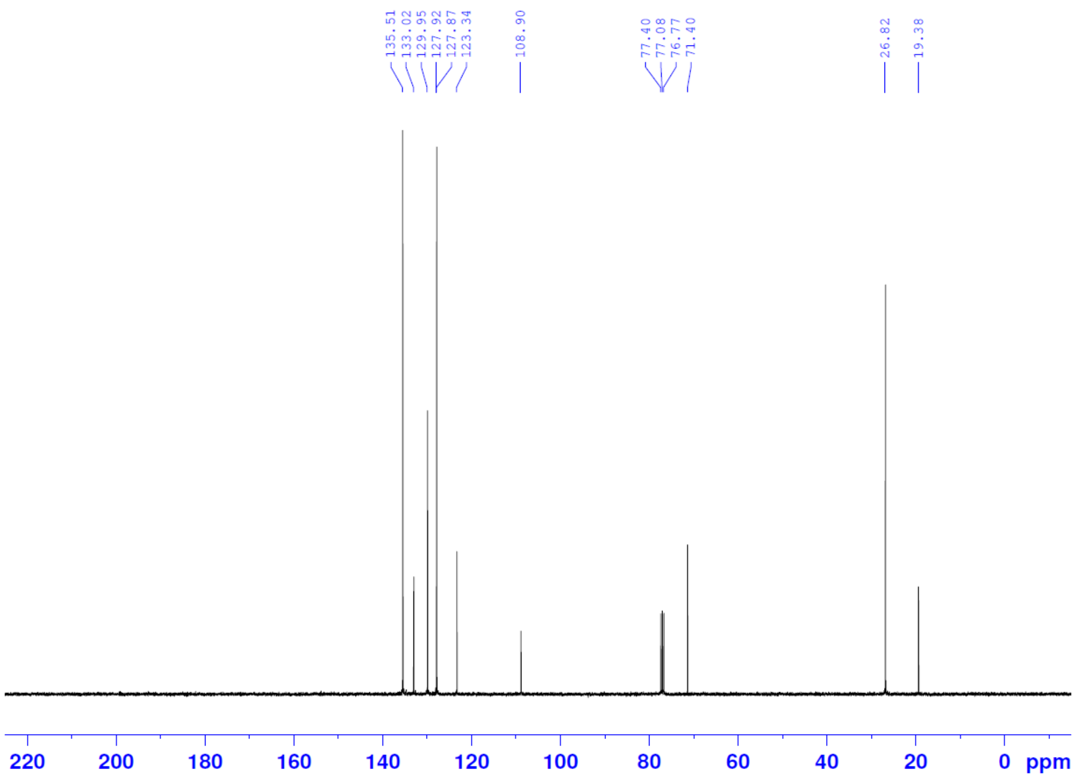

*(E)-6-(((tert-Butyldiphenylsilyl)oxy)methyl)-3-isopropylhepta-4,6-dien-1-yl 4-methylbenzenesulfonate (±)-**13***

^{1}H NMR (400 MHz, C_6D_6)

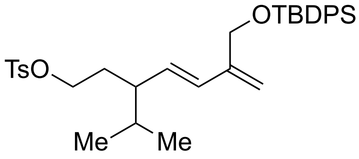

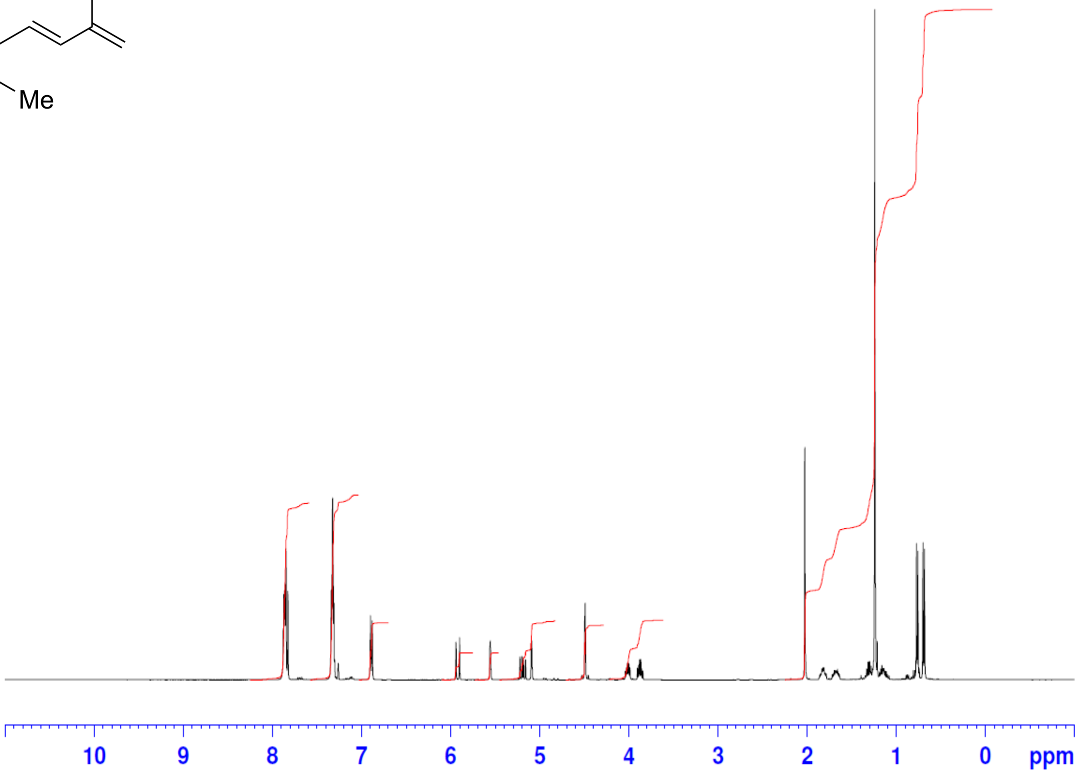

^{13}C NMR (100 MHz, C_6D_6)

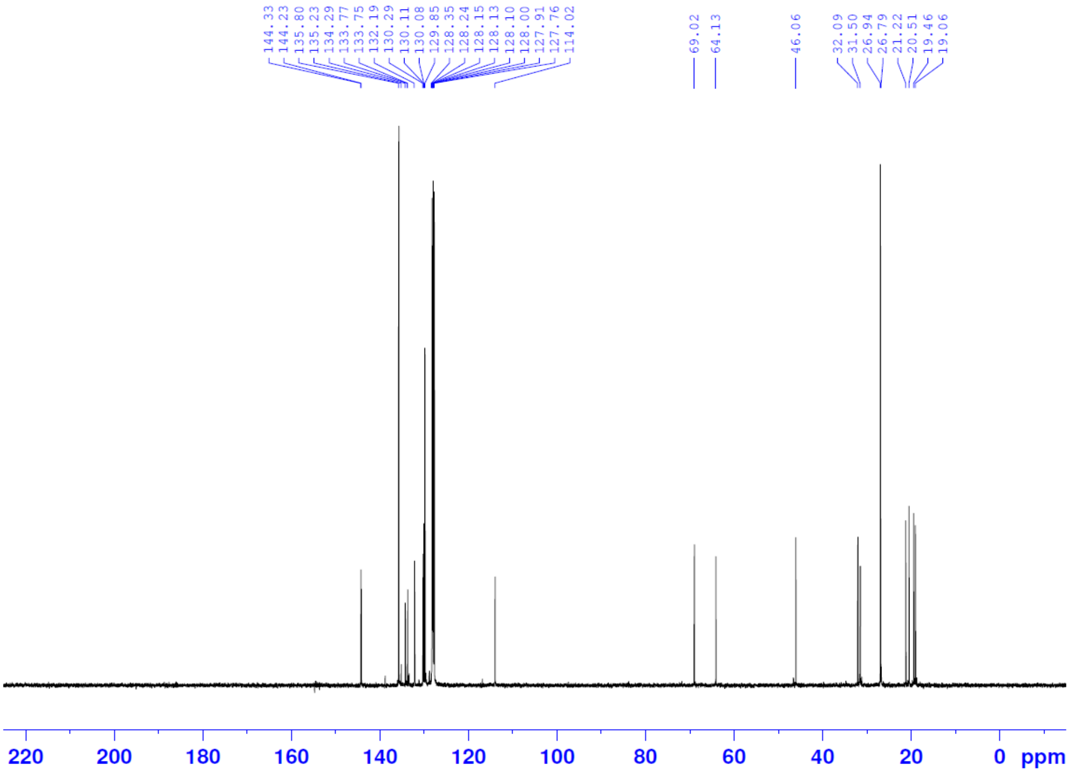

*(E)-Dimethyl 2-(6-(((*tert*-butyldiphenylsilyl)oxy)methyl)-3-isopropylhepta-4,6-dien-1-yl)malonate (±)-**14***

^{1}H NMR (400 MHz, $CDCl_3$)

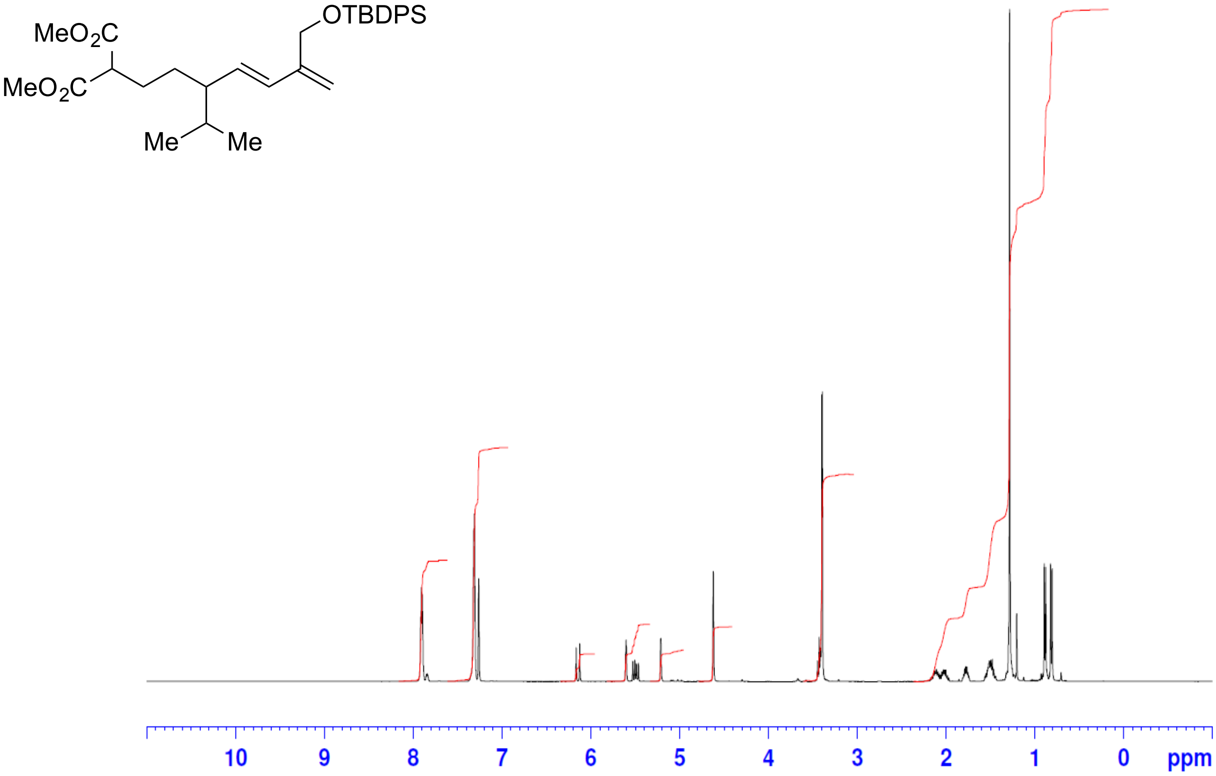

^{13}C NMR (100 MHz, $CDCl_3$)

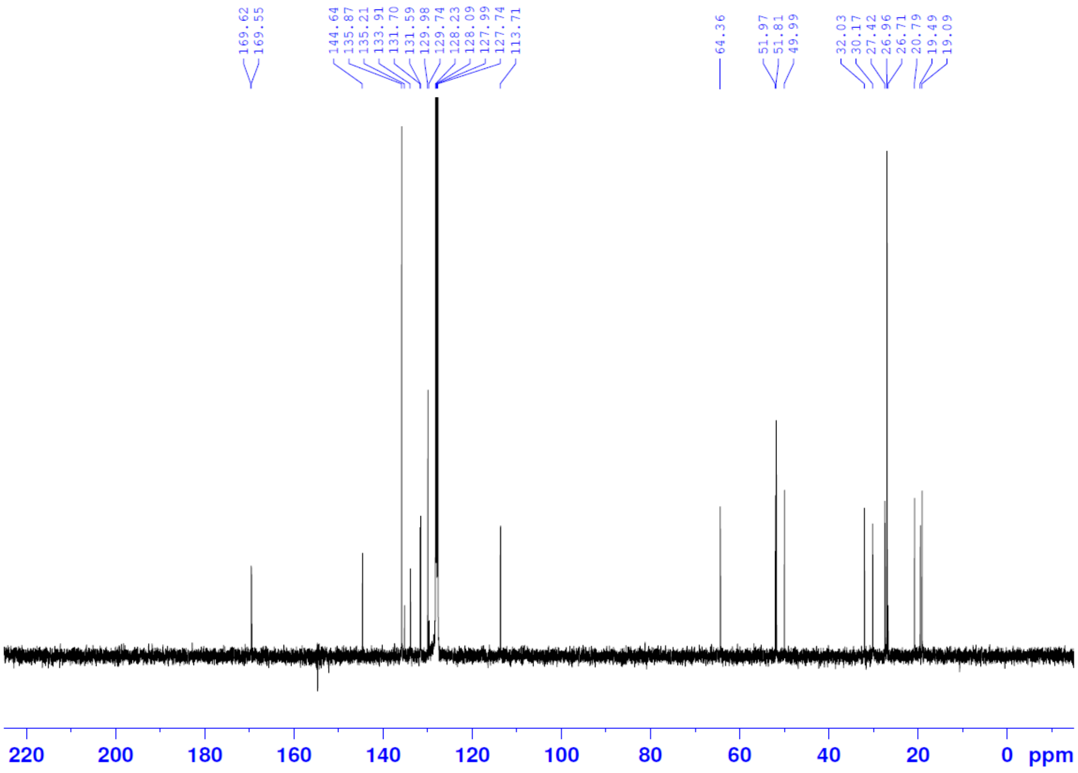

*(1S,3aR,6S,6aS)-Methyl 1-(3-((tert-butyldiphenylsilyl)oxy)prop-1-en-2-yl)-6-isopropyl-3-oxohexahydro-1H-cyclopenta[c]furan-3a-carboxylate (±)-**15***

^{1}H NMR (400 MHz, $CDCl_3$) – approximately 6:1 mixture of C-1 diastereomers – major diastereomer shown

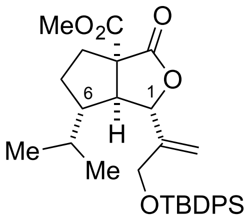

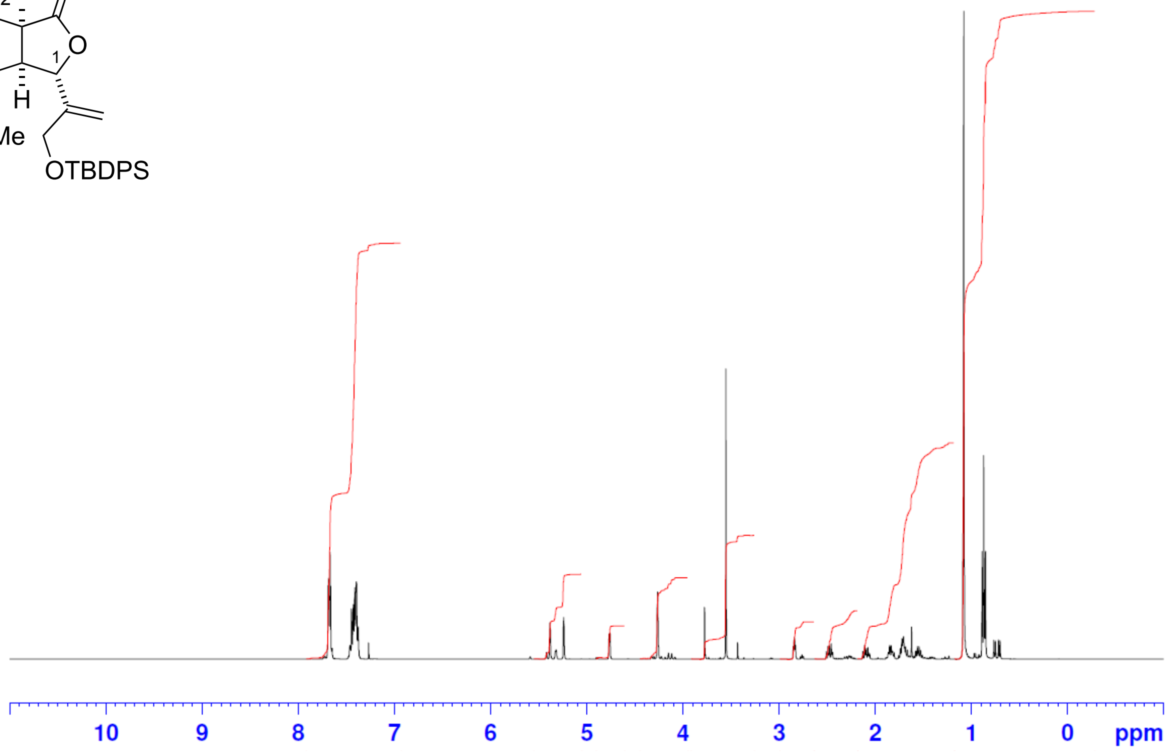

^{13}C NMR (100 MHz, $CDCl_3$)

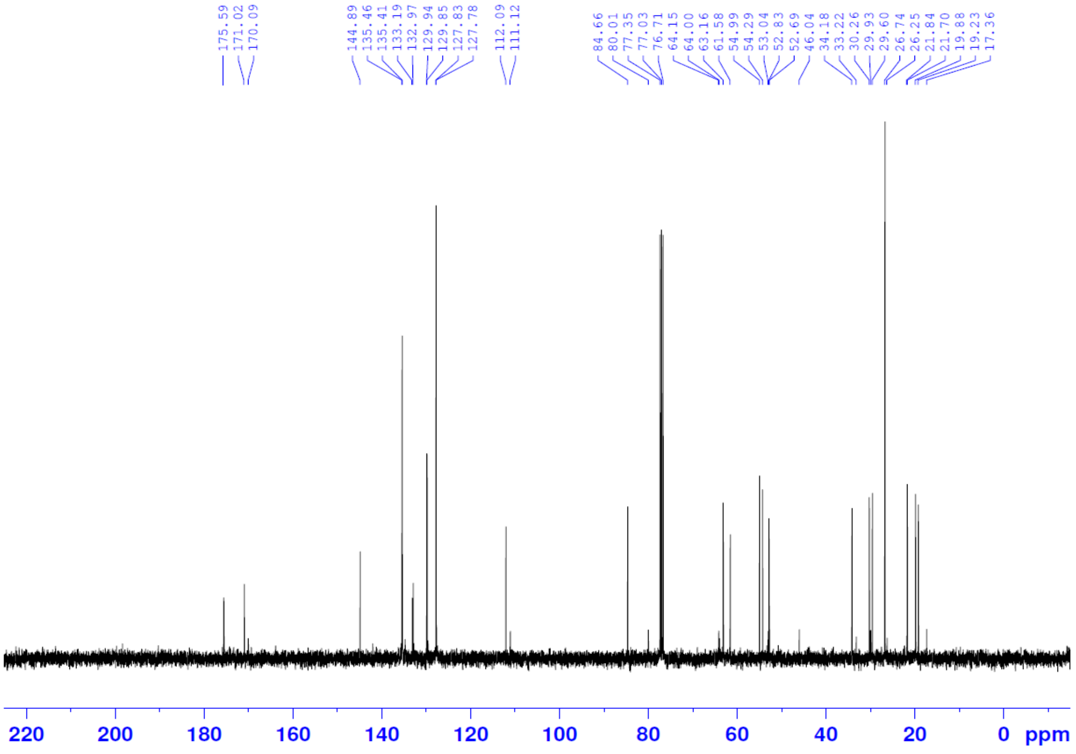

HPLC (Chrial OD column, flow 0.6 ml/min 0.2% IPA/hexane 230 nm) for ee determination

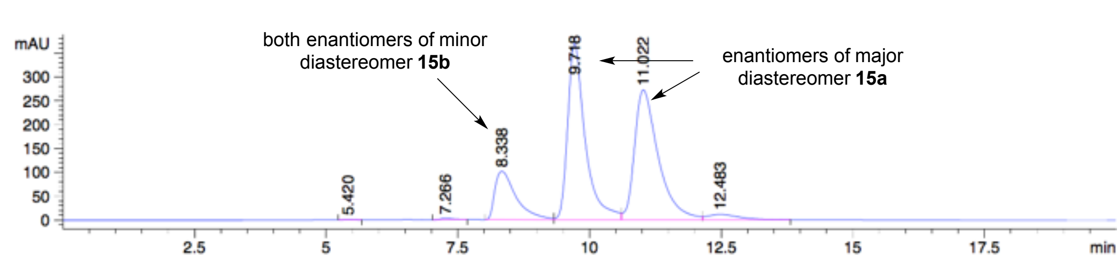

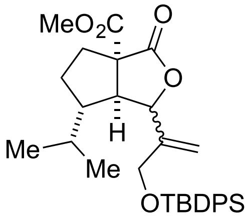

racemic 6:1 mixture of C-1 diastereomers

Signal 4: DAD1 D, Sig=230,16 Ref=360,100

Peak #	RetTime [min]	Type	Width [min]	Area [mAU*s]	Height [mAU]	Area %
1	5.420	BB	0.1850	13.12963	1.04230	0.0639
2	7.266	BB	0.2560	55.13317	3.09191	0.2685
3	8.338	BV	0.4241	2850.15430	101.34048	13.8806
4	9.718	VV	0.3401	8565.09766	369.97781	41.7130
5	11.022	VB	0.4700	8588.18359	272.24109	41.8254
6	12.483	BB	0.6058	461.72046	11.04217	2.2486

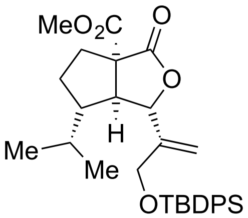

enantiopure 6:1 mixture of C-1 diastereomers

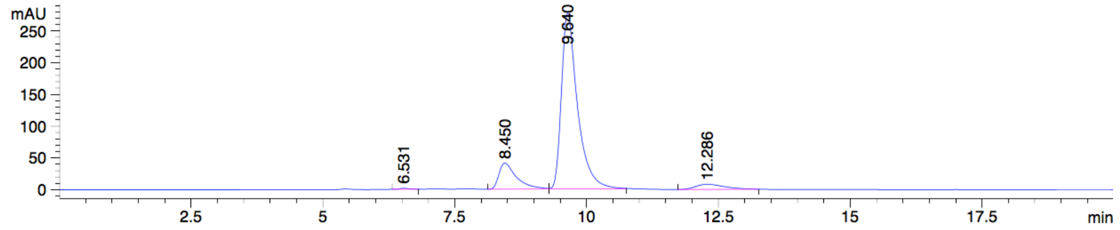

Signal 4: DAD1 D, Sig=230,16 Ref=360,100

Peak #	RetTime [min]	Type	Width [min]	Area [mAU*s]	Height [mAU]	Area %
1	6.531	BB	0.1854	18.34550	1.49245	0.2567
2	8.450	BV	0.3408	959.25812	41.31970	13.4230
3	9.640	VB	0.3106	5859.61670	277.20313	81.9943
4	12.286	BB	0.5735	309.14713	8.02404	4.3259
Totals :				7146.36744	328.03931	

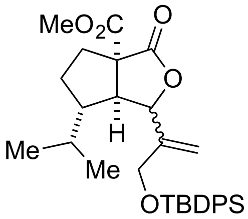

enantiopure spiked with racemic 6:1 mixture of C-1 diastereomers

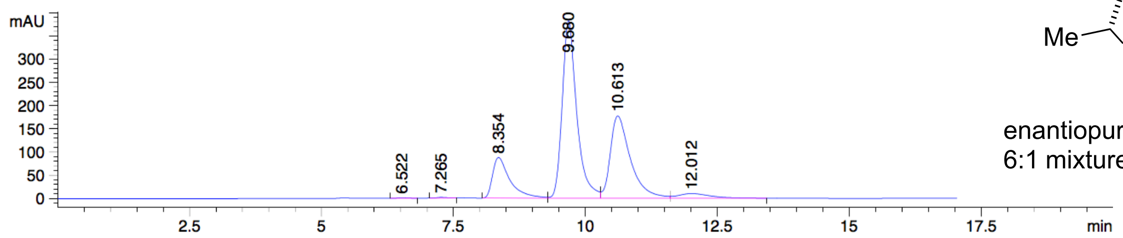

Signal 4: DAD1 D, Sig=230,16 Ref=360,100

Peak #	RetTime [min]	Type	Width [min]	Area [mAU*s]	Height [mAU]	Area %
1	6.522	BB	0.1979	14.45436	1.09550	0.0999
2	7.265	BB	0.2113	21.42728	1.49456	0.1481
3	8.354	BV	0.3348	2003.38013	87.59051	13.8446
4	9.680	VV	0.2834	7316.69092	382.10538	50.5627
5	10.613	VB	0.3926	4688.11719	177.20758	32.3977
6	12.012	BB	0.6167	426.46124	10.05662	2.9471

(3S,3aS*,4S*,6aR*)-3-(3-((tert-Butyldiphenylsilyl)oxy)prop-1-en-2-yl)-4-isopropylhexahydro-1H-cyclopenta[c]furan-1-one (±)-**16a***

^{1}H NMR (500 MHz, $CDCl_3$)

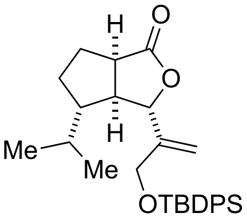

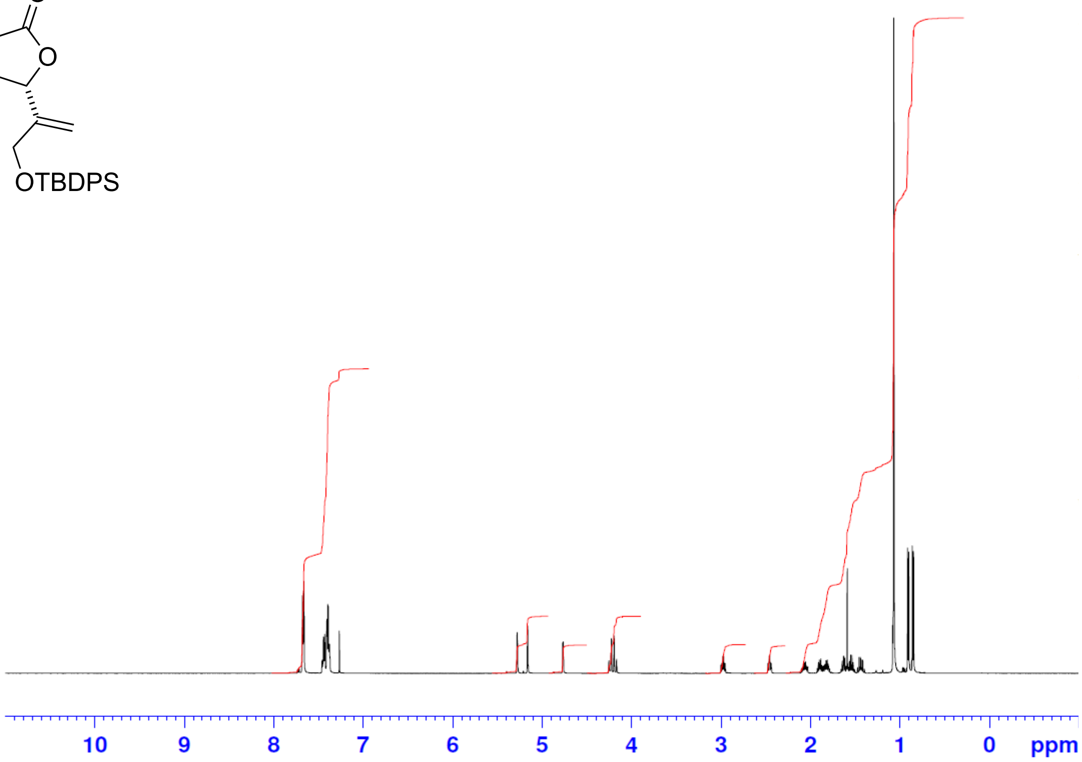

^{13}C NMR (125 MHz, $CDCl_3$)

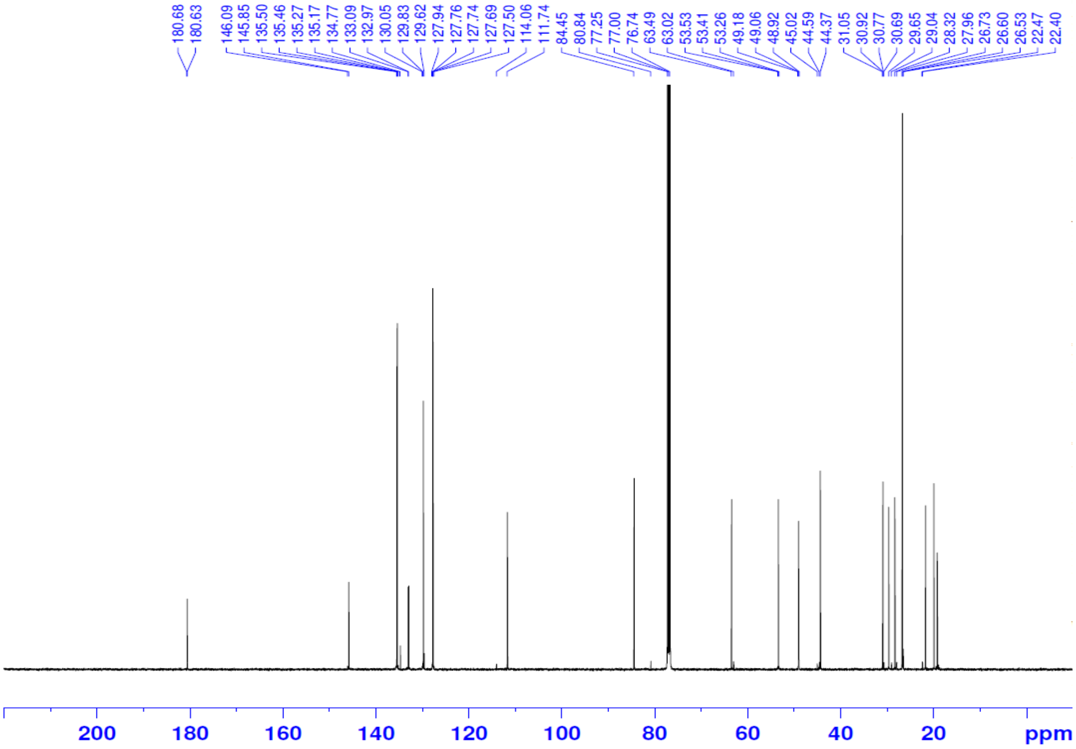

(3R,3aS*,4S*,6aR*)-3-(3-((tert-Butyldiphenylsilyl)oxy)prop-1-en-2-yl)-4-isopropylhexahydro-1H-cyclopenta[c]furan-1-one (±)-**16b***

^{1}H NMR (500 MHz, $CDCl_3$)

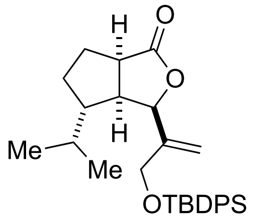

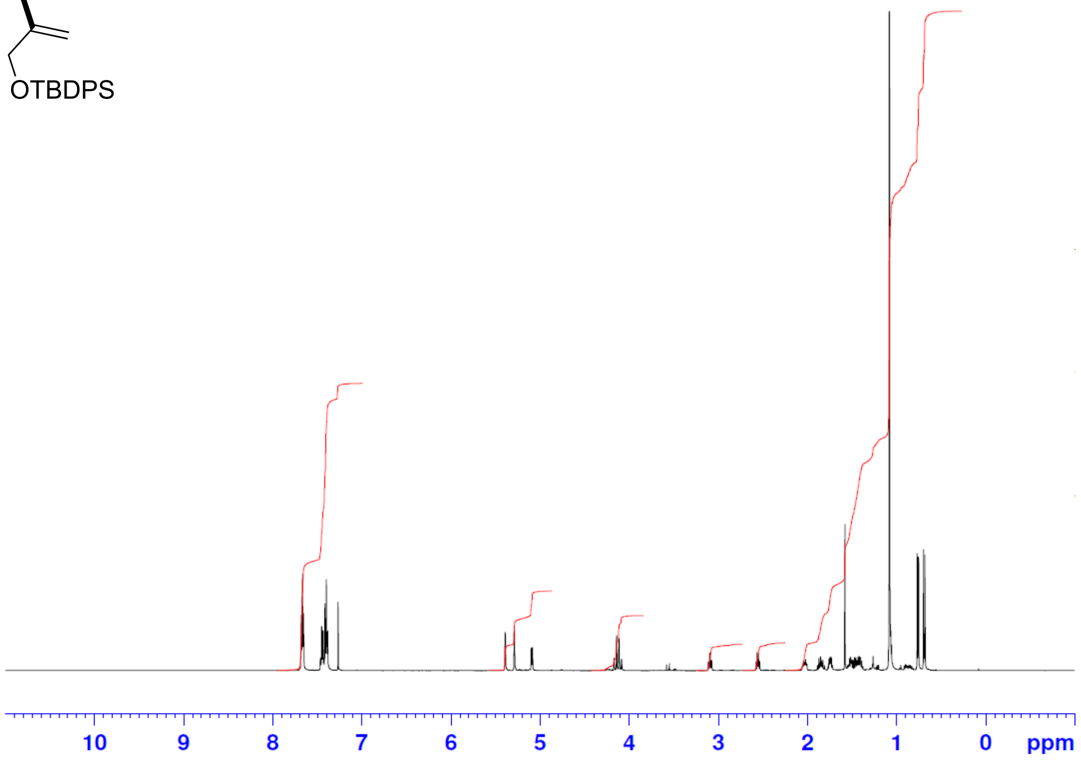

^{13}C NMR (125 MHz, $CDCl_3$)

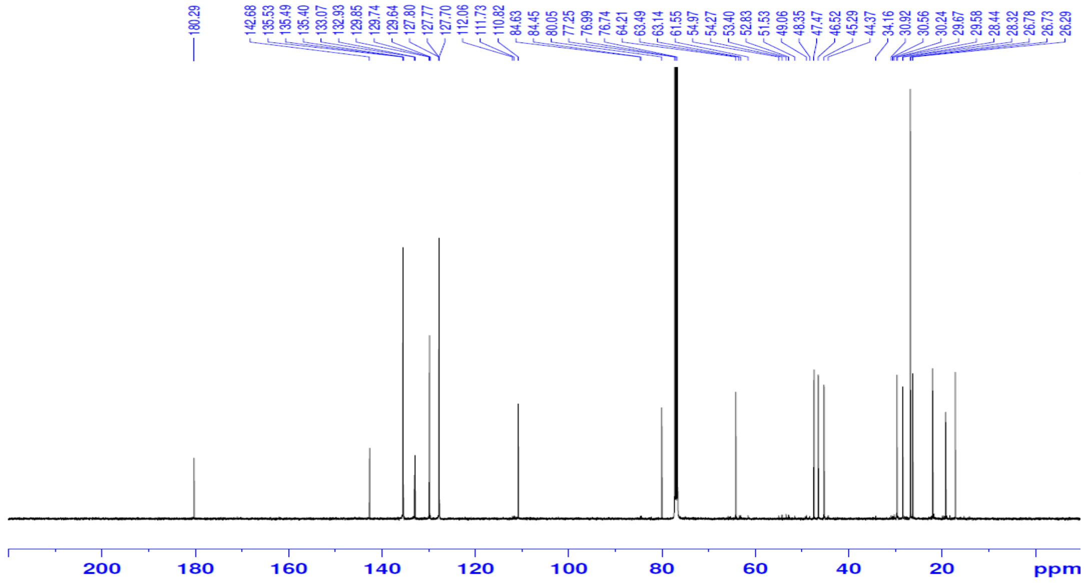

(3S,3aS*,4S*,6aR*)-3-(3-((tert-Butyldiphenylsilyl)oxy)prop-1-en-2-yl)-4-isopropyl-6a-methylhexahydro-1H-cyclopenta[c]furan-1-one (±)-**17***

^{1}H NMR (500 MHz, $CDCl_3$)

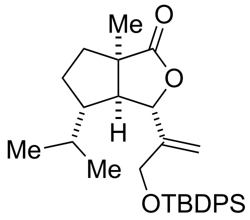

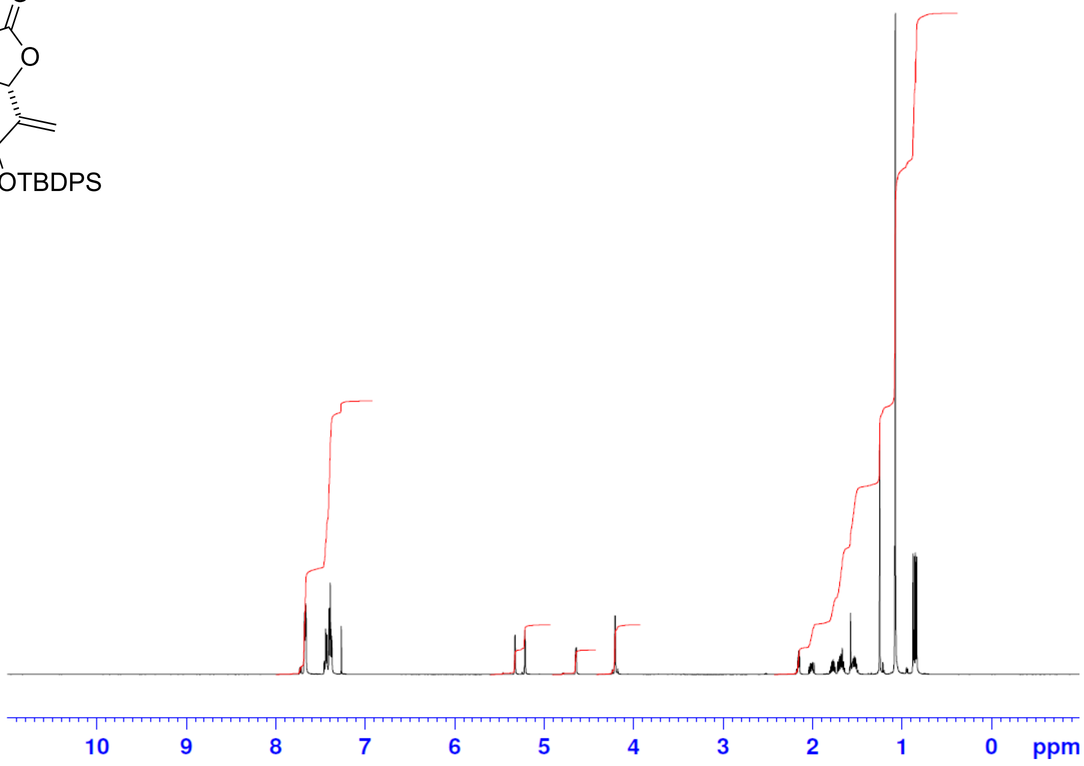

^{13}C NMR (125 MHz, $CDCl_3$)

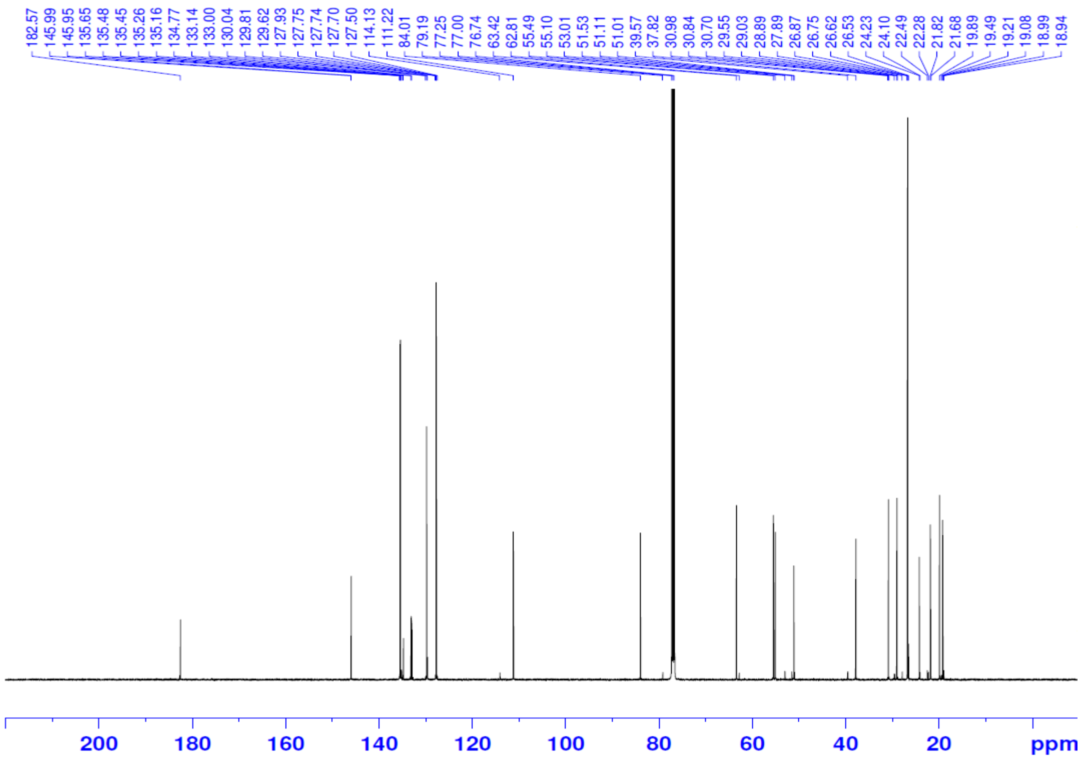

HPLC (Chrial OD column, flow 0.6 ml/min 0.2% IPA/hexane 230 nm) for ee determination .

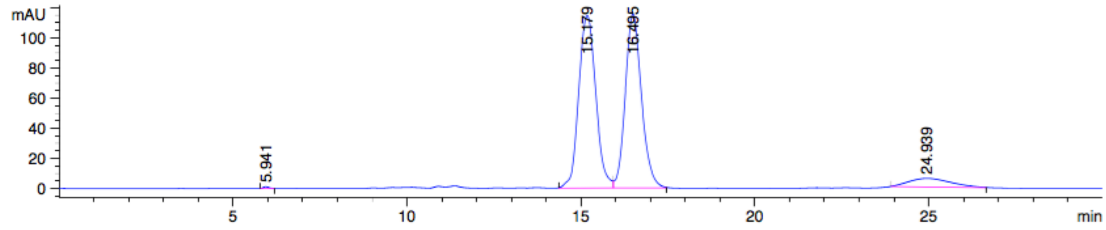

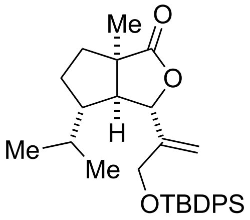

racemic

Signal 4: DAD1 D, Sig=230,16 Ref=360,100

Peak #	RetTime [min]	Type	Width [min]	Area [mAU*s]	Height [mAU]	Area %
1	5.941	BB	0.1489	11.66843	1.18493	0.1433
2	15.179	BV	0.5447	3908.27441	114.37346	48.0119
3	16.495	VB	0.4960	3740.43970	115.44514	45.9501
4	24.939	BB	1.0297	479.83282	5.69092	5.8946

Totals : 8140.21537 236.69445

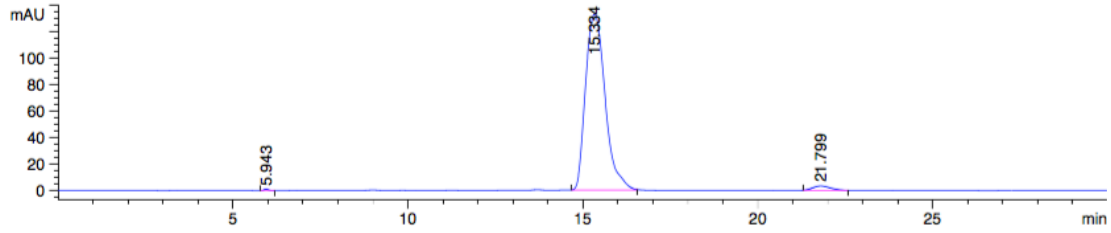

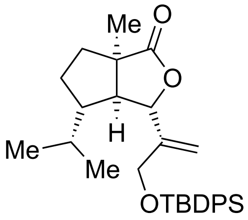

enantiopure

Signal 4: DAD1 D, Sig=230,16 Ref=360,100

Peak #	RetTime [min]	Type	Width [min]	Area [mAU*s]	Height [mAU]	Area %
1	5.943	BB	0.1474	12.02634	1.23749	0.2265
2	15.334	BB	0.6241	5179.21826	133.07001	97.5274
3	21.799	BB	0.5164	119.28030	3.34233	2.2461

Totals : 5310.52491 137.64983

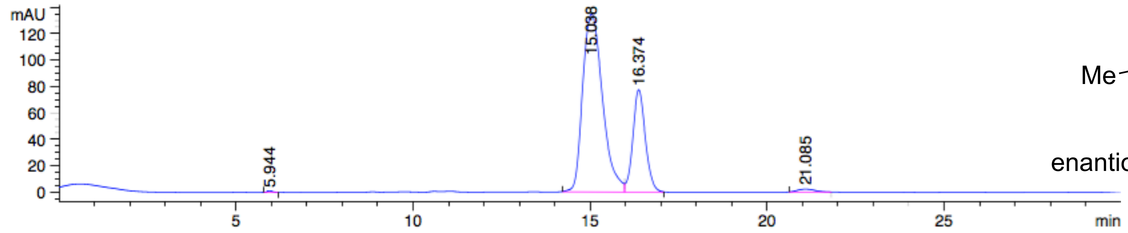

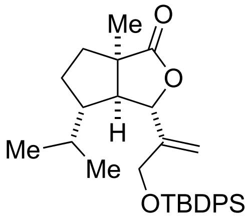

enantiopure spiked with racemic

Signal 4: DAD1 D, Sig=230,16 Ref=360,100

Peak #	RetTime [min]	Type	Width [min]	Area [mAU*s]	Height [mAU]	Area %
1	5.944	BB	0.1519	13.05942	1.27074	0.1800
2	15.038	BV	0.6189	5257.79541	134.84734	72.4575
3	16.374	VB	0.3769	1908.29907	77.59610	26.2982
4	21.085	BB	0.5120	77.22919	2.20836	1.0643

Totals : 7256.38309 215.92253

tert-*Butyl((2-((1*S*,3a*R*,6*S*,6a*S*)-6-isopropyl-3a-methyl-3-methylenehexahydro-1*H*-cyclopenta[*c*]furan-1-yl)allyl)oxy)diphenylsilane (±)-**18***

^{1}H NMR (500 MHz, $CDCl_3$)

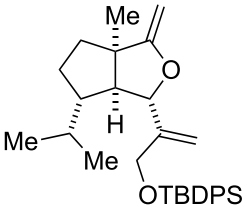

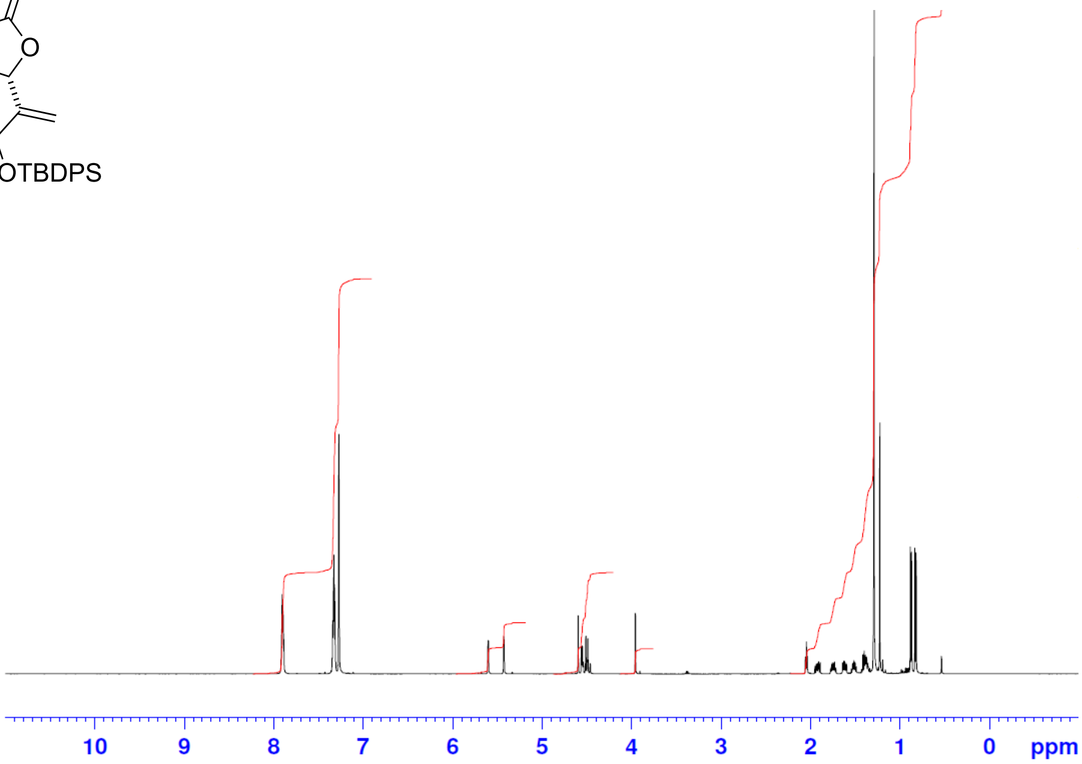

^{13}C NMR (125 MHz, $CDCl_3$)

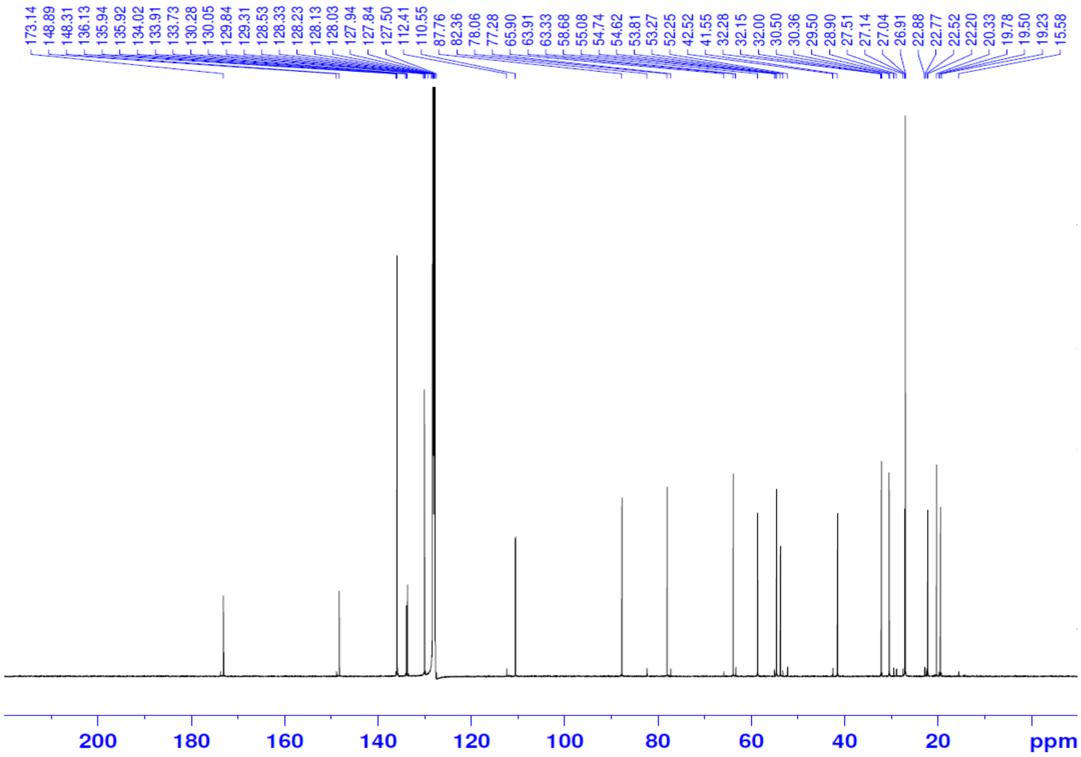

*(1*S*,3a*R*,8a*R*)-7-(((*tert*-Butyldiphenylsilyl)oxy)methyl)-1-isopropyl-3a-methyl-1,3,3a,5,6,8a-hexahydroazulen-4(2*H*)-one (±)-**19***

^{1}H NMR (500 MHz, $CDCl_3$)

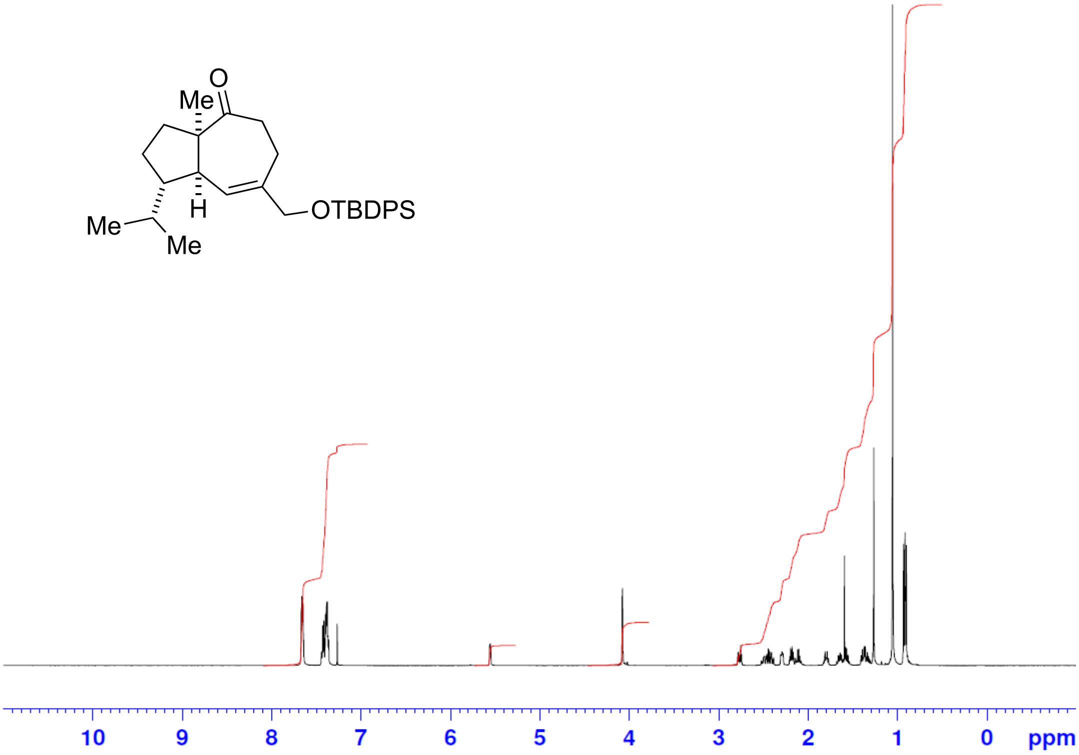

^{13}C NMR (125 MHz, $CDCl_3$)

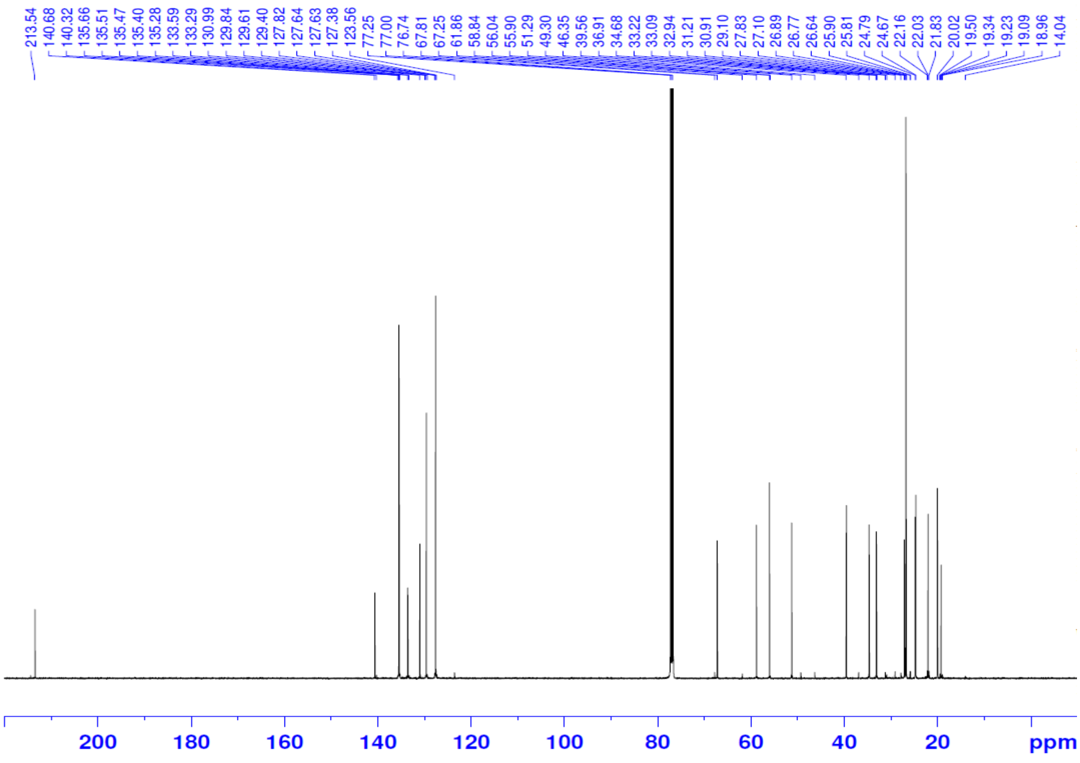

(±)-Aphanamol-I

^{1}H NMR (500 MHz, $CDCl_3$)

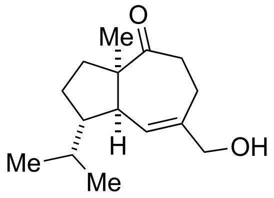

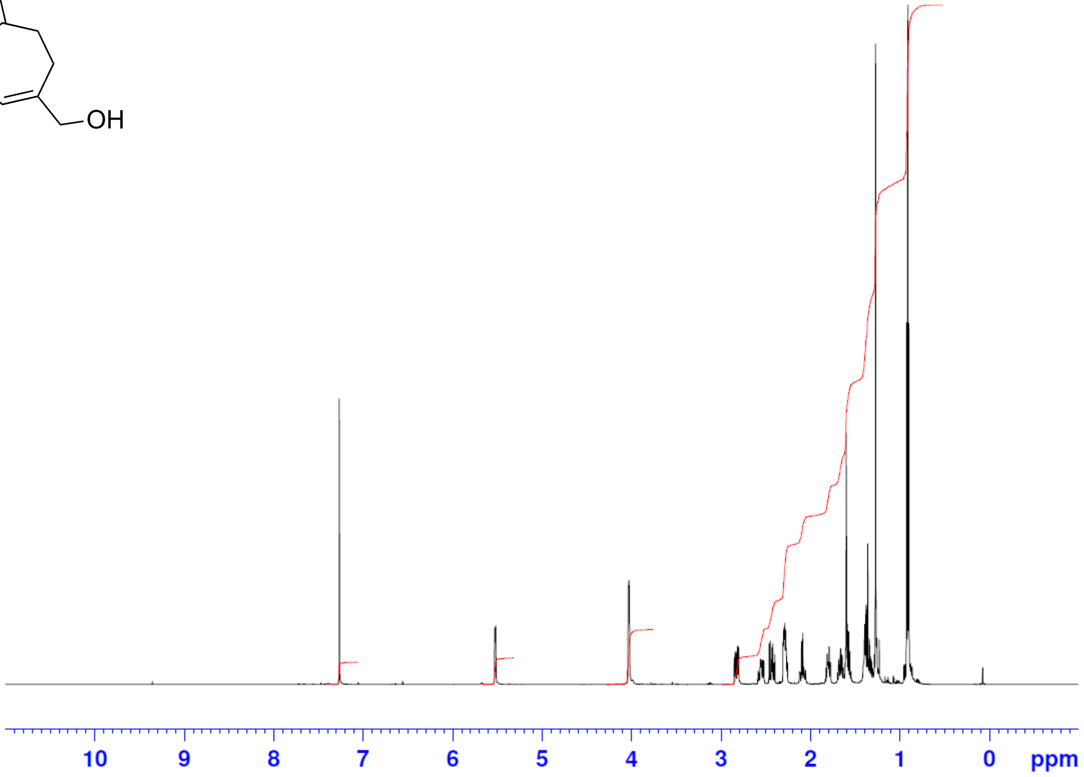

^{13}C NMR (125 MHz, $CDCl_3$)

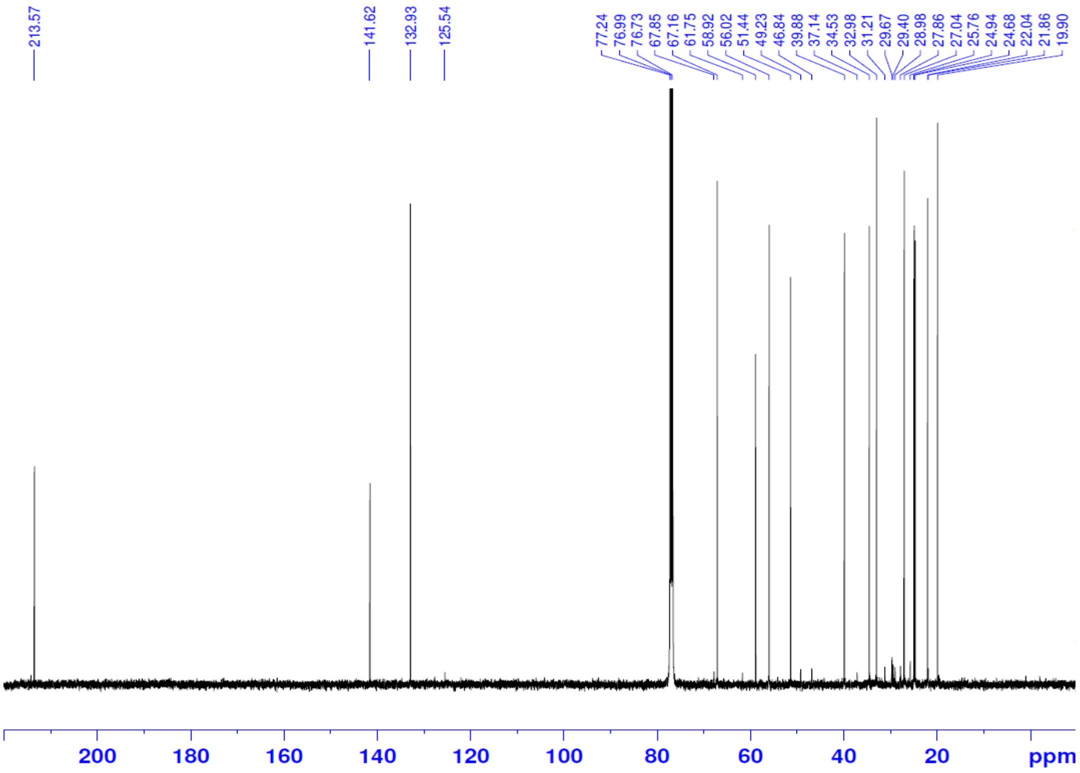

(+)-Aphanamol-I

^{1}H NMR (400 MHz, $CDCl_3$)

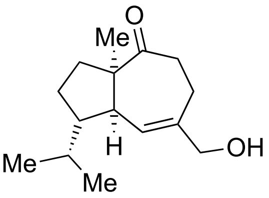

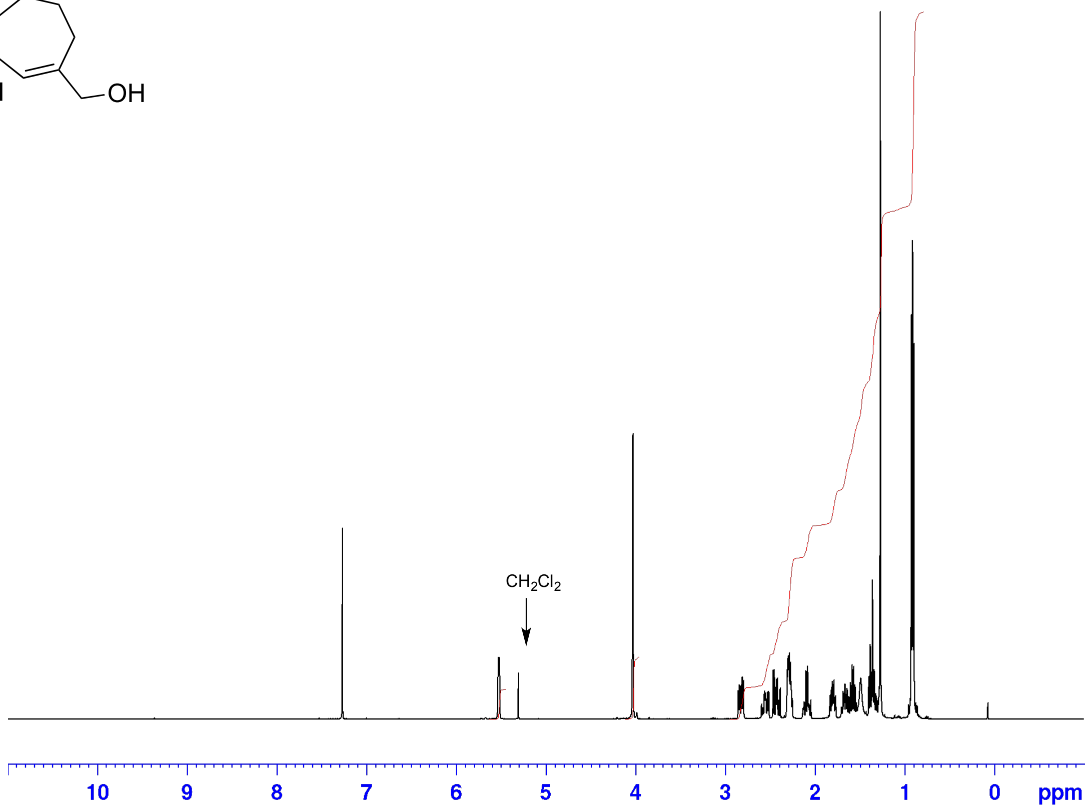

1.4 ^{13}C NMR comparison of natural and synthetic aphanamol I

δ_C Natural	δ_C Synthetic	$\Delta\delta C$	δ_C Natural	δ_C Synthetic	$\Delta\delta_C$ ppm
213.8	213.6	0.2	34.6	34.5	0.1
141.8	141.6	0.2	33.0	33.0	0
132.7	132.9	0.2	27.1	27.0	0.1
67.0	67.1	0.1	25.0	24.9	0.1
59.0	58.9	0.1	24.7	24.7	0
56.1	56.0	0.1	22.1	22.0	0.1
51.5	51.4	0.1	19.9	19.9	
40.0	39.9	0.1			

Table Comparison of the ^{13}C NMR data for synthetic aphanamol I (125 MHz, $CDCl_3$) with natural aphanamol I reported by Nishizawa[8] (25 MHz, $CDCl_3$).

1.5 References

[1] We used the Knoevengal procedure descirbed by Cardillo for the preparation of **6** see: G. Cardillo, S. Fabbroni, L. Gentilucci, M. Gianotti, A. Tolomelli, *Synth. Commun.* **2003**, *33*, 1587-1594.

[2] We followed the procedure of Ohno and Tanaka and co-workers who prepared the dibenzylmalonate analogue of (±)-**8**, see: H. Ohno, Y. Takeoka, Y. Kadoh, K. Miyamura, T. Tanaka, *J. Org. Chem.* **2004**, *69*, 4541-4544.

[3] F. Beaufils, F. Dénès, P. Renaud, *Angew. Chem. Int. Ed.* **2005**, *44*, 5273-5275.

[4] T. Nishimura, T. Sawano, T. Hayashi, *Angew. Chem. Int. Ed.* **2009**, *48*, 8057-8059.

[5] S. Irifune, T. Kibayashi, Y. Ishii, M. Ogawa, *Synthesis* **1988**, *5*, 366-369.

[6] P. A. Wender, L. Zhang, *Org. Lett.* **2000**, *2*, 2323-2326.

[7] T. Hansson, B. Wickberg, *J. Org. Chem.* **1992**, *57*, 5370-5376.

[8] M. Nishizawa, A. Inoue, Y. Hayashi, S. Sastrapradja, S. Kosela, T. Iwashita, *J. Org. Chem.* **1984**, *49*, 3660-3662.
